# Domestication of Tartary Buckwheat Shaped a Regulatory Module for Seedling Salt Tolerance by Targeting the Magnesium Transporter Gene *FtMGT2*

**DOI:** 10.1002/advs.202511570

**Published:** 2025-11-25

**Authors:** Xiang Lu, Yuqi He, Wenfeng Weng, Zebin Liu, Yuanfen Gao, Yaliang Shi, Wei Li, Dili Lai, Mengyu Zhao, Rintu Jha, Hui Zhao, Guangsheng Li, Chaonan Guan, Shuai Shao, Jingjun Ruan, Sun Hee Woo, Yinan Ouyang, Muriel Quinet, Milen I. Georgiev, Alisdair R. Fernie, Congcong Hou, Kaixuan Zhang, Xu Liu, Meiliang Zhou

**Affiliations:** ^1^ State Key Laboratory of Crop Gene Resources and Breeding/Institute of Crop Sciences Chinese Academy of Agricultural Sciences Beijing 100081 China; ^2^ College of Biology and Agriculture Zunyi Normal University Zunyi 563099 China; ^3^ College of Life Sciences Capital Normal University Beijing 100048 China; ^4^ College of Agriculture Sichuan Agricultural University Chengdu 611130 China; ^5^ State Key Laboratory of Bioreactor Engineering East China University of Science and Technology Shanghai 200237 China; ^6^ College of Agriculture Guizhou University Guiyang 550025 China; ^7^ Department of Agronomy Chungbuk National University Cheongju 28644 South Korea; ^8^ Groupe de Recherche en Physiologie Végétale (GRPV) Earth and Life Institute‐Agronomy (ELI‐A) Université Catholique de Louvain Croix du Sud 4‐5, boîte L7.07.13 Louvain‐la‐Neuve B‐1348 Belgium; ^9^ Laboratory of Metabolomics Institute of Microbiology Bulgarian Academy of Sciences Plovdiv 4000 Bulgaria; ^10^ Center of Plant Systems Biology and Biotechnology Plovdiv 4000 Bulgaria; ^11^ Department of Root Biology and Symbiosis Max‐Planck‐Institute of Molecular Plant Physiology 14476 Potsdam Germany

**Keywords:** FtMGT2, GWAS, RNA‐seq, salt tolerance

## Abstract

Globally, soil salinization increasingly affects farmland, severely limiting the production of Tartary buckwheat (*Fagopyrum tataricum*). To identify genetic factors for salt tolerance, we analyzed core Tartary buckwheat accessions and utilized differential expression analysis and genome‐wide association studies (GWAS), identifying a key domesticated magnesium transporter protein, FtMGT2. A single nucleotide polymorphism (SNP) genotype (G/A) of a natural variant located in the *FtMGT2* promoter was found to be positively associated with the expression of *FtMGT2* and salt tolerance variation. Mechanistically, the MADS transcription factor FtAGL16 binds the A variant more strongly. FtAGL16 and the MYB transcription factor FtMYB15L co‐regulate *FtMGT2* transcription, with FtMYB15L protein stability strictly controlled by the E3 ubiquitin ligase FtBRG1. Intriguingly, under salt stress, FtAGL16 can compete with FtBRG1 for binding to FtMYB15L, stabilizing and accumulating FtMYB15L. This enhances *FtMGT2* expression, increasing Mg^2+^ flux, which in turn enhances the transport activity of the sodium (Na^+^) transporter FtHKT1. This coordinated action leads to increased Na^+^ efflux and enhanced salt resistance. This study thereby establishes both the theoretical basis and practical application for targeted molecular breeding to enhance plant salt tolerance.

## Graphical abstract

Domestication of Tartary buckwheat is selected for a salt tolerance mechanism involving the magnesium transporter *FtMGT2*. Its expression is controlled by the FtAGL16‐FtMYB15L module, which is stabilized under salt stress through a competitive interaction that blocks its degradation by the E3 ligase FtBRG1, ultimately boosting Na⁺ efflux and plant resilience.

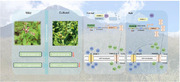

## 1 Introduction

Crop domestication was a pivotal event in the development of our agriculture‐based civilization.^[^ ^1^ ^]^ Previous studies indicated that human‐driven selection factors typically led to higher crop yields following domestication.^[^ ^2^ ^]^ However, the content of secondary metabolites, such as hormones and flavonoids, in crops was frequently diminished during this process.^[^ ^3^, ^4^ ^]^ Concomitantly, this reduction in secondary metabolites often compromises crop resistance to abiotic and biotic stresses, leading to significantly lower yields when crops are exposed to natural disasters.^[^ ^4^, ^5^ ^]^ Soil salinization represents a pervasive abiotic stress that affects crop growth and productivity and currently seriously threatens food security and sustainable agriculture. The accumulation of sodium chloride (NaCl) in saline soil induces several deleterious effects in plants, including osmotic stress, chlorophyll degradation, reduced seed germination, increased ion toxicity, and oxidative stress.^[^ ^6^ ^]^ Tartary buckwheat exhibits exceptional environmental adaptability to abiotic stresses such as drought, cold, and ultraviolet radiation,^[^ ^7^ ^]^ which allows it to flourish in semi‐arid or arid regions and mountain areas.^[^ ^8^, ^9^ ^]^ Nevertheless, many areas where Tartary buckwheat grows are additionally significantly impacted by soil salinization.^[^ ^10^ ^]^ Moreover, salt stress negatively affects the flavonoid content of Tartary buckwheat, which is an important nutritional component of this crop.^[^ ^11^, ^12^ ^]^ Therefore, elucidating the molecular mechanisms underlying the salt stress response in Tartary buckwheat is of critical importance.

Plants have developed various mechanisms to cope with salt stress, including activating signal transduction, reprograming plant metabolism, and altering ion channel permeability.^[^ ^13^ ^]^ The maintenance of sodium (Na^+^) and potassium (K^+^) homeostasis, regulated by their corresponding transporters, is crucial for plant survival in saline soils.^[^ ^14^ ^]^ Specifically, the magnesium transporter (MGT, also known as Mitochondrial RNA splicing 2, MRS2) was found to not only regulate the transport of magnesium,^[^ ^15^, ^16^, ^17^, ^18^, ^19^ ^]^ the essential cofactor of many photosynthetic enzymes,^[^ ^20^ ^]^ but also modulate sodium ion concentrations in the aerial parts of plants.^[^ ^21^, ^22^ ^]^ Furthermore, Mg^2+^ transport is known to be regulated by transcription factors, such as MYB and MADS.^[^ ^23^, ^24^, ^25^, ^26^ ^]^ Similarly, previous research has demonstrated that E3 ubiquitin ligases can regulate the plant response to salt stress via interaction with transcription factors.^[^ ^27^, ^28^, ^29^, ^30^ ^]^ For example, the E3 ligase—MYBc stress‐related RING finger protein (OsMSRFP)—interacts with and ubiquitinates OsMYBs, attenuating OsMYBc mediated high‐affinity K^+^ transporter *OsHKT1;1* expression, and thereby regulating the response to salt stress.^[^ ^14^ ^]^ However, the mechanism by which E3 ubiquitin ligase‐mediated transcription factor degradation regulates target gene expression in the Tartary buckwheat response to salt stress remains to be elucidated.

In the present study, we used a combination of salt responsive transcriptomics and genome‐wide association studies (GWAS), we identified a magnesium transporter gene, *FtMGT2*, which is involved in the Tartary buckwheat response to salt stress and underwent selection during Tartary buckwheat domestication. This comprehensive analysis reveals the molecular mechanism that precisely regulates *FtMGT2* expression under both normal and salt stress conditions. These findings uncover a novel regulatory network for the salt stress response, providing a theoretical basis for breeding high salt tolerance varieties in Tartary buckwheat and other crops.

## 2 Results

### 2.1 Natural Variation on the Promoter of *FtMGT2* Is Responsible for Salt‐Tolerance Variation in Tartary Buckwheat Accessions

**Figure 1:**
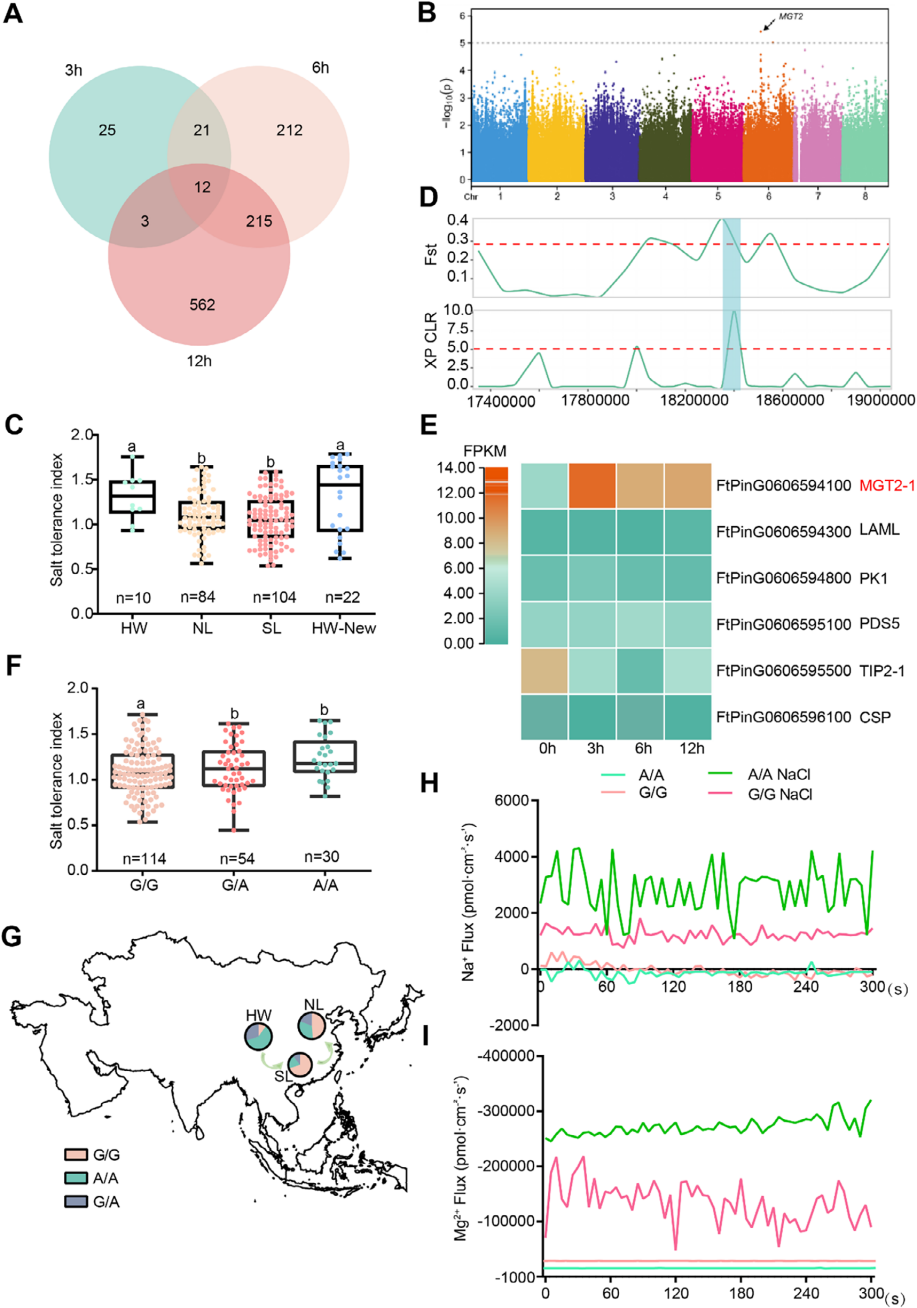
The integration of GWAS and differential expression analysis identified the *FtMGT2* gene associated with salt tolerance. A) Venn diagram of upregulated genes among three treatment groups (NaCl 3 h, NaCl 6 h, and NaCl 12 h) and the control group. B) Manhattan plot of association analysis on the salt‐tolerance index. C) The box plots illustrate the salt tolerance index for the three accessions, HW: Himalayan Wild accessions, NL: North Landrace accessions, SL: Southwest Landrace accessions. HW + New: the 22 new Himalayan Wild accessions. D) The selective sweeps on chromosome 6 identified through comparisons between HW and SL using *F_ST_* and XP‐CLR, the section above the dashed line delineates the intervals that are amenable to domestication, while the portion within the blue‐green box denotes the segment of the domestication significant interval that aligns with the GWAS significant interval. E) Heatmap showing candidate genes within significant loci that are associated with salt tolerance. F) Box plots showed a significant difference among the three genotypes, G/G: genotype G, G/A: genotype G/A, A/A: genotype A. G) Distribution of three genotypes in different groups. H) Na^+^ fluxes measured from the root of different Tartary buckwheat groups. I) Mg^2+^ fluxes measured from the root of different genotypes. Data in (C) and (F) are presented as the mean ± SD. Each data point on the plot indicates the number of replicates. Statistical analysis was performed using one‐way ANOVA analysis with Tukey's HSD test (Different letters represent significant differences at *P* < 0.05).

To comprehensively evaluate the salt tolerance of our diverse accessions, we first established appropriate screening conditions by testing two agronomically relevant levels of salinity: 100 × 10^−3^ and 200 × 10^−3^ m NaCl. These concentrations correspond to soil electrical conductivity values of ≈10 dS m^−1^ (“strongly saline”) and 20 dS m^−1^ (“very strongly saline”), respectively, thus representing both moderate and severe stress conditions found in agricultural settings. Our preliminary tests indicated that while both concentrations allowed for the differentiation of phenotypes, the 100 × 10^−3^ m NaCl treatment induced a discernible stress response, whereas the 200 × 10^−3^ m NaCl treatment imposed a much more severe, near‐inhibitory level of stress. Therefore, we utilized both concentrations for the large‐scale accessions screening to capture a comprehensive spectrum of tolerance, from moderate to high‐level resistance. For subsequent, detailed molecular and gene function analyses, the 100 × 10^−3^ m NaCl concentration was selected as it represents a significant but sub‐lethal stress level, ideal for investigating the underlying regulatory mechanisms (Figure S1, Supporting Information). To identify salt‐responsive genes in buckwheat, we selected the salt‐tolerant Tartary buckwheat cultivar “Pinku 1” (sx147 in Table S2, Supporting Information) due to the availability of its high‐quality reference genome. We performed a time‐course differential expression analysis on “Pinku 1” seedlings subjected to 100 × 10^−3^ m NaCl treatment, with samples collected at 3, 6, and 12 h. A total of 1050 genes were significantly differentially expressed in at least one time point following NaCl treatment (**Figure** **1**A; Figure S2 and Table s1, Supporting Information). Gene Ontology analysis revealed that many differentially expressed genes are involved in membrane components (Figure S3, Supporting Information), and Kyoto Encyclopedia of Genes and Genomes analysis revealed that they are mainly enriched in plant hormone signal transduction and biosynthesis of secondary metabolites (Figure S3, Supporting Information). This suggests that genes involved in membrane component, plant hormone signaling and secondary metabolites biosynthesis are critical for Tartary buckwheat response to salt stress. Further analysis revealed 45, 158, and 318 genes were significantly upregulated following treatment for 3, 6, and 12 h, respectively (Table S1, Supporting Information). By contrast, 16, 302, and 474 genes were significantly downregulated after treatment for 3, 6, and 12 h, respectively (Table S1, Supporting Information). We postulate that these differentially expressed genes might play critical roles in the plant response to salt stress.

To further refine the candidate genes identified through differential expression analysis, we aimed to identify specific genomic regions associated with salt resistance in Tartary buckwheat by combining our transcriptomic data with a GWAS. A globally collected panel of 198 Tartary buckwheat accessions^[^ ^5^ ^]^ (including 10 Himalayan Wild accessions, HW; 84 North Landraces accessions, NL; 104 Southwest Landraces accessions, SL), collected from all over the world, was then employed for salt tolerance evaluation (Table S2, Supporting Information). A coefficient of variation of 25.0% was observed for salt tolerance, highlighting the substantial phenotypic variation for this trait within the Tartary buckwheat accession collection. Based on this analysis, we identified 13 salt‐tolerant genotypes (salt tolerance index > 1.5) and 14 salt‐sensitive genotypes (salt tolerance index < 0.7). The identified salt‐tolerant accessions can be further used as valuable germplasm for salt tolerance breeding in Tartary buckwheat.

We next performed a GWAS using the genotypic variation of 198 Tartary buckwheat accessions (identified in the previous research^[^ ^5^ ^]^) and salt tolerance data evaluated in this study, This analysis aimed to identify key candidate genes underlying Tartary buckwheat salt tolerance. A locus on chromosome 6, harboring 10 genes, was found to be associated with salt tolerance in Tartary buckwheat (Figure 1B and Table S3, Supporting Information). Consistent with previous research indicating that stress resistance generally decreased after crop domestication.^[^ ^31^ ^]^ Therefore, the salt tolerance of various Tartary buckwheat groups was further analyzed (Figure 1C), this analysis indicated a significant difference in salt tolerance among the populations, with HW group exhibiting a higher mean tolerance level than the cultivated SL and NL groups. This overall trend was evident despite the significant heterogeneity observed within each population. To mitigate the potential influence of the limited number of HW populations, we included an additional set of 22 HW materials for analysis related to domestication by using *F_ST_* and XP‐CLR (Cross Population Composite Likelihood Ratio) between HW and SL. These studies identified genomic regions situated in the top 5% of the XP‐CLR value distribution which displayed 221 selective sweeps harboring 2259 genes in the comparison between HW and SL (Tables S4–S6, Supporting Information). This observation suggests a decrease in salt tolerance that occurred during the domestication of Tartary buckwheat.

We further analyzed this genetic differentiation to determine if these genes were responsible for the decreased salt tolerance. A total of six genes within the GWAS‐associated locus overlapped with a selective sweep region (HW vs SL), indicating that these genes likely played a role in the reduced salt tolerance observed during domestication (Figure 1D and Tables S4–S6, Supporting Information). Finally, by integrating these GWAS and domestication data with our DEG analysis, we identified a single high‐priority candidate: *FtPinG0606594100*. This gene, which we designated *FtMGT2* (a member of the MGT2 family of magnesium transporter genes), was not only located within the associated GWAS locus and a selective sweep region, but also exhibited significantly increased expression following NaCl treatment (upregulated twofold after 3 h compared to untreated) (Figure 1E and Figure S4A, Supporting Information). This observation suggests that FtMGT2 plays a key role in the initial response to salt stress in Tartary buckwheat.

Analysis of linkage disequilibrium (LD) revealed a rapid decay of LD across the locus (Figure S4B, Supporting Information). While the rate of LD decay was faster than anticipated for a predominantly self‐pollinating species, this pattern provides valuable insights into the genetic architecture of the locus and suggests a history of occasional outcrossing and recombination. After analyzing the genotypes of 198 Tartary buckwheat accessions, six single nucleotide polymorphisms (SNPs) were found to be associated with *FtMGT2* (Figure S4C, Supporting Information). One was a synonymous mutation (A/G) in the coding region, while the other five were in the promoter region. Among these, a G/A transition at position 18327291 on chromosome 6 was most strongly associated with increased salt tolerance (Figure 1F). Analysis of gene expression revealed that accessions with the genotype A exhibited significantly higher *FtMGT2* transcript levels than those with the genotype G (Figure S4D, Supporting Information). Consistent with this expression pattern, genotype A accessions also displayed significantly higher salt tolerance and Mg^2^⁺ content compared to those with the genotype G counterparts (Figure 1F and Figure S4E, Supporting Information). Interestingly, the frequency of genotype A (the salt‐tolerant genotype) in the HW population was significantly higher than that in the NL and SL populations (Figure 1G). Congruently, the Mg^2^⁺ content in the HW population was higher than that in the NL or SL populations (Figure S4F, Supporting Information).

To investigate the physiological mechanisms underlying the differential salt tolerance of Tartary buckwheat, we used noninvasive microtest technology (NMT) to measure Na^+^ flux in the roots of accessions with genotype A and genotype G. Under normal conditions, no significant difference in Na⁺ efflux was observed between the genotypes. In contrast, under salt stress, genotype A accessions exhibits a significantly higher rate of Na⁺ efflux compared to genotype G accessions, indicating a more efficient sodium exclusion mechanism (Figure 1H). Given that FtMGT2 encodes a magnesium transporter, we next measured Mg^2^⁺ flux. Under control conditions, genotype A exhibited a higher rate of Mg^2^⁺ efflux; however, upon exposure to salt stress, this pattern reversed, and genotype A accessions displayed a significant switch to net Mg^2^⁺ influx (Figure 1I). This switch from efflux to influx, which is opposite to the behavior of Na⁺, suggests an antagonistic relationship between Mg^2^⁺ and Na⁺ transport under salt stress. Collectively, these findings suggested that the enhanced ability of genotype A to maintain Na⁺ efflux while simultaneously increasing Mg^2^⁺ influx via FtMGT2 is a key physiological mechanism contributing to its superior salt tolerance.

### 2.2 *FtMGT2* Plays a Positive Role in Plant Resistant to Salt Stress

**Figure 2:**
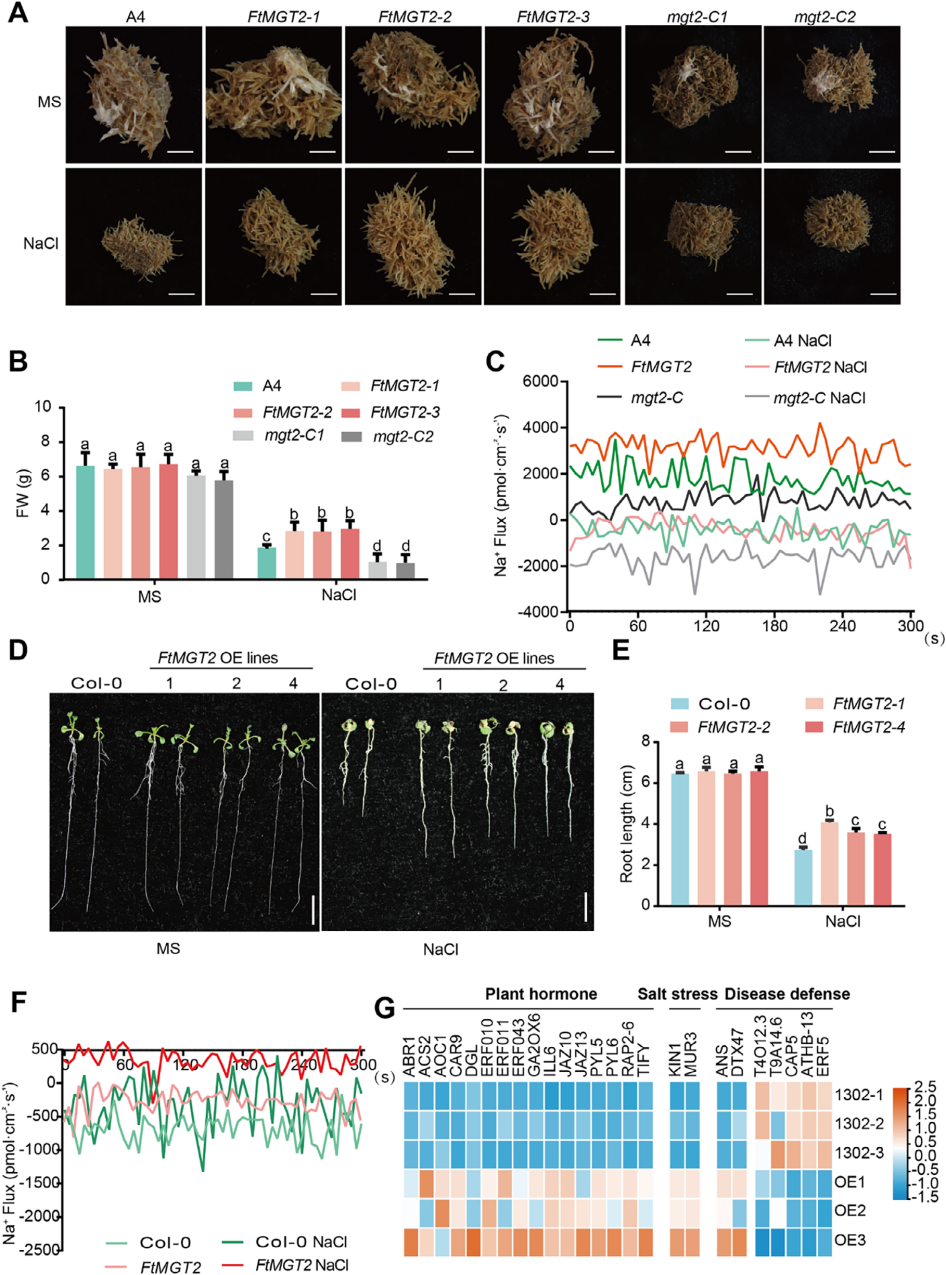
Functional validation of *FtMGT2* transgenic plants under salt stress. A) The phenotype of A4 and *FtMGT2* overexpressing and knockout hairy roots cultured in MS liquid medium (MS) and MS liquid medium + 100 × 10^−3^ m NaCl (NaCl) for 20 d. A4, A4 *Agrobacterium rhizogenes* empty strain hairy roots; *FtMGT2‐1*, *FtMGT2‐2*, and *FtMGT2‐3*, three *FtMGT2* overexpressed hairy root strains, *mgt2‐C1* and *mgt2‐C2*, knockout hairy roots of *FtMGT2*. Bar = 5 mm. B) The fresh weight of A4 and *FtMGT2* transgenic hairy roots in (A). C) Na^+^ fluxes measured from the root of different hairy roots. D) Phenotype of *FtMGT2* overexpression *Arabidopsis* seedlings treated with NaCl. Bar = 1 cm. The phenotype (D), root length (E) of Col‐0 and *FtMGT2* heterologous expression *Arabidopsis* grown for 10 d in MS and MS solid medium containing 100 × 10^−3^ m NaCl. Col‐0, wild‐type *Arabidopsis*, *FtMGT2‐1*, *FtMGT2‐2*, *FtMGT2‐4*, three lines of *FtMGT2* heterologous expression *Arabidopsis*. F) Na^+^ fluxes measured from the root of different *Arabidopsis*. G) The impact of *FtMGT2* heterologous expression on the transcriptome of *Arabidopsis thaliana*, including the expression of reported genes, on plant growth, development, and adversity stress. Data in (B) and (E) are presented as the mean ± SD from *n* = 3 independent biological replicates. Statistical analysis was performed using one‐way ANOVA analysis with Tukey's HSD test (Different letters represent significant differences at *P* < 0.05).

To further validate the function of *FtMGT2* in plants, we then overexpressed and knocked out *FtMGT2* in Tartary buckwheat hairy roots (Figure S5A, Supporting Information), and analyzed their growth under control (MS medium) and salt stress (MS + 100 × 10^−3^ m NaCl) conditions (**Figures** **2**A and S5, Supporting Information). Under basal growth conditions, *FtMGT2* expression was significantly elevated in overexpressed (OE) hairy roots but was barely detectable in knockout hairy roots (Figure S5B, Supporting Information). Compared to controls (infected with *Agrobacterium rhizogenes* A4), the overexpression of *FtMGT2* significantly promoted the hairy roots growth. Following treatment with 100 × 10^−3^ m NaCl, the fresh weight was significantly higher in OE lines (Figure 2B). In addition, the kaempferol content (a key flavonoid known to plays a protective role in plant responses to various abiotic stresses), Mg^2^⁺ absorption capacity and total Mg^2^⁺ content were all significantly higher in OE lines compared to the control (Figures S5C,D and S6A, Supporting Information). Furthermore, the Na⁺ / K⁺ ratio in *FtMGT2* OE hairy root was lower than that of the control (Figure S6B, Supporting Information), and the OE lines exhibited higher Na⁺ efflux and Mg^2^⁺ influx (Figure 2C and Figure S5D, Supporting Information). By contrast, in *mgt2‐C* mutant hairy roots, *FtMGT2* expression was minimal. Correspondingly, these mutant hairy roots exhibited lower fresh weight, kaempferol content, Mg^2+^ content and Mg^2^⁺ uptake efficiency compared to the control. In addition, the Na⁺/K⁺ ratio was higher in *mgt2‐C* hairy roots, which also displayed lower Na⁺ efflux and Mg^2^⁺ influx (Figure 2A–C and Figure S6A,B, Supporting Information). To investigate the effect of salt stress on Mg^2^⁺ uptake efficiency in natural populations, five cultivated accession (SC247, BTN519, BTN542, XZ315, and XZ318) and five wild accessions (GZ261, GZ266, SC105, GS351, and YN480) were selected for Mg^2^⁺ absorption experiments. The results indicated that under salt stress, the Mg^2^⁺ absorption efficiency of the wild accessions was higher than that of the cultivated accessions (Figure S6C and Table S7, Supporting Information). This suggests that increased Mg^2^⁺ uptake may enhance the resilience of Tartary buckwheat to salt stress. Consistent with these findings, Mg^2^⁺ absorption experiments on transgenic hairy root lines demonstrated that OE lines had a greater Mg^2^⁺ uptake capacity than control hairy roots, both with and without salt stress. Conversely, the Mg^2^⁺ uptake efficiency of the knockout hairy roots was significantly lower than that of the control (Figure S6D, Supporting Information).

To isolate the Mg^2+^ transport activity of FtMGT2 from other factors, we first cultured the *FtMGT2* transgenic materials in ultrapure water, or cultured them in ultrapure water supplemented with 50 mM MgSO_4_. Subsequently, we measured the Mg^2+^ flux rates of the different materials using NMT. The results showed that Mg^2+^ absorption in the *FtMGT2* overexpression hairy roots was significantly increased, while the Mg^2+^ absorption in the *mgt2‐C* mutant hairy roots was minimal, with no significant difference observed before and after treatment (Figure S7A, Supporting Information). This indicates that FtMGT2 can transport Mg^2+^ in hairy roots, whereas the *mgt2‐C* mutant has essentially lost its ability to transport Mg^2+^.

Furthermore, to examine the effect of FtMGT2 on Na^+^ transport under salt stress, we treated the *FtMGT2* transgenic hairy roots with either 100 × 10^−3^ m NaCl or a combination of 100 × 10^−3^ m NaCl and 50 × 10^−3^ m MgSO_4_. We then measured the Na^+^ flux rates utilizing NMT. The results indicated no significant differences in Na^+^ flux rates among the *FtMGT2* transgenic materials in the NaCl only treatment. However, when both Na^+^ and Mg^2+^ were present, the Na^+^ efflux in the *FtMGT2* overexpression materials significantly increased. This suggests Mg^2+^ transport activity of FtMGT2 is required to enhance Na^+^ transport (Figure S7B,C, Supporting Information). These findings demonstrate that *FtMGT2* modulates the salt stress response mechanism in Tartary buckwheat by regulating the Mg^2+^ transport.

Moreover, to further investigate the involvement of *FtMGT2* in the regulation of Na⁺/ K⁺ homeostasis, we assessed the expression levels of genes encoding Na⁺/ K⁺ transporters in hairy roots. The results revealed significant differences in the expression of these genes in *FtMGT2* transgenic hairy roots. For instance, gene families such as HKT exhibit high expression levels in *FtMGT2* overexpressing hairy roots, whereas their expression was markedly reduced in the *FtMGT2* knocked‐out hairy roots (Figure S8A, Supporting Information). These observations that *FtMGT2* directly influences the absorption of Mg^2^⁺ in Tartary buckwheat, and that enhanced Mg^2^⁺ absorption can effectively alleviate the salt stress experienced by the Tartary buckwheat. The observation that the overexpression of *FtMGT2* overexpression leads to the upregulation of the *FtHKT1* gene is intriguing. To validate this finding, we treated buckwheat seedlings with 50 mM MgSO_4_ and conducted RNA sequencing. The results showed that Mg^2^⁺ treatment induces expression changes in numerous genes, including the significant upregulation of *FtHKT1* (Figure S8B, Supporting Information). Consequently, we hypothesize that *FtMGT2* regulates the expression of genes such as *FtHKT1* by modulating the intracellular Mg^2^⁺ content, thus providing strong evidence for an indirect regulatory pathway linking Mg^2^⁺ transport to Na⁺/K⁺ homeostasis.

To further explore *FtMGT2* function, we next cloned the homologous gene *FeMGT2* from common buckwheat, and introduced it into Tartary buckwheat hairy roots for salt stress experiments. Compared to the A4 control, hairy roots overexpressing *FeMGT2* exhibited a significantly enhanced salt‐tolerant phenotype (Figure S9A, Supporting Information), manifesting as higher fresh weight (Figure S9B, Supporting Information), and greater kaempferol content (Figure S9C, Supporting Information). This result suggests a functionally conserved role for the MGT2 protein family in mediating salt tolerance across buckwheat species.

Previous research has demonstrated that salt stress induces cellular oxidative damage, including lipid peroxidation. The enzyme activity of superoxide dismutase (SOD) and catalase (CAT) are often used to assess the degree of lipid oxidation damage in plant cells.^[^ ^32^ ^]^ To examine the role of *FtMGT2* in mitigating oxidative damage induced by salt stress, the activity of SOD and CAT were analyzed in *FtMGT2* overexpression and A4 control hairy roots under salt stress conditions. The results demonstrate that the activity of antioxidant enzymes was significantly increased in *FtMGT2* overexpressing hairy roots compared to controls (Figure S10A,B, Supporting Information), suggesting that overexpressing *FtMGT2* improves the capacity to scavenge reactive oxygen species.

As flavonoids were previously found to aid in plant resistance to salt stress and to exhibit antioxidant properties,^[^ ^33^ ^]^ we next analyzed whether flavonoids were involved in *FtMGT2*‐mediated responses. Upon analyzing the flavonoid content of Tartary buckwheat accessions,^[^ ^5^ ^]^ it was observed that genotype A accessions demonstrated elevated kaempferol content compared to genotype G accessions(Figure S11, Supporting Information). Further research illustrated kaempferol could improve salt stress resistance in both Tartary buckwheat and *Arabidopsis* (Figures S12 and S13, Supporting Information). Moreover, the overexpression of *FtMGT2* enhanced kaempferol accumulation, while *mgt2‐C* hairy roots showed a significant decreased, suggesting *FtMGT2* could promotes the biosynthesis or accumulation of flavonoid metabolites like kaempferol.

To further reveal the mechanism of *FtMGT2* in plant response to salt stress, *FtMGT2* heterologous expression *Arabidopsis* lines were generated. The heterologous expression of *FtMGT2* invoked no significant root phenotype change compared to wild type (WT). However, following treatment with 100 × 10^−3^ m NaCl, both root length and biomass were significantly higher in the *FtMGT2* heterologous expression lines than in the WT (Figure 2D,E and Figure S14, Supporting Information), indicating that the overexpression of *FtMGT2* in *Arabidopsis* improves overall growth performance under salt stress. Furthermore, the kaempferol content in the *Arabidopsis* heterologous expression lines was also elevated, consistent with the findings in FtMGT2 hairy roots (Figure S15, Supporting Information).

We then carried out a standard salt response assay of *FtMGT2* overexpressing *Arabidopsis*. These findings revealed that following salt treatment for two weeks, both *Arabidopsis* heterologous overexpression lines of *FtMGT2* and the WT experienced significant growth inhibition, characterized by reduced plant growth, yellowing of leaves and stems, and a notable decline in fruiting efficiency. However, the *Arabidopsis* heterologous expression lines retained more green regions and displayed partially normal flowering in contrast to WT plants. Moreover, two weeks post‐rehydration, *Arabidopsis* heterologous expression lines had restored vitality, exhibiting enhanced flowering and fruiting, while the majority of WT plants failed to recover or achieve a higher survival rate (Figure S16, Supporting Information). These outcomes demonstrate that *FtMGT2* overexpressing *Arabidopsis* lines may exhibit improved tolerance to salt stress. To further explore the regulatory role of *FtMGT2* in *Arabidopsis* salt tolerance, we next introduced *FtMGT2* into the *mgt2* mutant to create complementation lines and conducted salt tolerance assays on these materials. The findings revealed that *mgt2* exhibited heightened sensitivity to salt stress, showing substantial inhibition under salt treatment. In contrast, the complementation lines notably boosted the *mgt2* mutant's ability to withstand salt stress (Figure S17, Supporting Information). These results imply that *FtMGT2* plays a direct role in regulating *Arabidopsis*’ defense mechanism against salt stress.

To investigate the role of FtMGT2 in regulating ion flux, we measured the Na⁺ flux in *FtMGT2* overexpressing *Arabidopsis*. The results indicated that, under both normal and salt treatment conditions, the Na⁺ efflux in *FtMGT2* overexpressing *Arabidopsis* was higher than in the WT (Figure 2F), suggesting that *FtMGT2* may enhance salt tolerance in *Arabidopsis*. Similarly, we then treated the *FtMGT2* transgenic *Arabidopsis* with ultrapure water containing either 100 mM NaCl or a combination of 100 × 10^−3^ m NaCl and 50 × 10^−3^ m MgSO_4_. Subsequently, we measured the Na^+^ flux rates employing NMT. The results indicated that Na^+^ efflux in the *FtMGT2* overexpression materials significantly increased only in the presence of both Na^+^ and Mg^2+^ (Figure S18, Supporting Information), further demonstrating that *FtMGT2* influences Na^+^ transport exclusively in the presence of Mg^2+^. Furthermore, to investigate the downstream regulatory network, *FtMGT2* overexpressing *Arabidopsis* plants were subjected to differential expression analysis. It was found that the overexpression of *FtMGT2* could alter numerous genes involved in signaling of hormones associated with plant development and stress responses (Figure 2G). For instance, abscisic acid receptor PYLs,^[^ ^34^ ^]^ 1‐aminocyclopropane‐1‐carboxylate synthase,^[^ ^35^ ^]^ ethylene‐responsive transcription factors,^[^ ^36^ ^]^ gibberellin 2‐beta‐dioxygenase,^[^ ^37^ ^]^ and IAA‐amino acid hydrolase ILR1‐like genes^[^ ^38^ ^]^ were significantly increased in *FtMGT2* overexpressing lines. Moreover, the negative regulators of the signaling of jasmonic acid (JA) which is an important hormone promoting Tartary buckwheat resistance to *R. solani*, such as TIFY^[^ ^39^ ^]^ and jasmonate ZIM‐domain proteins,^[^ ^40^ ^]^ were also significantly increased. The varied expression of these genes in *FtMGT2* over‐expressing lines might be associated with the function of *FtMGT2* in plant development and stress response.

### 2.3 FtMGT2 Can Influence Plant Salt Tolerance by Transporting Mg^2+^

**Figure 3:**
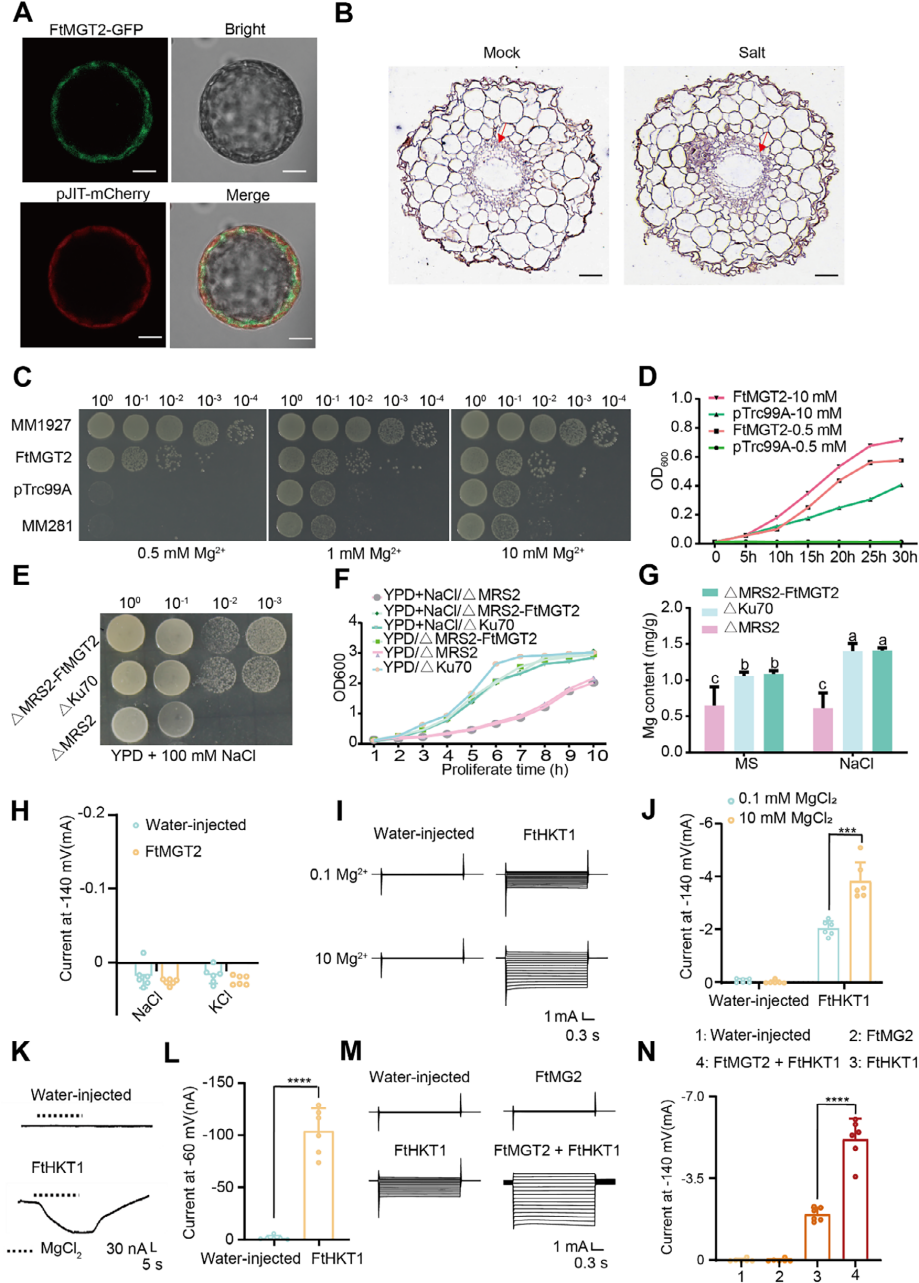
Validation of FtMGT2 transporter activity in yeast, *S. typhimurium* MM281 and *Xenopus oocyte* system. A) Subcellular localization of FtMGT2. FtMGT2‐GFP, and pCAMBIA1300‐FtMGT2 recombinant plasmid; pJIT‐mCherry, membrane marker; Bright, bright channel; Merge, merge channel. Bar = 10 µm. B) FISH assay of *FtMGT2* in the root. Mock: Control treatment, Salt: Salt treatment, the expression site of *FtMGT2* in the tan area. The blue‐purple signal indicates positive hybridization. Bar = 50 µm. C,D) Complementation of MM281 Mutant by *FtMGT2*. C) Growth of different bacterial cells on the LB medium containing 500 × 10^−6^ m, 1 × 10^−3^ m, 10 × 10^−3^ m MgSO_4_. The cells MM1927 as a positive control, MM281 transformed with *FtMGT2* cDNA in pTrc99A vector and MM281 transformed with pTrc99A vector only as a negative control. D) The bacterial cells shown in (C) were cultured in the LB medium containing different concentrations of MgSO_4_. E) The growth of three yeast strains was observed on YPD solid medium + 100 × 10^−3^ m Mg^2+^ + 100 × 10^−3^ m NaCl. F) The growth curves of three yeast strains in YPD liquid medium and YPD liquid medium + 100 × 10^−3^ m NaCl. G) The Mg^2+^ content of three yeast strains in YPD liquid medium and YPD liquid medium + 100 × 10^−3^ m Mg^2+^ + 100 × 10^−3^ m NaCl. Data are presented as the mean ± SD from *n* = 3 independent biological replicates. Statistical analysis was performed using one‐way ANOVA analysis with Tukey's HSD test (Different letters represent significant differences at *P* < 0.05). H–N) TEVC experiments were performed using *X. laevis oocytes* injected with water, *FtMGT2* cRNA or *FtHKT1* cRNA in the presence of different concentrations of Mg^2+^. H) Current–voltage relationships of oocytes injected with water or *FtMGT2* cRNA in the presence of 20 × 10^−3^ m NaCl or 20 × 10^−3^ m KCl. I) A current profile of water‐injected or FtHKT1 oocytes, bathed in different concentrations of Mg^2+^. J) Current–voltage relationships of oocytes injected with water or *FtHKT1* cRNA in different concentrations of Mg^2+^. K) A current profile of water‐injected or *FtHKT1* oocytes, bathed in different concentrations of Mg^2+^. L) Current–voltage relationships of oocytes injected with water or *FtHKT1* cRNA in the presence of Mg^2+^. M) A current profile of water‐injected, *FtMGT2*, *FtHKT1*, or *FtMGT2* + *FtHKT1* oocytes, bathed in the presence of Na^+^. N) Current–voltage relationships of oocytes injected with water‐injected, *FtMGT2*, *FtHKT1*, or *FtMGT2* + *FtHKT1* oocytes, bathed in the presence of Na^+^. Data in (H), (J), (L), and (N) are presented as the mean ± SD from *n* = 3 independent biological replicates. Statistical significance was determined using a two‐sided Student's *t*‐test. Asterisks indicate significant differences (***P < 0.001; ****P < 0.0001).

To further reveal the mechanism by which *FtMGT2* participates in the Tartary buckwheat response to salt stress, the subcellular localization of FtMGT2 was identified using transient expression in *Nicotiana benthamiana* protoplasts. It is found that FtMGT2 was localized on the cell membrane (**Figure** **3**A), which is in accordance with the subcellular localization of MGT1.^[^ ^41^ ^]^ To further investigate the expression of *FtMGT2* in Tartary buckwheat, we performed quantitative real‐time polymerase chain reaction on different Tartary buckwheat tissues and found that *FtMGT2* is most highly expressed in the roots (Figure S19, Supporting Information). By contrast, the expression of *FtMGT2* in the stem and flowers is low, a fact that we ascribe to its likely function in transporting substances from the roots to the leaves rather than residing in the stem. To validate this hypothesis, we generated the pCAMBIA1391‐*FtMGT2* promoter‐GUS and transformed it into *Arabidopsis*. Subsequent, GUS staining of the transgenic *Arabidopsis* lines exhibited notable GUS expression in the roots and mature leaves, which was consistent with our initial hypothesis (Figure S20, Supporting Information).

To test the functional significance of the A/G polymorphism, we performed site‐directed mutagenesis on the *FtMGT2* promoter from genotype A. Specifically, the “A” allele was mutated to a “G” (mutant A), and this modified promoter was fused to a GUS reporter gene and transformed into *Arabidopsis*. GUS staining revealed that the activity of the mutant A promoter was significantly lower than that of the native genotype A promoter (Figure S21A,B,E, Supporting Information). Conversely, we performed the reciprocal experiment, mutating the “G” allele in the genotype G promoter to an “A” (mutant G), and transformed this construct into *Arabidopsis*. As expected, GUS activity driven by the mutant G promoter was significantly higher than that of the native genotype G promoter (Figure S21C,D–E, Supporting Information). These reciprocal mutagenesis experiments indicate that the “A” allele in the *FtMGT2* promoter is sufficient to enhance gene expression in both roots and leaves. To determine the tissue‐specific expression pattern of *FtMGT2*, we performed fluorescence in situ hybridization (FISH) on Tartary buckwheat seedling roots. *FtMGT2* transcripts were highly abundant in the vascular and epidermal cells of the root tip, and their abundance increased following salt stress (Figure 3B). Collectively, these results collectively indicate that *FtMGT2* is probably involved in Tartary buckwheat response to salt stress.

To determine if *FtMGT2* encodes a functional Mg^2^⁺ transporter, we performed a complementation assay using the *Salmonella typhimurium* mutant strain MM281. This strain lacks its native Mg^2^⁺ transport systems and requires high concentrations of supplemental magnesium (10 × 10^−3^ m Mg^2^⁺) for growth in basal medium. On medium with a low, growth‐restrictive magnesium concentration (0.5 × 10^−3^ m Mg^2^⁺), both the positive control strain (MM1927) and the MM281 mutant expressing *FtMGT2* were able to grow. In contrast, the negative controls—the untransformed MM281 mutant and MM281 transformed with an empty vector (pTrc99A)—failed to grow (Figure 3C). This finding was further corroborated by growth curve analysis in liquid culture (Figure 3D). Collectively, these results demonstrate that heterologous expression of *FtMGT2* rescues the Mg^2^⁺ transport deficiency of the *S. typhimurium* MM281 mutant, confirming that *FtMGT2* is a functional magnesium transporter.

To further reveal the function of *FtMGT2*, the *△Ku70* yeast system^[^ ^42^ ^]^ (A yeast mutant strain of *Pichia pastoris* was regarded as capable of enhancing the efficiency of CRISPR system‐mediated gene knockout and integration) was used to examine whether FtMGT2 functions as a transporter. To this end, we generated a yeast mutant line, *△MRS2*, by deleting its endogenous homolog, *MRS2*, using CRISPR/Cas9‐mediated gene editing (Figure S22A, Supporting Information). Subsequently, the FtMGT2 gene was transformed into the △MRS2 mutant to generate the complemented line, *△MRS2‐FtMGT2*. On YPD medium supplemented with a high concentration of magnesium (100 × 10^−3^ m Mg^2^⁺), the *△MRS2* mutant exhibited a severe growth defect compared to the wild‐type control (*△Ku70*). This growth defect was significantly rescued by the expression of *FtMGT2* in the complemented strain (*△MRS2*‐*FtMGT2*). These results confirm that FtMGT2 is a functional magnesium transporter capable of restoring tolerance to high‐magnesium conditions in yeast (Figure S22B, Supporting Information). Sequence analysis revealed that the N‐terminal 377‐441 amino acids constituted the predicted transmembrane domain of FtMGT2. We then removed the predicted transmembrane domain of FtMGT2, forming a mutant without the transmembrane domain (*Ftmgt2*; Figure S22C, Supporting Information), and subsequently transformed the mutated *Ftmgt2* into the *△MRS2* mutant yeast, forming the partial complementation strain, *△MRS2‐Ftmgt2*. The growth rate of the partial complementation strain was significantly lower than that of the *△Ku70* wild‐type yeast and the fully complemented strain, *△MRS2‐FtMGT2* (Figure S22D,E, Supporting Information). These findings suggested that FtMGT2 is crucial for magnesium transport, and that the transmembrane structure is indispensable for FtMGT2 function as a magnesium transporter.

To investigate the role of *FtMGT2* in salt stress, we analyzed the growth rate after yeast cells treated with NaCl. We found the growth rate of the *△MRS2* mutant yeast was significantly lower than that of the *△Ku70* wild‐type yeast (Figure 3E,F). However, the growth rate of the complemented strain (*△MRS2‐FtMGT2*) was significantly higher than that of the mutant yeast (*△MRS2*) and comparable to the wild type (*△Ku70*), suggesting that *FtMGT2* was involved in resistance to salt stress. Additionally, a separate treatment with NaCl on different yeast strains revealed that the growth rate of the *FtMGT2* yeast strain did not change significantly, indicating that *FtMGT2* is unable to transport Na^+^ within the yeast system (Figure s22 D,E, Supporting Information). Additionally, Mg^2+^ content analysis revealed that *△MRS2* contained the lowest levels of Mg^2+^, whereas the complemented strain *△MRS2‐FtMGT2*2 displayed the highest levels following salt stress (Figure 3G). These findings suggest that *FtMGT2* is directly involved in magnesium transport in yeast and modulates the yeast's reaction to salt stress.

HKT1 is a key transporter protein in plants responsible for the movement of Na⁺ and K⁺.^[^ ^22^ ^]^ To determine whether FtMGT2 affects the activity of FtHKT1, we conducted two‐electrode voltage clamp (TEVC) experiments. Previous studies have shown that Mg^2+^ currents are difficult to detect using TEVC.^[^ ^43^ ^]^ Therefore, we then employed TEVC to determine whether Na^+^ and K^+^ currents could be detected in the membranes of *Xenopus oocytes* injected with *FtMGT2*. The results indicated that the currents in the membranes of oocytes injected with *FtMGT2* were unchanged compared to the water controls, suggesting that FtMGT2 does not possess the ability to transport Na^+^ and K^+^ (Figure 3H). However, FtHKT1 exhibited strong Na⁺ transport activity. Interestingly, the addition of exogenous Mg^2^⁺ significantly enhanced the Na⁺ activity of FtHKT1 (Figure 3I–L), while the addition of exogenous Ca^2^⁺ did not influence its activity (Figure S23, Supporting Information). Notably, when *FtMGT2* and *FtHKT1* were co‐expressed, the activity of FtHKT1 significantly increased (Figure 3M,N). Together, these results demonstrate that Mg^2^⁺ can enhance FtHKT1 activity, which indicates that *FtMGT2* may participate in the mechanism by which Tartary buckwheat mitigates salt stress through the regulation of Na⁺/K⁺ transporters.

To investigate the genetic relationship between *FtMGT2* and *FtHKT1* in plant, we generated several *hkt1* mutant *Arabidopsis* lines and hairy roots (Figure S24A, Supporting Information). After germinating and culturing these different *Arabidopsis* lines in ultrapure water, we then treated them with Na^+^, Mg^2+^ or a combination of Na^+^ and Mg^2+^, and subsequently measured the Na^+^ and Mg^2+^ flux rates using NMT (Figure S24B,C, Supporting Information). The results indicated that Na^+^ efflux was significantly decreased in the *hkt1* mutant, *hkt1*/ *mgt2* double mutant, and *hkt1*/ *FtMGT2* complementation line and remained consistent under all three treatment conditions. In contrast, the *hkt1* / *FtHKT1* complementation line exhibited significantly enhanced Na^+^ efflux when treated with Na^+^, and the strongest Na^+^ efflux occurred under the combined Na^+^ and Mg^2+^ treatment. Similarly, we conducted experiments on hairy roots to investigate the effects of mutations and overexpression of *FtHKT1* under different ion treatments (Figure S24D,E, Supporting Information). On the other hand, the measurement results of Mg^2+^ flux suggest that differences in Mg^2+^ flux rates in plants under various treatments only occur in the presence of FtMGT2. This indicates that FtMGT2 may act upstream of FtHKT1. The results indicated that Na^+^ efflux in the *hkt1‐C* hairy roots significantly decreased after treatment, while Na^+^ efflux in the *FtHKT1* overexpression hairy roots increased, with the highest efflux observed when both Na^+^ and Mg^2+^ were present, suggesting that FtHKT1 is a key transporter for Na^+^ and its Na^+^ transport activity can be regulated by Mg^2+^.

### 2.4 The FtAGL16‐FtMYB15L Complex Directly Regulates *FtMGT2* Transcription

**Figure 4:**
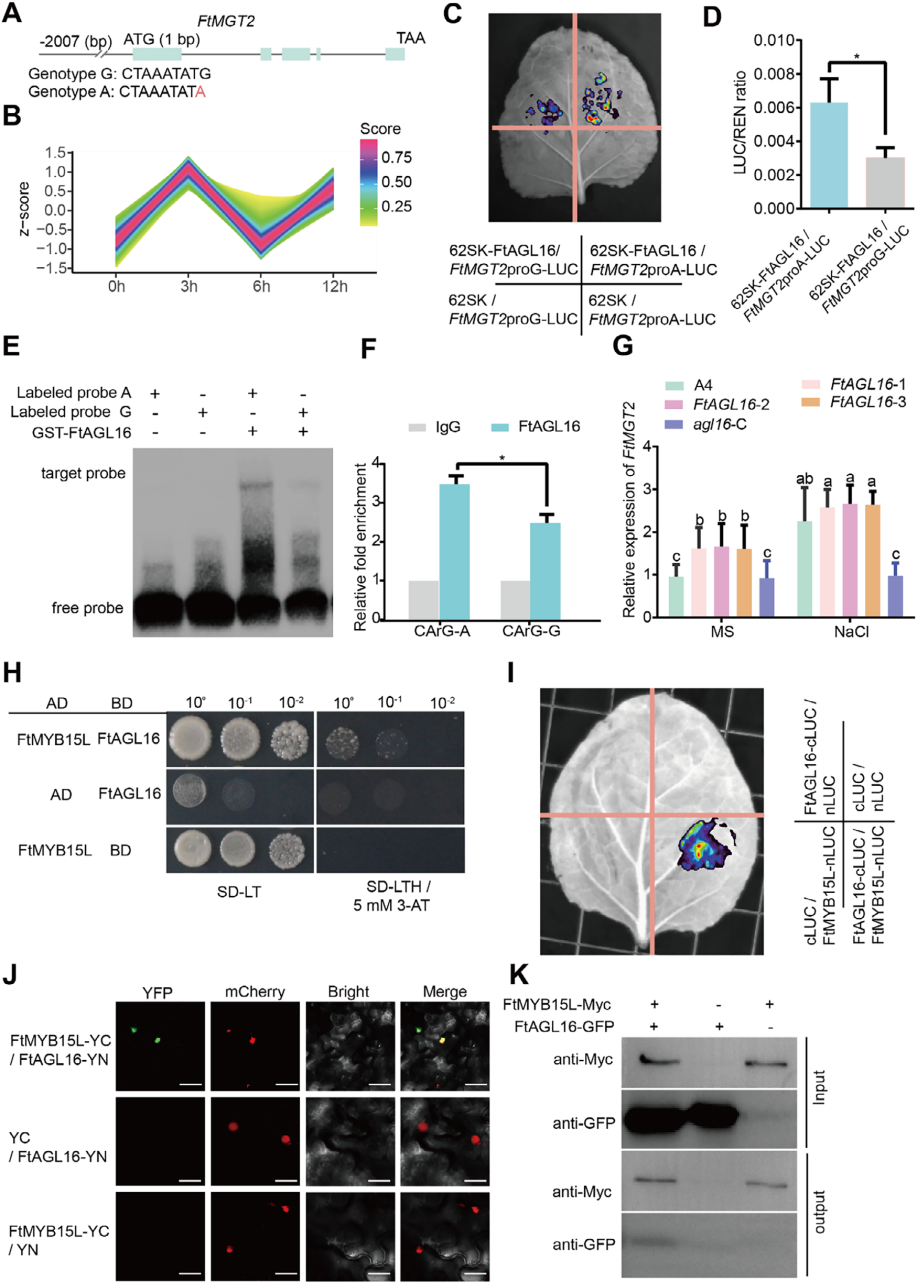
The transcription factor FtAGL16 directly bind to *FtMGT2* promoter and interact with FtMYB15L. A) The gene structure of the *FtMGT2* genomic sequence and its corresponding box diagram illustrate the coding region. B) Cluster that is co‐expressed with *FtMGT2* has been identified in the salt transcriptome. C) Using the LUC system to detect the LUC fluorescence imaging of 62SK‐FtAGL16 and 62SK in combination with *FtMGT2* promoter mini‐LUC. D) LUC/REN ratios of various combinations were analyzed under condition C. E) EMSA experimental results of GST‐FtAGL16 protein and *FtMGT2* promoter probe. Free probe, unbound probe, target probes, and probes that specifically bind to the FtAGL16, probe A: genotype A sequence of the *FtMGT2* promoter, probe G: genotype G sequence of the *FtMGT2* promoter. F) The ChIP‐qPCR result shows the FtAGL16 can specifically bind to *FtMGT2* promoter sequence using fold enrichment method. G) The expression of *FtMGT2* in *FtAGL16* overexpression and knockout hairy roots under normal and salt stress conditions. Data are presented as the mean ± SD from *n* = 3 independent biological replicates. Statistical analysis was performed using one‐way ANOVA analysis with Tukey's HSD test (Different letters represent significant differences at *P* < 0.05). H) Y2H results between FtAGL16 and FtMYB15L, SD‐LT, SD basal medium lacked the Leu and Trp; SD‐LTH / 5 × 10^−3^ m 3‐AT, SD basal medium lacked Leu, Trp, and His, containing 5 × 10^−3^ m 3‐AT. I) LCI result between FtAGL16 and FtMYB15L, the fluorescence indicates that LUC is activated. J) BiFC assay showing interactions between FtAGL16 and FtMYB15L in *N. benthamiana* leaf epidermal cells. FtAGL16 was fused to the N‐terminal fragment of YFP (YN), and FtMYB15L was fused to the C‐terminal fragment of YFP (YC), GFP, GFP channel; mCherry, H2B‐nuclear marker; Bright, bright channel; Merge, merge channel. Bar = 20 µm. K) Co‐IP assay showing the interactions between FtAGL16 and FtMYB15L in vivo. FtMYB15L‐Myc protein were extracted by *FtMYB15L* overexpressing hairy roots and incubated with FtAGL16‐GFP protein using Myc beads. FtMYB15L‐Myc proteins in the immunoprecipitated fraction were detected by immunoblotting with an anti‐GFP antibody. Data in (D) and (F) are presented as the mean ± SD from *n* = 3 independent biological replicates. Statistical significance was determined using a two‐sided Student's *t*‐test. Asterisks indicate significant differences (*P < 0.05).

To investigate the mechanism of the varied *FtMGT2* expression in accessions of different genotype, the SNP on the promoter of *FtMGT2* was further analyzed. Sequence analysis revealed that the G to A transition alters a CArG motif (**Figure** **4**A), one of the MADS transcription factor binding sites in genotype A, suggesting MADS transcription factor might directly bind to the promoter of *FtMGT2*. Therefore, to identify upstream MADS transcription factors, we analyzed genes co‐expressed with *FtMGT2* in our salt‐stress transcriptome data. A total of 763 genes were found to be co‐expressed with *FtMGT2* (Figure 4B and Table S6, Supporting Information, in express_cluster 9, only have one MADS protein). Among them, a MADS transcription factors family gene (*FtPinG0403785000*, named *FtAGL16*) was found to be co‐expressed with *FtMGT2*. To investigate whether FtAGL16 could regulate *FtMGT2* expression, we performed a dual‐luciferase (LUC) reporter assay, the results showed that FtAGL16 activated the *FtMGT2* promoter, with the promoter sequence from genotype A driving significantly higher LUC activity than that from genotype G (Figure 4C,D). To test for direct physical interaction, we conducted an electrophoretic mobility shift assay (EMSA). Consistent with the LUC assay results, the EMSA demonstrated that the FtAGL16 protein binds directly to the *FtMGT2* promoter and exhibited a significantly higher binding affinity for the genotype A sequence compared to the genotype G sequence. (Figure 4E). We then performed chromatin immunoprecipitation (ChIP)‐qPCR on *FtAGL16* OE hairy roots and found that the its can specifically bind to *FtMGT2* promoter sequence (Figure 4F). Collectively, these findings demonstrate that FtAGL16 can directly bind to the *FtMGT2* promoter and that this binding is enhanced by the “A” allele, leading to activated *FtMGT2* expression.

To examine if *FtAGL16* directly impacts Tartary buckwheat's response to salt stress, we overexpressed and knocked out *FtAGL16* in Tartary buckwheat hairy roots. After obtaining the overexpressing and knockout lines (Figure S25A–C, Supporting Information), we then examined the expression of *FtMGT2* and *FtAGL16*. However, *FtAGL16* overexpression or knockout did not result in a significant change in FtMGT2 expression levels (Figure 4G and Figure S25D, Supporting Information). Subsequently, we simultaneously knocked out *FtAGL16* and *FtMGT2* in Tartary buckwheat hairy roots. Under salt stress conditions, the *agl16‐C* / *mgt2‐C* double mutant exhibited elevated stress levels compared to the *mgt2‐C* mutant, although this difference was not statistically significant (Figure 2A–C and Figure S25E). This finding suggests that FtAGL16 plays a partial, rather than a sole, regulatory role, implying that other transcription factors likely act in concert with FtAGL16 to modulate *FtMGT2* expression.

To investigate whether other transcription factors are involved with *FtAGL16* in regulating *FtMGT2* expression, we analyzed genes co‐expressed with both *FtMGT2* and *FtAGL16* in transcriptome response to salt stress, and 14 transcription factors were found (Table S8, Supporting Information, in express_cluster 9). Previous studies have shown that MADS proteins can interact with MYB proteins to co‐regulate the expression of downstream genes.^[^ ^44^ ^]^ Thus, we focus on MYB transcription factors in this list and identified a gene encoding FtMYB15L transcription factor (*FtPinG0606538500*) co‐expressed with *FtMGT2* and *FtAGL16*. To investigate whether FtMYB15L interacts with FtAGL16, we first conducted yeast two‐hybrid (Y2H) experiments, the results showed that FtMYB15L and FtAGL16 successfully cultured yeast colonies in the SD‐LTH medium with 20 mm 3‐AT. However, yeast growth occurred normally only when both FtMYB15L and FtAGL16 were simultaneously present, suggesting a direct interaction between FtMYB15L and FtAGL16 (Figure 4H). Furthermore, firefly luciferase fragment complementary imaging technology (LCI) assay results confirmed this interaction, showing that FtMYB15L‐cLUC can interact with FtAGL16‐nLUC to activate the LUC fluorescent signaling (Figure 4I). Subsequently, bimolecular fluorescence complementation assay (BiFC) was also verified the interaction of FtMYB15L and FtAGL16, as a YFP signal was observed when FtMYB15L and FtAGL16 were co‐expressed (Figure 4J) We then conducted co‐immunoprecipitation (Co‐IP) experiments on FtMYB15L OE hairy roots, with the results illustrating that FtMYB15L‐Myc can specifically bind to FtAGL16‐GFP, indicating that FtMYB15L and FtAGL16 can interact in vivo (Figure 4K). Collectively, these findings provide robust evidence that FtMYB15L has the capability to interact with FtAGL16.

**Figure 5:**
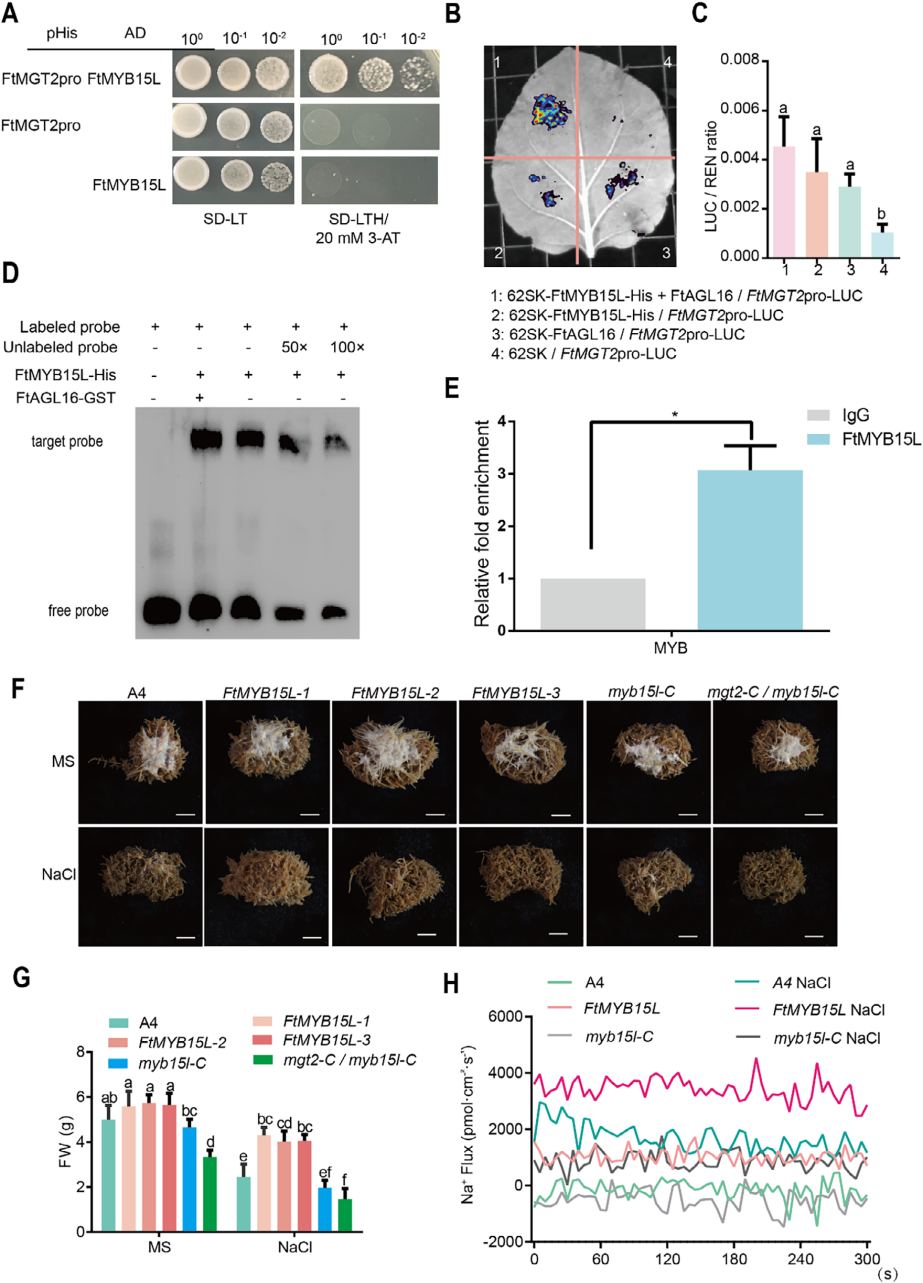
The transcription factor FtMYB15L exerts a positive regulatory effect on *FtMGT2*. A) The Y1H results of *FtMGT2* promoter and FtMYB15L. SD‐LT, SD basal medium lacked the Leu and Trp; SD‐LTH / 20 × 10^−3^ m 3‐AT, SD basal medium lacked Leu, Trp, and His, containing 20 × 10^−3^ m 3‐AT. B) Using the LUC system to detect the LUC fluorescence imaging of 62SK‐FtMYB15L and 62SK in combination with *FtMGT2* promoter mini‐LUC. C) LUC/REN ratios of various combinations were analyzed under condition B. D) EMSA experimental results of His‐FtMYB15L protein and FtAGL16‐GST with *FtMGT2* promoter probe. Free probe, unbound probe, target probes, probes that specifically bind to the FtMYB15L protein. E) The ChIP‐qPCR result shows the FtMYB15L can specifically bind to *FtMGT2* promoter sequence using fold enrichment method. Data are presented as the mean ± SD from *n* = 3 independent biological replicates. Statistical significance was determined using a two‐sided Student's *t*‐test. Asterisks indicate significant differences (*P < 0.05). F) Phenotype of *FtMYB15L* overexpressed and knockout hairy roots exposed to salt stress conditions. A4, A4 *Agrobacterium rhizogenes* empty strain hairy roots; *FtMYB15L‐1*, *FtMYB15L‐2*, and *FtMYB15L‐3*, three *FtMYB15L* overexpressed hairy root strains, *myb15‐C*, *FtMYB15L* knockout hairy roots; *mgt2‐C/myb15l‐C*, double mutant hairy roots of *FtMGT2* and *FtMYB15L*. Bar = 5 mm. G) The fresh weight was observed in A4 hairy roots, *FtMYB15L* overexpressed and knockout hairy roots both under normal conditions (MS) and after exposure to 100 × 10^−3^ m NaCl salt stress for two weeks (NaCl). H) Na^+^ fluxes measured from the root of different hairy root. Data in (C) and (G) are presented as the mean ± SD from *n* = 3 independent biological replicates. Statistical analysis was performed using one‐way ANOVA analysis with Tukey's HSD test (Different letters represent significant differences at *P* < 0.05).

To investigate whether *FtMYB15L* is also involved in regulating *FtMGT2* expression in the Tartary buckwheat response to salt stress, yeast one hybridization (Y1H) validation experiments were performed. The results of these studies revealed that FtMYB15L could bind to the promoter of *FtMGT2* (**Figure** **5**A). The effect of FtMYB15L on *FtMGT2* was then analyzed by transient transcriptional activity assays. These assays illustrated that *FtMYB15L* could increase the expression of LUC, and when both FtAGL16 and FtMYB15L are present simultaneously, the fluorescence signal of LUC is stronger (Figure 5B,C). The promoter sequence of *FtMGT2* was further analyzed, and a MYB binding motif TTTGGT was found (Figure S26, Supporting Information). EMSA analysis revealed that FtMYB15L could directly bind to the motif on the promoter of *FtMGT2* (Figure 5D), and when FtMYB15L and FtAGL16 coexist, the probe's binding capability is stronger, indicating that FtMYB15L and FtAGL16 could cooperatively strongly regulate the expression of *FtMGT2*. We then performed ChIP‐qPCR using *FtMYB15L* OE hairy roots and found that FtMYB15L can specifically bind to the *FtMGT2* promoter sequence, demonstrating that FtMYB15L can directly bind to the *FtMGT2* promoter in vivo. Furthermore, when FtAGL16 and FtMYB15L proteins are present simultaneously, their binding ability to the *FtMGT2* promoter sequence is stronger (Figure 5E). Collectively, these findings demonstrate that FtMYB15L is a transcription activator that directly targets FtMGT2, that FtAGL16 and FtMYB15L act synergistically to co‐regulate and enhance the expression of *FtMGT2*.

To further investigate the role of *FtMYB15L* in the salt response of Tartary buckwheat, we then conduct the subcellular localization of FtMYB15L. The results revealed that FtMYB15L was localized in the nucleus (Figure S27, Supporting Information). To investigate the function of FtMYB15L, we generated transgenic Tartary buckwheat hairy roots overexpressing (*FtMYB15L*‐OE) or with knockout of *FtMYB15L* (*myb15l‐C*) (Figure S28A–C, Supporting Information). The *FtMYB15L*‐OE lines exhibited enhanced salt tolerance compared to the control roots (Figure 5F–H). Conversely, the *myb15l‐C* knockout lines displayed a salt‐sensitive phenotype. In these *myb15l‐C* mutant lines, *FtMYB15L* expression was barely detectable, and the transcript levels of *FtMGT2* were also significantly reduced (Figure S28D,E, Supporting Information). Furthermore, the *myb15l‐C* single mutant also showed reduced kaempferol and Mg^2^⁺ contents, a phenotype that mirrored the *mgt2‐C* mutant (Figure S29A,B, Supporting Information). To further probe the genetic interaction between these genes, we generated a *myb15l‐C*/ *mgt2‐C* double knockout mutant (Figure S28A–C, Supporting Information). Under salt stress, the *myb15l‐C*/*mgt2‐C* double mutant exhibited a more severe salt‐sensitive phenotype than either the *myb15l‐C* or *mgt2‐C* single mutants (Figure 5F–H; Figures S28D,E and S29A,B, Supporting Information). In addition, *FtMYB15L* overexpressing *Arabidopsis* exhibited the highest Na⁺ efflux in the roots under salt stress (Figure 5H). These findings highlight the essential roles of *FtMYB15L* and *FtMGT2* as key genes in conferring salt stress resistance in Tartary buckwheat. We next constructed *FtMYB15L* overexpression *Arabidopsis* lines. The *FtMYB15L* overexpression lines exhibited higher salt tolerance than WT (Figure S30, Supporting Information), which in consistent with *FtMYB15L* increasing *FtMGT2* expression. In addition, the *FtMYB15L* overexpression lines exhibited higher amounts of kaempferol than those found in the WT following NaCl treatment, which is in accordance with the results of *FtMGT2* accumulating kaempferol. We then conducted standard salt treatment experiments on *FtMYB15L* OE *Arabidopsis*. The results showed that the *FtMYB15L* OE *Arabidopsis* exhibited a phenotype similar to *FtMGT2* OE *Arabidopsis* after salt treatment, retaining more green tissues compared to the WT and displaying normal flowering and fruiting after watering (Figure S31, Supporting Information). These results demonstrated that *FtMYB15L* increased the expression of *FtMGT2*, thus enhancing plant resistant to salt stress.

### 2.5 The E3 Ubiquitin Ligase FtBRG1 Assembles with the *FtMGT2* Transcriptional Activator FtMYB15L

**Figure 6:**
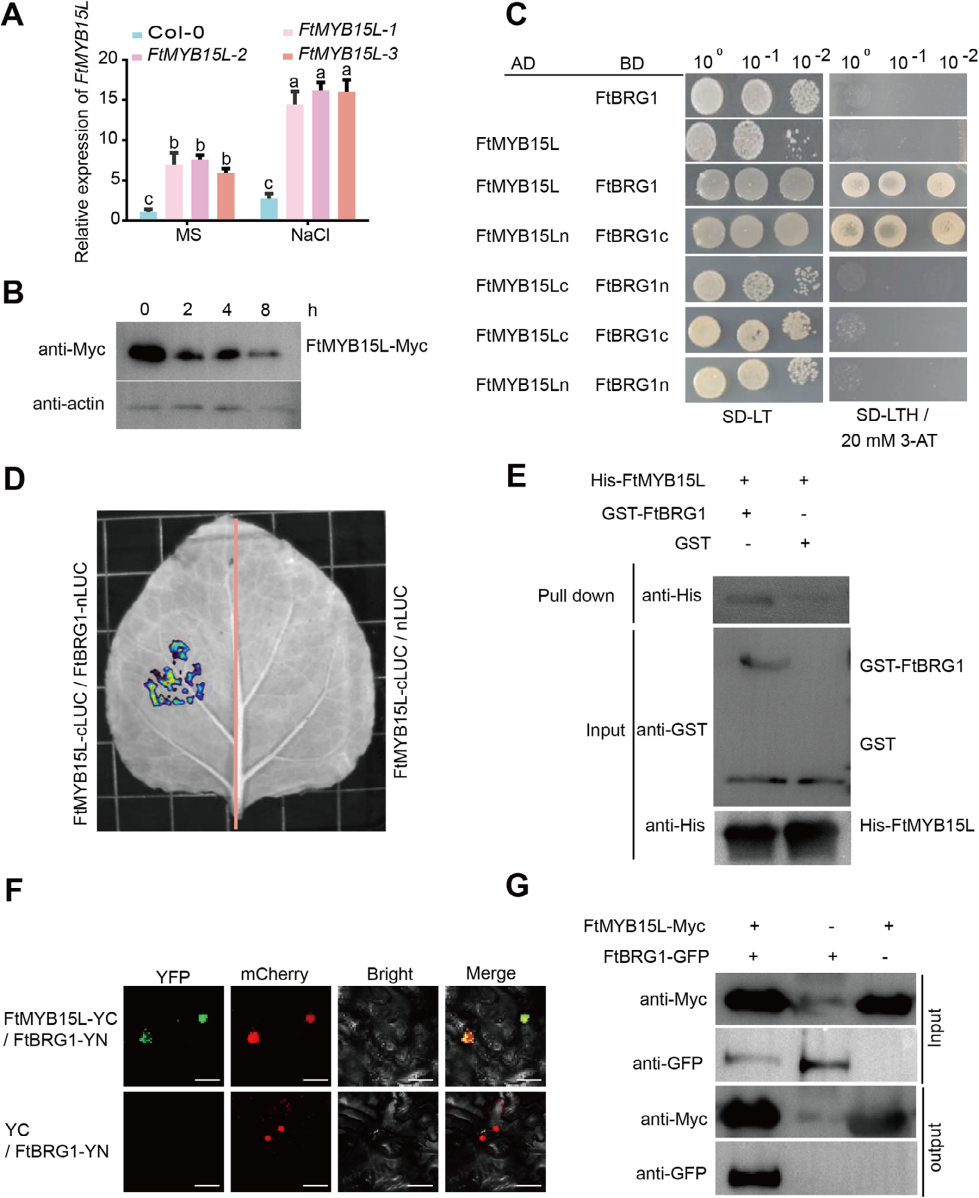
The investigation of the interaction between FtBRG1 and FtMYB15L. A) The *FtMYB15L* expression of Col‐0 and *FtMYB15L* overexpressed *Arabidopsis* under normal conditions and under 3 h salt stress conditions. Data are presented as the mean ± SD from *n* = 3 independent biological replicates. Statistical analysis was performed using one‐way ANOVA analysis with Tukey's HSD test (Different letters represent significant differences at *P* < 0.05). B) The protein accumulation of *FtMYB15L* overexpressed *Arabidopsis* under normal conditions and at different times of salt treatments, anti‐actin, actin anti‐mouse antibody, anti‐Myc, Myc anti‐mouse antibody. C) Y2H results of FtMYB15L and FtBRG1. FtMYB15L and FtMYB15L full length; FtMYB15Lc and FtMYB15L C domain truncation; FtMYB15Ln and FtMYB15L N domain truncation; FtBRG1 and FtBRG1 full length; FtBRG1c and FtBRG1 C domain truncation; FtBRG1n and FtBRG1 N domain truncation. SD‐LT, the SD basal medium lacked Leu and Trp; SD‐LTH/20 × 10^−3^ m 3‐AT, SD basal medium lacked Leu, Trp, and His, containing 20 × 10^−3^ m 3‐AT. D) Fluorescence imaging results determination after injecting FtMYB15L‐cLUC with FtBRG1‐nLUC and p2300‐nLUC into *N. benthamiana* leaves, respectively. E) Pull‐down results for His‐FtMYB15L and GST‐FtBRG1. Input, protein combination without beads; pull‐down, protein combination with GST beads; +, indicates the presence of this protein in the combination. –, indicates the absence of this protein in the combination. anti‐His, His anti‐mouse antibody. anti‐GST, GST anti‐mouse antibody. F) BiFC assay showing interactions between FtBRG1 and FtMYB15L in *N. benthamiana* leaf epidermal cells. FtBRG1 was fused to the N‐terminal fragment of YFP (YN), and FtMYB15L was fused to the C‐terminal fragment of YFP (YC), GFP, GFP channel; H2B‐mCherry, nuclear marker; Bright, bright channel; Merge, merge channel. Bar = 20 µm. G) Co‐IP assay showing the interactions between FtBRG1 and FtMYB15L in vivo, FtMYB15L‐Myc protein were extracted by FtMYB15L overexpressing hairy roots and incubated with FtBRG1‐GFP protein using Myc beads. FtMYB15L‐Myc proteins in the immunoprecipitated fraction were detected by immunoblotting with an anti‐GFP antibody.

To further reveal the function of *FtMYB15L* in plant response to salt stress, the transcription and protein levels of FtMYB15L were analyzed. The expression of *FtMYB15L* was significantly increased after 100 mM NaCl treatment in A4 hairy roots and *FtMYB15L* overexpression lines (**Figure** **6**A). Moreover, the protein level of FtMYB15L was significantly decreased after 100 mM NaCl treatment (Figure 6B). This inverse relationship strongly suggests that a protein degradation pathway may precisely regulate FtMYB15L at the protein level in plants. Yeast two hybrid analysis was then used to investigate whether E3 ubiquitin ligases in the co‐expression gene cluster of *FtMGT2* could interact with FtMYB15L (Figure S32 and Table S8, Supporting Information). A BOI‐related E3 ubiquitin‐protein ligase 1 (*FtBRG1*) gene was found to interact with FtMYB15L (Figure 6C), and the C‐terminal of FtBRG1 (172‐329 aa) and N‐terminal of FtMYB15L (1‐125 aa) were responsible for this interaction. Subcellular localization experiments indicated that FtBRG1 is also localized in the nucleus (Figure S33, Supporting Information). Luciferase complementation imaging and pull down experiments further confirmed FtBRG1 could interact with FtMYB15L (Figure 6D,E). Moreover, the results of BiFC assay identified FtMYB15L could interact with FtBRG1 (Figure 6F). We next conducted Co‐IP experiments on *FtMYB15L* OE hairy roots, and the results showed that FtMYB15L‐GFP can specifically pulled down FtBRG1‐Myc, indicating that FtMYB15L and FtBRG1 can interact in vivo (Figure 6G).

**Figure 7:**
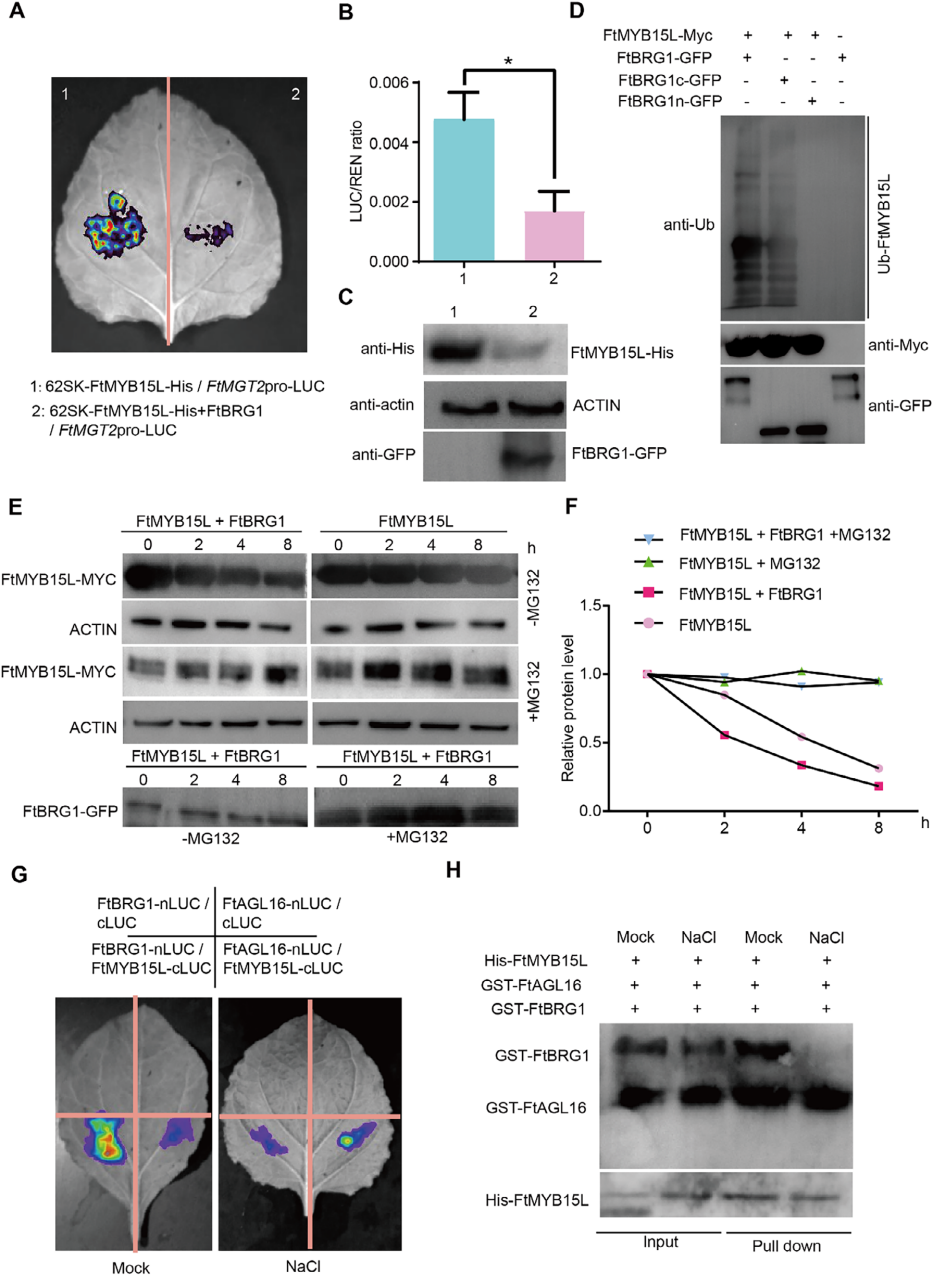
The FtBRG1 interacts with FtMYB15L and induces ubiquitination, thereby modulating the regulatory mechanisms of *FtMGT2*. A,B) *FtMGT2*pro‐miniLUC was co‐injected with 62SK‐FtMYB15L‐His and 62SK‐FtMYB15L‐His+FtBRG1 for LUC fluorescence. Data are presented as the mean ± SD from *n* = 3 independent biological replicates. Statistical significance was determined using a two‐sided Student's *t*‐test. Asterisks indicate significant differences (*P < 0.05). C) Immunoblotting results of FtMYB15L and FtBRG1 by transient transformed *N. benthamiana* leaves in (a), anti‐ub, ubiquitin antibody; anti‐Myc, anti Myc mouse antibody. anti‐GFP, anti GFP mouse antibody. D) Ubiquitination results of FtMYB15L and FtBRG1 by transient transformed *N. benthamiana* leaves, anti‐ub, Ubiquitin antibody; anti‐Myc, anti‐Myc mouse antibody. anti‐GFP, anti‐GFP mouse antibody. E,F) Protein degradation assay showing that FtMYB15L degradation is facilitated by FtBRG1. FtMYB15L‐Myc or both FtMYB15L‐Myc and FtBRG1 were transiently transformed *N. benthamiana* leaves. Plant total proteins were extracted from different seedlings. Protein extractions are incubated for the indicated time and used for immunoblotting assays. FtMYB15L‐Myc was detected by immunoblotting with an anti‐Myc antibody, FtBRG1‐GFP was detected by immunoblotting with an anti‐GFP antibody, and Actin was used as the internal control E, with the relative protein level of FtMYB15L‐Myc are shown in (F). G) Fluorescence imaging results between FtBRG1 and FtMYB15L or FtAGL16 and FtMYB15L under different conditions, Mock, under control condition, NaCl, under 100 × 10^−3^ m NaCl condition. H) Pull‐down result between FtBRG1 and FtMYB15L or FtAGL16 and FtMYB15L under different conditions, input, protein combination without beads; Pull‐down, protein combination with beads; +, indicates the presence of this protein in the combination. –, indicates the absence of this protein in the combination. anti‐His, His anti‐mouse antibody. anti‐GST, GST anti‐mouse antibody.

To further investigate how FtBRG1 assembled with FtMYB15L and the impact that this has on *FtMGT2* expression level, *FtBRG1* overexpressing and knockout Tartary buckwheat hairy roots were constructed (Figure S34A, Supporting Information) and tested their salt resistance. The results demonstrated that *FtBRG1* negatively regulates salt tolerance (Figure S34B–E, Supporting Information). The expression of *FtBRG1* was significantly higher while *FtMGT2* was significantly lower in *FtBRG1* overexpressing hairy roots compared to those in the control (Figure S34B, Supporting Information). Consistent with *FtMGT2* expression, the kaempferol contents was also significantly lower in *FtBRG1* overexpressing hairy roots. Conversely, the *brg1*‐C knockout lines exhibited the opposite phenotype: *FtBRG1* transcript was barely expressed while that of *FtMGT2* was significantly increased (Figure S34C, Supporting Information), and the kaempferol contents were significantly higher than those of control hairy roots (Figure S34D,E, Supporting Information). These results indicate that *FtBRG1* negatively regulates the *FtMGT2*‐mediated salt tolerance pathway. A transient transcriptional activity assay exhibited that FtBRG1 could alleviate the transcriptional activation effect of FtMYB15L on *FtMGT2* (**Figure** **7**A,B), which indicates that the FtMYB15L protein could be degraded following its co‐transformation with FtBRG1 (Figure 7C).

To further explore the impact of FtBRG1 on FtMYB15L protein degradation, we subjected the proteins to ubiquitination assays. The findings revealed that FtMYB15L undergoes ubiquitination only in the presence of FtBRG1‐C (Figure 7D). This observation elucidates the presence of their interaction region and active sites within FtMYB15L and FtBRG1‐C, corroborating the outcomes of the Y2H study. We next performed protein degradation assays Purified recombinant FtBRG1‐GFP was incubated with total proteins extracted from FtMYB15L hairy roots. The findings observed that FtMYB15L was degraded much faster when co‐incubated with FtBRG1, whereas the FtMYB15L protein in the control incubation (without FtBRG1) remained stable (Figure 7E,F). Moreover, LCI experiments revealed that FtMYB15L binds to FtBRG1 more frequently than FtAGL16 under normal conditions. Conversely, after salt stress treatment, this preference was reversed, with FtMYB15L interacting more strongly with FtAGL16 (Figure 7G and Figure S35, Supporting Information). To further support this hypothesis, we transiently transformed FtMYB15L‐His into *N. benthamiana* leaves and extracted the total plant protein. We subsequently co‐incubated His‐FtMYB15L, GST‐FtAGL16 and GST‐FtBRG1 proteins under various conditions (normal and 100 × 10^−3^ m NaCl) and conducted pull‐down experiments (Figure 7H). The results revealed that under normal conditions, FtMYB15L and FtBRG1 demonstrated a strong binding capability. Upon exposure to salt treatment, the affinity of FtMYB15L for FtBRG1 decreased, while its binding capacity to FtAGL16 was enhanced. This statement implies that FtAGL16 can rival FtBRG1 in its binding to FtMYB15L under conditions of salt stress. This study thus elucidates a competitive binding mechanism whereby FtAGL16 sequesters FtMYB15L from the FtBRG1 ubiquitin ligase complex under salt stress, leading to the stabilization of FtMYB15L and the subsequent transcriptional upregulation of *FtMGT2* to improve plant performance.

### 2.6 Differentiation of *FtMGT2* Conferred Salt Tolerance Difference between Cultivated and Wild Species

Genetic divergence, reflected in DNA sequence variation, is a fundamental driver of population differentiation and speciation.^[^ ^45^ ^]^ Following its domestication in the Himalayan region, cultivated buckwheat dispersed throughout China and was subsequently disseminated globally. As the environment for buckwheat cultivation changed, many resistance genes were likely lost and observation underscored by the fact that wild species of buckwheat generally exhibit higher stress resistance than cultivated species. To validate this hypothesis, we conducted a salt treatment experiment using two cultivated species including *F. tataricum* “Pinku 1” and *F. esculentum* “Xinong 9976” and two wild species including of *F. gracilipes* “Xibing 65” and *F. urophyllum* “Yingzhi.” The results from this experiment indicated that the wild species exhibited significantly better salt tolerance compared to the cultivated species (Figures S36 and S37, Supporting Information).

**Figure 8:**
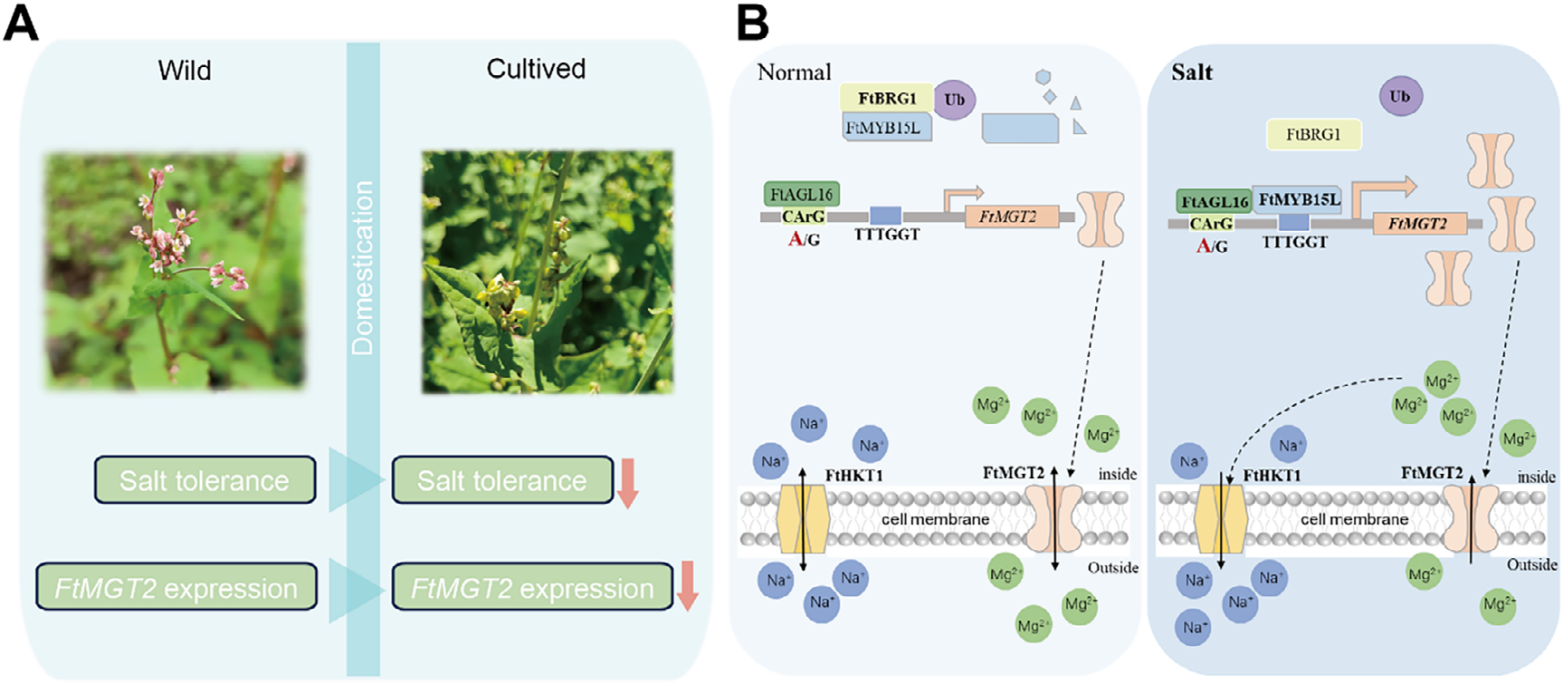
Model of the *FtMGT2* regulatory mechanisms. A) The domestication of wild buckwheat into cultivated buckwheat resulted in a reduction in total salt tolerance and a decrease in *FtMGT2* expression. Wild, wild Tartary buckwheat, cultivated, cultivated Tartary buckwheat. B) FtMGT2 is capable of transporting Mg^2^⁺, and under salt stress conditions, it can transport higher concentrations of Mg^2^⁺ to assist plants in resisting salt stress. Specifically, elevated levels of Mg^2^⁺ enhance the activity of FtHKT1 in Na⁺ efflux, resulting in reduced accumulation of Na⁺ in plants and thereby alleviating salt stress.

To investigate the presence of *FtMGT2* in the differentiation process, we cloned and sequenced the *FtMGT2* gene from these four species. We observed distinct sequence variation in the promoters of cultivated and wild species (Figures S38 and S39, Supporting Information). To further elucidate the impact of *FtMGT2* on salt stress during buckwheat differentiation, each of the four *FtMGT2* promoters were inserted into 0800‐miniLUC vector. Subsequently, the LUC activities were assessed through transient transformation into *N. benthamiana* leaves. The results indicated that the *FtMGT2* promoters from the wild species drove significantly higher LUC activity than those from the cultivated species (Figure S40, Supporting Information). Consequently, this variation in promoter strength may lead to alterations in *FtMGT2* expression, which could be a contributing factor to the significant differences in salt resistance observed between wild and cultivated groups (**Figure** **8**A). Thus, all the above findings suggest that the differentiations of *FtMGT2* between cultivated and wild groups may mediate the plant salt stress response. This differentiation represents a valuable genetic resource that can be harnessed as a breeding aid.

## 3 Discussion

Salt stress significantly constrains global crop production.^[^ ^46^, ^47^ ^]^ Despite the identification of various transporters associated with salt tolerance, the regulatory mechanisms governing their transport activity remain inadequately elucidated.^[^ ^22^ ^]^ Previous research has demonstrated the significance of Mg^2+^ in the growth and development processes of plants, including photosynthesis^[^ ^48^ ^]^ and circadian period maintenance.^[^ ^49^, ^50^, ^51^ ^]^ In addition, Mg^2+^ plays a crucial role in conferring tolerance to environmental stress. Previous studies have shown that the exogenous application of Mg^2+^ significantly enhances salt tolerance in plants subjected to saline stress.^[^ ^22^ ^]^ Mg^2+^ promotes ion balance, improves the Na^+^ conductivity ratio, and supports normal physiological functions. Furthermore, Mg^2+^ supplementation can alleviate oxidative stress and minimize cellular damage caused by salt stress, thus promoting plant growth and development.^[^ ^18^, ^19^ ^]^ The regulation of Na^+^ levels in the aerial parts of plants by magnesium transporters has provided insights into the role of these transporters in salt stress.^[^ ^21^, ^22^ ^]^ In this study, through genome‐wide association study and transcriptomic profiling of the response to salt treatment, we identified FtMGT2, encoding a magnesium transporter, to be significantly associated with salt tolerance in Tartary buckwheat. Genotypic analysis indicated that accessions with higher *FtMGT2* expression exhibited higher salt tolerance. Our comprehensive functional analyses—spanning complementation in mutant yeast, heterologous expression in Arabidopsis seedlings, and overexpression/knockout lines in Tartary buckwheat hairy roots—collectively confirm a critical role for this transporter in plant salt stress tolerance, confirming the positive regulatory role of *FtMGT2* in Tartary buckwheat salt tolerance. Transcriptome analyses unveiled that overexpression of *FtMGT2* resulted in a significant increase in the expression of salt tolerance‐associated genes, such as xyloglucan galactosyltransferase MUR3.^[^ ^52^ ^]^ This study confirmed that FtMGT2 exhibits robust Mg^2+^ transport activity while being incapable of directly transporting Na^+^ and K^+^. Furthermore, FtMGT2 can indirectly influence the activity of the Na^+^ transporter protein FtHKT1 by enhancing Mg^2+^ transport. This mechanism results in increased Na^+^ efflux in plants under salt stress, thereby alleviating the harmful effects of saline conditions. This implies that *FtMGT2* potentially modulates plant salt stress tolerance by regulating the expression of salt tolerance‐associated genes. In addition, the expression of negative regulators of JA signaling was found to increase following *FtMGT2* overexpression. This suggests a potential role of *FtMGT2* in decreasing plant disease tolerance through the regulation of JA signaling.

Previous research has also shown that the expression of *MGTs* is regulated by transcription factors. For example, two central circadian oscillators, *OsPRR59* and *OsPRR95*, were discovered to function as transcriptional repressors, negatively regulating the rhythmic expression of *OsMGT3*, thereby modulating magnesium homeostasis in rice.^[^ ^53^ ^]^ The expression of *OsMGT1* is regulated by an aluminium (Al)‐responsive transcription factor, Al resistance transcription factor 1^[^ ^54^ ^]^. In this study, we discovered that the MYB transcription factor FtMYB15L and MADS transcription factor FtAGL16 can regulate *FtMGT2* expression by directly binding to its promoter. This demonstrates that FtMYB15L and FtAGL16 were crucial regulators in the *FtMGT2*‐dependent salt stress response. We also found FtAGL16 could interact with FtMYB15L, synergistically co‐regulating the transcription of *FtMGT2*. This cooperative regulation is analogous to findings in *Cyanobacteria*, where the two transcription factors *NtcA* and *NtcB* co‐regulate the transcription activation of the nitrate assimilation pathway.^[^ ^55^ ^]^

The amount of transcription factors is strictly regulated by the ubiquitin degradation pathway.^[^ ^56^ ^]^ For instance, the transcription factor Rice Outermost Cell‐Specific 4 (ROC4), which stimulates the expression of *BODYGUARD* and controls plant response to drought, is negatively regulated by the really interesting new gene (RING)‐type E3 ligase gene drought hypersensitive.^[^ ^1^ ^]^ The OsMYBc transcription factor can be ubiquitinated by E3 ligase MYBc Stress‐Related RING Finger Protein (MSRFP), thus regulating the expression of the Na^+^ transporter *OsHKT1;1* in plant salt stress.^[^ ^14^ ^]^ In the present study, through co‐expression network analysis and protein interaction validation, we found that transcription factor FtMYB15L could assemble with and be degraded by FtBRG1, thus regulating the expression of *FtMGT2*. The overexpression of *FtBRG1* in Tartary buckwheat hairy roots indicated that *FtBRG1* exhibited opposite function compared to *FtMGT2* and *FtMYB15L*, and the *brg1‐C* knockout hairy roots exhibited the same function as *FtMGT2* and *FtMYB15L OE hairy roots*, suggesting FtBRG1 participates in plant response to salt stress by precisely regulating the protein level of FtMYB15L, thus regulating *FtMGT2* expression. In addition, transcription factor degradation is also precisely regulated. For instance, in carcinoma of colon and rectum, C4orf19 competes with the E3 ligase TRIM25 for binding to Lys615 of the E3 ligase Keap1, disrupting Keap1 ubiquitination and resulting in an increase in Keap1 protein levels.^[^ ^57^ ^]^ Here, we found FtAGL16 competes with FtBRG1 to bind to FtMYB15L, stabilizing FtMYB15L and promoting its regulation of target gene *FtMGT2*, underscoring that the dynamic regulation of FtMYB15L is crucial in Tartary buckwheat response to salt stress.

Previous research illustrated that flavonoids play an important role in plant tolerance to various stresses, including drought, cold, salt, heavy metal, ultraviolet rays stress, as well as biotic stress.^[^ ^58^, ^59^, ^60^, ^61^ ^]^ Moreover, these flavonoids associated with stress tolerance significantly decreased during crop domestication.^[^ ^33^, ^39^, ^62^ ^]^ In the present research, by analyzing the metabolite content in Tartary buckwheat accessions of different genotypes, we found that natural variation in the promoter of *FtMGT2* is directly correlated with kaempferol content variation in Tartary buckwheat, suggesting that *FtMGT2* might also participate in the regulation of flavonoid metabolism. Furthermore, the exogenous application of kaempferol enhanced Tartary buckwheat's resistance to salt stress, suggesting kaempferol might be involved in *FtMGT2* mediated salt stress response. Differential expression analysis revealed that expression of genes involved in JA signaling was significantly changed in *FtMGT2* overexpression lines. Given that previous research illustrated JA could regulate kaempferol biosynthesis,^[^ ^63^, ^64^ ^]^ we hypothesize that *FtMGT2* might regulate kaempferol content through JA dependent pathway, which requires further study.

In recent years, the identification and utilization of superior genotypes play a critical role in crop breeding.^[^ ^65^ ^]^ For instance, the natural variation on the promoter region of *ZmICE1* was found to be involved in cold tolerance in maize by regulating amino acid metabolism.^[^ ^66^ ^]^ Similarly, natural variation in the *ZmbZIP68* promoter modulates cold tolerance, and it may be a crucial target for breeding cold‐tolerant maize varieties.^[^ ^67^ ^]^ Here, we found the natural variation on the promoter of *FtMGT2* could change *FtMGT2* expression and salt tolerance in Tartary buckwheat. We found that this natural variation is more prominent in the HW populations than in the NL and SL populations, suggesting that this genetic diversity may have undergone selective pressures during the domestication of Tartary buckwheat. We hypothesize this may be due to the higher content of Mg^2+^ and flavonoids in genotype A, which provides the plants with stronger antioxidant capacity and resilience to stress. Therefore, the *FtMGT2* A‐genotype, which confers higher *FtMGT2* expression and superior salt tolerance, represents a critical genetic resource and a valuable target for breeding high‐salt‐resistance Tartary buckwheat.

In conclusion, by integrating analyses of genotypic, phenotypic, and transcriptomic variation, we identified that a natural variation in the promoter of *FtMGT2* is associated with salt tolerance in Tartary buckwheat. This natural variation is located within a MADS transcription factor binding motif, CArG, which can bind By FtAGL16. FtAGL16 can not only bind to the *FtMGT2* promoter but also interact with FtMYB15L, thereby co‐regulating the expression of *FtMGT2*. Under normal conditions, the E3 ubiquitin ligase FtBRG1 promotes the degradation of excess FtMYB15L, alleviating the transcription activation of *FtMGT2*, thus reducing kaempferol content and basal plant salt tolerance. However, under salt stress, FtAGL16 can compete with FtBRG1 for binding to FtMYB15L, resulting in the accumulation of FtMYB15L protein, which in turn promotes *FtMGT2* expression, The high expression of *FtMGT2* results in increased accumulation of Mg^2+^ in plants. Elevated concentrations of Mg^2+^ enhance the Na^+^ transport capacity of FtHKT1, leading to a significant increase in Na^+^ efflux. This, in turn, enables plants to better withstand saline conditions (Figure 8B). These findings not only provide a deep understanding of the salt tolerance mechanism in Tartary buckwheat but also offer significant implications and valuable genetic targets for resistance breeding in other crops.

## 4 Experimental Section

### Methods

All methods are listed in the Supporting Information.

### Statistical Analysis

All statistical analyses were performed using GraphPad Prism 9.0 (GraphPad Software, La Jolla, CA) or SPSS 22.0 (IBM Corp., Armonk, NY). Data are presented as the mean ± SD. The sample size (*n*) for each experiment, representing independent biological replicates, was explicitly stated in the corresponding figure legends. Prior to analysis, all data were tested for normality and homogeneity of variances to ensure the assumptions for the *t*‐test and ANOVA were met. No data pre‐processing or outlier removal was performed. Comparisons between two groups were performed using a two‐sided Student's *t*‐test. Comparisons among three or more groups were performed using one‐way ANOVA followed by Tukey's HSD test. *P* < 0.05 were considered statistically significant. Significance levels are denoted in the figures as follows: *P < 0.05; **P < 0.01; ***P < 0.001; ****P < 0.0001.

## Author Contributions

X.L., Y.H., W.W., Z.L., and Y.G. contributed equally. M.Z. designed and managed the project. M.Z., Y.H., and K.Z. organized the funding for this research. D.L. and W.L. provided the genetic materials. Y.H., K.Z., Z.L., C.H., Y.S., and C.G. performed data analysis and figure design. X.L., S.S., H.Z., W.W., Y.G., W.L, Z.L., O.Y., Y.N., and G.L. performed most of the experiments. X.L., Y.H., K.Z., Y.S., R.J., S.H.W., M.Q., X.L., M.I.G., and A.R.F. wrote the manuscript. All authors read and approved the manuscript.

## Conflict of Interest

The authors declare no conflict of interest.

## Supporting information



Supporting Information

Supporting Information

Supporting Information

Supporting Information

Supporting Information

## Data Availability

All the Pinku 1 RNA‐seq read data and the transgenic *Arabidopsis thaliana* RNA‐seq read data have been deposited at China National Center for Bioinformation (CNCB) with BioProject accession PRJCA023791 and PRJCA023794. All the other data associated with this study are shown in the Supporting Information and Source Data file. Source data are provided with this paper.
