# Supplementary material for: Domestication of Tartary Buckwheat Shaped a Regulatory Module for Seedling Salt Tolerance by Targeting the Magnesium Transporter Gene FtMGT2
Source: Adv Sci (Weinh). 2025 Nov 25;13(8):e11570. doi: 10.1002/advs.202511570 (PMC12884793; doi:10.1002/advs.202511570)
Supplement: Supplementary file 1 — Supporting Information [file ADVS-13-e11570-s003.docx]

**Supplementary Information**

The following materials are available in the online version of this article.

**Figures S1-40**

**Figure S1** **Phenotype of Tartary buckwheat seedlings under 100 mM or 200 mM NaCl treatment.** Pinku1 seeds were subjected to varying salt concentrations (100 mM or 200 mM) to observe their germination and growth. Bar = 2 cm.

**Figure S2** **Volcano plot of the differentially expressed genes of NaCl treatment for 3 hours compared to controls.** The short line exhibited *FtMGT2* was significantly up-regulated after NaCl treatment for 3 hours.

**Figure S3** **KEGG and GO enrichment of up-regulated genes after NaCl treatment compared to control in different times. A)** KEGG enrichment analysis on the genes that were up-regulate following a 3-hour treatment with NaCl. **B)** GO enrichment analysis on the genes that were up-regulate d following a 3-hour treatment with NaCl. **C)** KEGG enrichment analysis on the genes that were up-regulate following a 6-hour treatment with NaCl. **D)** GO enrichment analysis on the genes that were up-regulate following a 6-hour treatment with NaCl. **E)** KEGG enrichment analysis on the genes that were up-regulate following a 12-hour treatment with NaCl. **F)** GO enrichment analysis on the genes that were up-regulate following a 12-hour treatment with NaCl.

**Figure S4 Phylogenetic analysis of *FtMGT2* gene with homologous genes in other species. A)** Construct phylogenetic trees by neighbour joining (NJ), gene labeled red is *FtMGT2.* **B)** Linkage disequilibrium (LD) heatmap of *FtMGT2* with the significant loci in Chr 6. **C)** Six significant SNPs were found on the promoter of *FtMGT2*. **D)** *FtMGT2* expression in different genotypes. G/G: genotype G, G/A: genotype G/A, A/A: genotype A. **E)** Ten samples from various populations were randomly selected for the determination of magnesium content. **F)** Ten samples from various genotypes were randomly selected for the determination of magnesium content. Data in **D**, **E** and **F** are presented as the mean  ± SD from n = 3 independent biological replicates. Each data point (**E** and **F**) on the plot indicates the number of replicates. Statistical analysis was performed using one-way ANOVA analysis with Tukey’s HSD test (Different letters represent significant differences at *P* < 0.05).

**Figure S5** **Sequencing results of the mutant hairy roots show that the *FtMGT2* has been successfully knocked out. A)** *FtMGT2*, *FtMGT2* reference sequence for target site. *mgt2-C1* or *mgt2-C2,* sequencing sequence of *mgt2* knockout hairy roots. **B)** The *FtMGT2* expression in *FtMGT2* OE and knockout hairy roots. **C)** The kaempferol of *FtMGT2* OE and knockout hairy roots. **D)** Mg^2+^ fluxes measured from the root of different hairy roots. Data in **B** and **C** are presented as the mean  ± SD from n = 3 independent biological replicates. Statistical analysis was performed using one-way ANOVA analysis with Tukey’s HSD test (Different letters represent significant differences at *P* < 0.05).

**Figure S6** ***FtMGT2* is capable of regulating Mg^2+^ absorption and the Na^+^ / K^+^ ratio. A)** Mg^2+^ content in different hairy roots. **B)** Na^+^ / K^+^ ratio in different hairy roots. **C)** The efficiency of Mg^2+^ absorption varies across different Tartary buckwheat materials. MS, under MS medium. MS + NaCl, under MS medium with 100 mM NaCl. GZ261, a NL group material. SC247, a HW group material. **D)** The efficiency of Mg^2+^ absorption varies across different hairy roots. Data in **A** and **B** are presented as the mean  ± SD from n = 3 independent biological replicates. Statistical analysis was performed using one-way ANOVA analysis with Tukey’s HSD test (Different letters represent significant differences at *P* < 0.05).

**Figure S7** **Dynamic changes of Mg^2+^ and Na⁺ in *FtMGT2* transgenic hairy roots under different ion treatment.** **A)** The flow rates of Na⁺ in various *FtMGT2* transgenic hairy roots materials under Mg^2+^ treatment. **B)** The flow rates of Na⁺ in various *FtMGT2* transgenic hairy roots under Na⁺ treatment. **C**) The flow rates of Na⁺ in various *FtMGT2* transgenic hairy roots under Na⁺ + Mg^2+^ treatment. Positive values indicate efflux, whereas negative values signify absorption.

**Figure S8 *FtMGT2* and Mg^2+^ can regulate the expression of *FtHKT1* in Tartary buckwheat. A)** The expression levels of *FtHKT1* significantly change in *FtMGT2* transgenic hairy roots. HKT1, reported genes that positively regulate Na^+^ - K^+^ transport. Data are presented as the mean  ± SD from n = 3 independent biological replicates. Statistical analysis was performed using one-way ANOVA analysis with Tukey’s HSD test (Different letters represent significant differences at *P* < 0.05). **B)** The data was derived from the transcriptome of Tartary buckwheat under 50 mM MgSO_4_ treat for different time.

**Figure S9** **Functional validation of *FeMGT2* overexpressing hairy roots under salt stress. A)** The phenotype of A4 and *FeMGT2* overexpressing hairy roots cultured in MS liquid medium (MS) and MS liquid medium + 100 mM NaCl (NaCl) for 20 days. A4, A4 *Agrobacterium rhizogenes* empty strain hairy roots; *FeMGT2-1*, *FeMGT2-2*, and *FeMGT2-3*, three *FeMGT2* overexpressed hairy root strains. Bar = 5 mm. **B)** The fresh weight (FW) of A4 and *FeMGT2* overexpressed hairy roots in **A**. **C)** The kaempferol content of A4 and *FeMGT2* overexpressed hairy roots in **A**. Data in **B** and **C** are presented as the mean  ± SD from n = 3 independent biological replicates. Statistical analysis was performed using one-way ANOVA analysis with Tukey’s HSD test (Different letters represent significant differences at *P* < 0.05).

**Figure S10** **The enzyme activity of *FtMGT2* hairy roots under natural conditions (MS) and suffered to salt stress condition. A)** The enzyme activity of superoxide dismutase (SOD) and **B)** catalase (CAT) when *FtMGT2* hairy roots under natural conditions (MS) and suffered to salt stress condition. Data in **A** and **B** are presented as the mean  ± SD from n = 3 independent biological replicates. Statistical analysis was performed using one-way ANOVA analysis with Tukey’s HSD test (Different letters represent significant differences at *P* < 0.05).

**Figure S11** **The kaempferol content in *FtMGT2* different genotype accessions.** G/G: genotype G (G1), G/A: genotype G/A (G2), A/A: genotype A (G3). Data are presented as the mean  ± SD. Each data point on the plot indicates the number of replicates. Statistical analysis was performed using one-way ANOVA analysis with Tukey’s HSD test (Different letters represent significant differences at *P* < 0.05).

**Figure S12** **Phenotype (A) and root length (B) of external application of kaempferol in Tartary buckwheat subjected to salt stress.** Bar = 2 cm. Data are presented as the mean  ± SD. Each data point on the plot indicates the number of replicates. Statistical analysis was performed using one-way ANOVA analysis with Tukey’s HSD test (Different letters represent significant differences at *P* < 0.05).

**Figure S13** **Phenotype (A) and root length (B) of external application of kaempferol in *Arabidopsis* seedlings subjected to salt stress.** Bar = 1 cm. Data are presented as the mean  ± SD. Each data point on the plot indicates the number of replicates. Statistical analysis was performed using one-way ANOVA analysis with Tukey’s HSD test (Different letters represent significant differences at *P* < 0.05).

**Figure S14** ***FtMGT2* overexpression *Arabidopsis* can enhance plant salt tolerance. A)** The phenotype of *FtMGT2* overexpression *Arabidopsis* treated with different salt concentrations. Bar = 5 mm. **B-C)** The fresh weight **(B)** and dry weight **(C)** of seedlings in **A**. Data in **B** and **C** are presented as the mean  ± SD from n = 3 independent biological replicates. Statistical analysis was performed using one-way ANOVA analysis with Tukey’s HSD test (Different letters represent significant differences at *P* < 0.05).

**Figure S15** **The salt standard treatment experiment demonstrated that the overexpression of *FtMGT2* confers increased salt tolerance.** The content of kaempferol of Col-0 and *FtMGT2* heterologous expression Arabidopsis grown for 10 days in MS and MS solid medium containing 100 mM NaCl. Data are presented as the mean  ± SD from n = 3 independent biological replicates. Statistical analysis was performed using one-way ANOVA analysis with Tukey’s HSD test (Different letters represent significant differences at *P* < 0.05).

**Figure S16** **The salt standard treatment experiment demonstrated that the overexpression of *FtMGT2* confers increased salt tolerance. A)** Mock, control treatment; Salt, treat *Arabidopsis* with a solution containing 100 mM NaCl for 2 weeks; Rehydration, rehydrate *Arabidopsis* for 2 weeks after 2 weeks of salt treatment. Bar = 5 cm. **B)** The survival rate of *Arabidopsis* in **A**. This study was conducted through three independent experiments, with six pots (four plants per pot) allocated to both the wild-type and transgenic lines in each independent experiment. Data from these parallel experiments were utilized to determine the rehydration rates. Data are presented as the mean  ± SD from n = 3 independent biological replicates. Statistical analysis was performed using one-way ANOVA analysis with Tukey’s HSD test (Different letters represent significant differences at *P* < 0.05).

**Figure S17** ***FtMGT2* can mitigate the harm induced by salt stress in the *mgt2* mutant. A)** Mock, control treatment; Salt, treat *Arabidopsis* with a solution containing 100 mM NaCl for 1 weeks. Bar = 5 mm. **B)** The root length of *Arabidopsis* in **A**. Data are presented as the mean  ± SD from n = 3 independent biological replicates. Statistical analysis was performed using one-way ANOVA analysis with Tukey’s HSD test (Different letters represent significant differences at *P* < 0.05).

**Figure S18** **Dynamic changes of Mg^2+^ and Na⁺ in *FtMGT2* transgenic *Arabidopsis* under different ion treatment. A)** The flow rates of Na⁺ in various *FtMGT2* transgenic *Arabidopsis* materials under Mg^2+^ treatment. **B)** The flow rates of Na⁺ in various *FtMGT2* transgenic *Arabidopsis* materials under Na⁺ treatment. **C)** The flow rates of Na⁺ in various *FtMGT2* transgenic *Arabidopsis* materials under Na⁺ + Mg^2+^ treatment. Positive values indicate efflux, whereas negative values signify absorption.

**Figure S19** **Expression levels of *FtMGT2* in different tissues of Tartary buckwheat seedlings.** Data are presented as the mean  ± SD from n = 3 independent biological replicates. Statistical analysis was performed using one-way ANOVA analysis with Tukey’s HSD test (Different letters represent significant differences at *P* < 0.05).

**Figure S20** **The *FtMGT2* promoter exhibits high expression in both roots and mature leaves.** The GUS staining results of the *FtMGT2* promoter-GUS transgenic *Arabidopsis*: **A)** Whole plants, **B)** Flowers and pods, **C)** Stems, **D)** Roots, **E)** Leaves. Bar = 1 cm.

**Figure S21** **The *FtMGT2* promoter of different genotypes exhibits different expression in both roots and leaves. A)** The GUS staining results of the *FtMGT2* promoter of genotype A GUS transgenic *Arabidopsis.* **B)** The GUS staining results of the *FtMGT2* promoter of mutant A (a point mutation in the genotype A promoter sequence transforms it into that of genotype G) GUS transgenic *Arabidopsis.* **C)** The GUS staining results of the *FtMGT2* promoter of genotype G GUS transgenic *Arabidopsis.* **D)** The GUS staining results of the *FtMGT2* promoter of mutant G (a point mutation in the genotype G promoter sequence transforms it into that of genotype A) GUS transgenic *Arabidopsis*. Bar = 1 cm. **E)** The GUS activity of different GUS transgenic *Arabidopsis.* Data are presented as the mean  ± SD from n = 3 independent biological replicates. Statistical analysis was performed using one-way ANOVA analysis with Tukey’s HSD test (Different letters represent significant differences at *P* < 0.05).

**Figure S22** **Phenotype of different yeast strains in different YPD solid medium.** △Ku70, a wild-type yeast strain; △MRS2, mutant yeast strain; △MRS2-MGT2, cover yeast strain. Bar = 2 cm. **A)** The schematic diagram of yeast knockout vector construction. **B)** The growth curves of three yeast strains in YPD and YPD + Mg^2+^ liquid medium, as shown in B, with OD600 values on the vertical axis and proliferation time on the horizontal axis. **C)** The schematic diagram depicts the location of the transmembrane domain in *FtMGT2* and the structural alteration of the *mgt2* mutant after truncating the transmembrane structure. **D)** The growth of four yeast strains in solid medium of YPD, YPD + 100 mM Mg^2+^ and 100 mM YPD + Na^+^. *△MRS2-Ftmgt2*, a complementary yeast strain. **E)** The growth curves of four yeast strains in YPD + Mg liquid medium.

**Figure S23** **TEVC experiments were performed using *X. laevis* oocyte*s* injected with water, *FtMGT2* cRNA or *FtHKT1* cRNA in the presence of different concentrations of Ca^2+^.**  **A)** A current profile of water-injected or *FtHKT1* oocytes, bathed in different concentrations of Ca^2+^. **B)** Current-voltage relationships of oocytes injected with water or *FtHKT1* cRNA. **C-D)** TEVC of water-injected or *FtHKT1* *Xenopus laevis* oocytes showing no effect of Ca^2+^ (100 µM) on endogenous currents membrane potential (EM). Data in **B** and **D** are presented as the mean ± SD from n = 3 independent biological replicates. Statistical significance was determined using a two-sided Student's *t*-test. Asterisks indicate significant differences.

**Figure S24 Steady-state Na⁺ and Mg^2+^ fluxes of *FtHKT1* transgenic plants under different treatment at the final peaks were calculated. A)** Sequencing results of the mutant hairy roots show that the *FtHKT1* has been successfully knocked out. *FtHKT1,* reference sequence for target site. *hkt1-C*, sequence of the target site in *hkt1-C* mutant hairy roots. **B)** The flow rates of Na⁺ in various *FtHKT1* transgenic *Arabidopsis* materials under different treatment. **C)** The flow rates of Mg^2+^ in various *FtHKT1* transgenic *Arabidopsis* materials under different treatment. **D)** The flow rates of Na⁺ in various *FtHKT1* transgenic Tartary buckwheat hairy roots materials under different treatment. **E)** The flow rates of Mg^2+^ in various *FtHKT1* transgenic Tartary buckwheat hairy roots materials under different treatment. Positive values indicate efflux, whereas negative values signify absorption. Data in **B**, **C**, **D** and **E** are presented as the mean ± SD from n = 3 independent biological replicates. Statistical analysis was performed using one-way ANOVA analysis with Tukey’s HSD test (Different letters represent significant differences at *P* < 0.05).

**Figure S25** **Sequencing results of the mutant hairy roots show that the *FtAGL16* has been successfully knocked out. A-B)** *FtMGT2* and *FtAGL16* reference sequence for target site. **C)** *agl16-C* or *mgt2-C / agl16-C*，sequence of the target site in *agl16-C* and *mgt2-C / agl16-C* mutant hairy roots. **D)** Relative expression of *FtAGL16* in *FtAGL16* OE hairy roots and knockout hairy roots. **E)** Fresh weight of *FtAGL16* OE hairy roots and knockout hairy roots. Data in **D** and **E** are presented as the mean ± SD from n = 3 independent biological replicates. Statistical analysis was performed using one-way ANOVA analysis with Tukey’s HSD test (Different letters represent significant differences at *P* < 0.05).

**Figure S26** **The sequence (A) and position (B) of predicted FtMYB15L binding motif on *FtMGT2* promoter.**

**Figure S27** **The subcellular localization of FtMYB15L.** FtMYB15L-GFP, pCAMBIA1300-FtMYB15L-GFP materials channel; H2B-mCherry, nuclear marker; Bright, bright channel; Merge, merge channel. Bar = 10 μm.

**Figure S28** **Sequencing results of the mutant hairy roots show that the *FtMYB15L* has been successfully knocked out. A-B)** *FtMGT2* and *FtMYB15L* reference sequence for target site. **C)** *myb15l-C* or *mgt2-C / myb15l-C*，sequence of the target site in *myb15l-C* and *mgt2-C / myb15l-C* mutant hairy roots. **D)** Relative expression of *FtMYB15L* in *FtMYB15L* OE hairy roots and knockout hairy roots. **E)** Relative expression of *FtMGT2* in *FtMYB15L* OE hairy roots and knockout hairy roots. Data in **D** and **E** are presented as the mean ± SD from n = 3 independent biological replicates. Statistical analysis was performed using one-way ANOVA analysis with Tukey’s HSD test (Different letters represent significant differences at *P* < 0.05).

**Figure S29 The kaempferol and Mg content of A4 hairy roots, *FtMYB15L* overexpressed and knockout hairy roots. A)** The kaempferol content of different hairy roots. **B)** The Mg^2+^ content of different hairy roots. A4, A4 *Agrobacterium rhizogenes* empty strain hairy roots; *FtMYB15L-1*, *FtMYB15L-2*, and *FtMYB15L-3*, three *FtMYB15L* overexpressed hairy root strains, *myb15-C*, *FtMYB15L* knockout hairy roots; *mgt2-C / myb15l-C*, double mutant hairy roots of *FtMGT2* and *FtMYB15L*. Data in **A** and **B** are presented as the mean ± SD from n = 3 independent biological replicates. Statistical analysis was performed using one-way ANOVA analysis with Tukey’s HSD test (Different letters represent significant differences at *P* < 0.05).

**Figure S30** **The phenotype of Col-0 and *FtMYB15L* overexpressed *Arabidopsis thaliana* grown for 10 days in MS solid medium and MS solid medium + 100 mM NaCl. A)** Col-0, wild-type *Arabidopsis*, *FtMYB5L-1*, *FtMYB15L-2*, *FtMYB15L-3*, three lines of *FtMYB15L* heterologous expression *Arabidopsis*. Bar = 5 mm. **B)** The root length (cm) under **A** condition. **C)** The content of kaempferol under **A** condition. Data in **B** and **C** are presented as the mean ± SD from n = 3 independent biological replicates. Statistical analysis was performed using one-way ANOVA analysis with Tukey’s HSD test (Different letters represent significant differences at *P* < 0.05).

**Figure S31** **The salt standard treatment experiment demonstrated that the overexpression of *FtMYB15L* confers increased salt tolerance. A)** Mock, control treatment; Salt, treat *Arabidopsis* with a solution containing 100 mM NaCl for 2 weeks; Rehydration, rehydrate *Arabidopsis* for 2 weeks after 2 weeks of salt treatment. Bar = 5 cm. **B)** The survival rate of *Arabidopsis* in **A**. This study was conducted through three independent experiments, with six pots (four plants per pot) allocated to both the wild-type and transgenic lines in each independent experiment. Data from these parallel experiments were utilized to determine the rehydration rates. Data are presented as the mean ± SD from n = 3 independent biological replicates. Statistical analysis was performed using one-way ANOVA analysis with Tukey’s HSD test (Different letters represent significant differences at *P* < 0.05).

**Figure S32** **Yeast two hybridization identified candidate E3 ligase interacted with FtMYB15L.** U1, FtPinG0606089000.01, FtBRG1; U2, FtPinG0100616700.01； U3, FtPinG0606392400.01; U4, FtPinG0404214400.01; U5, FtPinG0302633400.01.

**Figure S33** **The subcellular localization of FtBRG1.** FtBRG1-GFP, pCAMBIA1300-FtBRG1 recombination plasmid; H2B-mCherry, nuclear marker; Bright, bright channel; Merge, merge channel. Bar = 10 μm.

**Figure S34** **Sequencing results of the mutant hairy roots show that the *FtBRG1* has been successfully knocked out. A)** *FtBRG1*, *FtBRG1* reference sequence for target site. *brg1-C,* sequence of the *FtBRG1* target site in *brg1-C* mutant hairy roots. **B)** Relative expression of *FtBRG1* in *FtBRG1* OE hairy roots and knockout hairy roots. **C)** Relative expression of *FtMGT2* in *FtBRG1* OE hairy roots and knockout hairy roots. Bar data showed means ± SD. * indicate significant differences at *P* < 0.05. **D)** The fresh weight was observed in A4 hairy roots, *FtBRG1* overexpressed and knockout hairy roots both under normal conditions and after exposure to 100 mM NaCl salt stress for 2 weeks, A4, A4 *Agrobacterium rhizogenes* empty strain hairy roots; *FtBRG1-1*, *FtBRG1-2*, and *FtBRG1-3*, three *FtBRG1* overexpressed hairy root strains. **E)** The kaempferol content of A4 hairy roots, *FtBRG1* overexpressed and knockout hairy roots. Data in **B**, **C**, **D** and **E** are presented as the mean ± SD from n = 3 independent biological replicates. Statistical analysis was performed using one-way ANOVA analysis with Tukey’s HSD test (Different letters represent significant differences at *P* < 0.05).

**Figure S35** **Relative LUC activity under** **Figure 7G.** Mock, under normal condition. NaCl, under 100 mM NaCl condition. Data are presented as the mean ± SD from n = 3 independent biological replicates. Statistical analysis was performed using one-way ANOVA analysis with Tukey’s HSD test (Different letters represent significant differences at *P* < 0.05).

**Figure S36** **Phenotypes of cultivated species and wild species under salt stress.** Two cultivated species including *F. tataricum* 'Pinku 1' and *F. esculentum* 'Xinong9976’, two wild species including *F. gracilipes* 'Xibing 65' and *F. urophyllum* 'Yingzhi’. Normal, under water condition. Salt, under 100 mM NaCl stress. Bar = 1 cm.

**Figure S37** **The relative root elongation of cultivated species and wild species under salt stress.** Two cultivated species including *F. tataricum* 'Pinku 1' and *F. esculentum* 'Xinong9976’, two wild species including *F. gracilipes* 'Xibing 65' and *F. urophyllum* 'Yingzhi’. The relative root elongation of four species in **Figure S36.** Data are presented as the mean ± SD from n = 3 independent biological replicates. Statistical analysis was performed using one-way ANOVA analysis with Tukey’s HSD test (Different letters represent significant differences at *P* < 0.05).

**Figure S38** **The difference sequence of *FtMGT2* promoter in cultivated species and wild species.**

**Figure S39** **Phylogenetic analysis of FtMGT2 gene with four species.**

**Figure S40** **The relative LUC activity between four species.** Two cultivated species including *F. tataricum* 'Pinku 1' and *F. esculentum* 'Xinong9976’, two wild species including *F. gracilipes* 'Xibing 65' and *F. urophyllum* 'Yingzhi’. The relative LUC activity of *FtMGT2*pro-mini-LUC from four species. Data are presented as the mean ± SD from n = 3 independent biological replicates. Statistical analysis was performed using one-way ANOVA analysis with Tukey’s HSD test (Different letters represent significant differences at *P* < 0.05).

**Tables 1-9**

**Table S1** **Differentially expressed genes during Tartary buckwheat treated with NaCl.**

**Table S2** **Salt tolerance index of Tartary buckwheat accessions.**

**Table S3** **Candidate genes in the significant locus.**

**Table S4** **The genes annotation of HW-SL *F_ST_* top 5% intervals.**

**Table S5 The genes annotation of HW-NL *F_ST_* top 5% intervals.**

**Table S6 Putative selective sweeps between HW and SL based on XP-CLR.**

**Table S7 The Mg^2+^ content of different Tartary buckwheat materials.**

**Table S8** **Co-expression genes with *FtMGT2* in transcriptome of Tartary buckwheat treated with NaCl.**

**Table S9** **Primers used in this study.**
