# Supplementary material for: Domestication of Tartary Buckwheat Shaped a Regulatory Module for Seedling Salt Tolerance by Targeting the Magnesium Transporter Gene FtMGT2
Source: Adv Sci (Weinh). 2025 Nov 25;13(8):e11570. doi: 10.1002/advs.202511570 (PMC12884793; doi:10.1002/advs.202511570)
Supplement: Supplementary file 2 — Supporting Information [file ADVS-13-e11570-s002.pdf]

NaCl (mM) 0 100 200

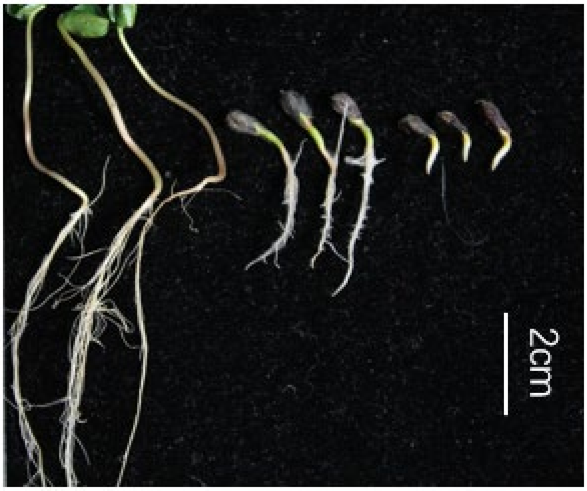

Figure S1 Phenotype of Tartary buckwheat seedlings under 100 mM or 200 mM NaCl treatment. Pinku1 seeds were subjected to varying salt concentrations (100 mM or 200 mM) to observe their germination and growth. Bar = 2 cm.

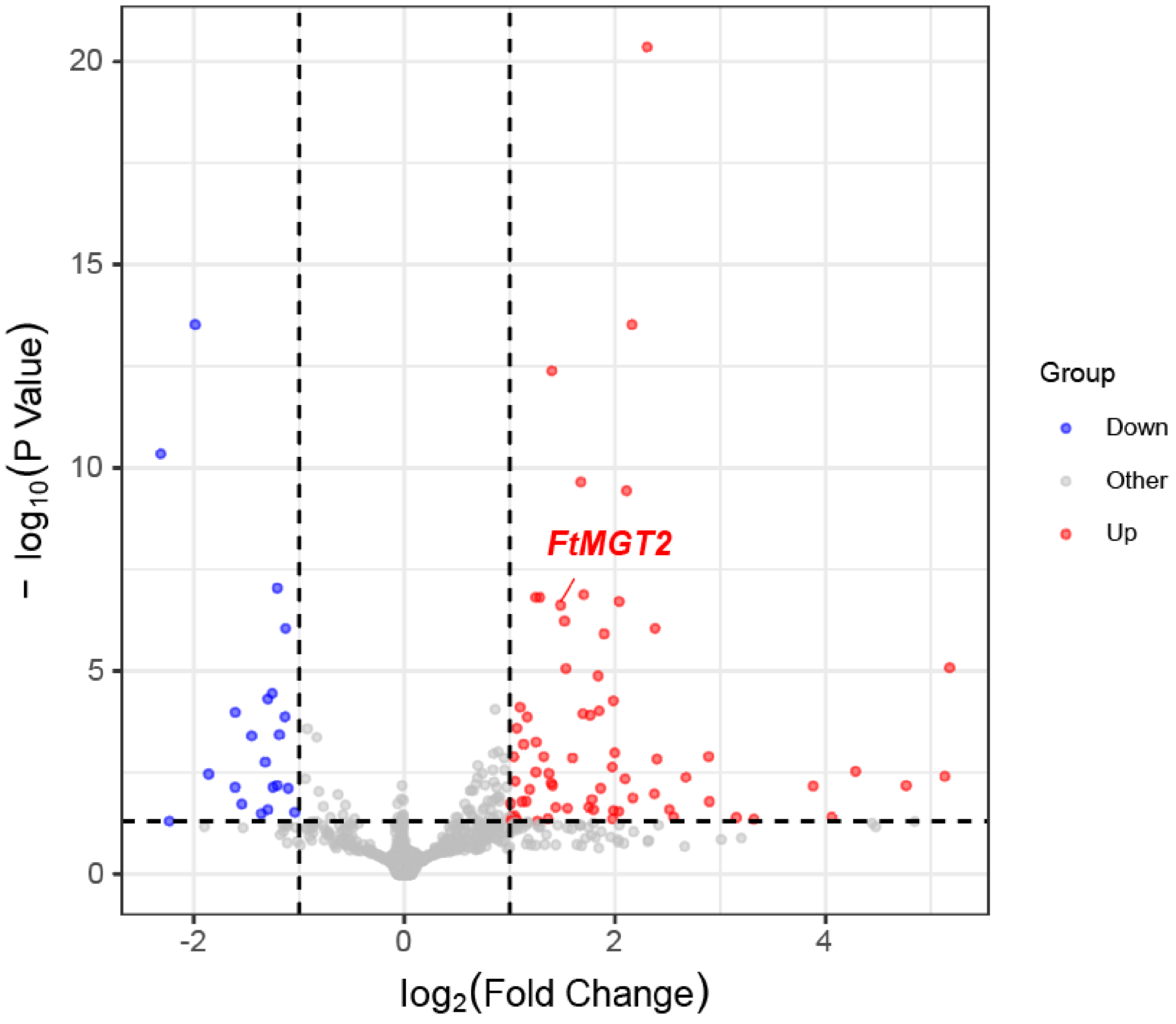

Figure S2 Volcano plot of the differentially expressed genes of NaCl treatment for 3 hours compared to controls. The short line exhibited *FtMGT2* was significantly up-regulated after NaCl treatment for 3 hours.

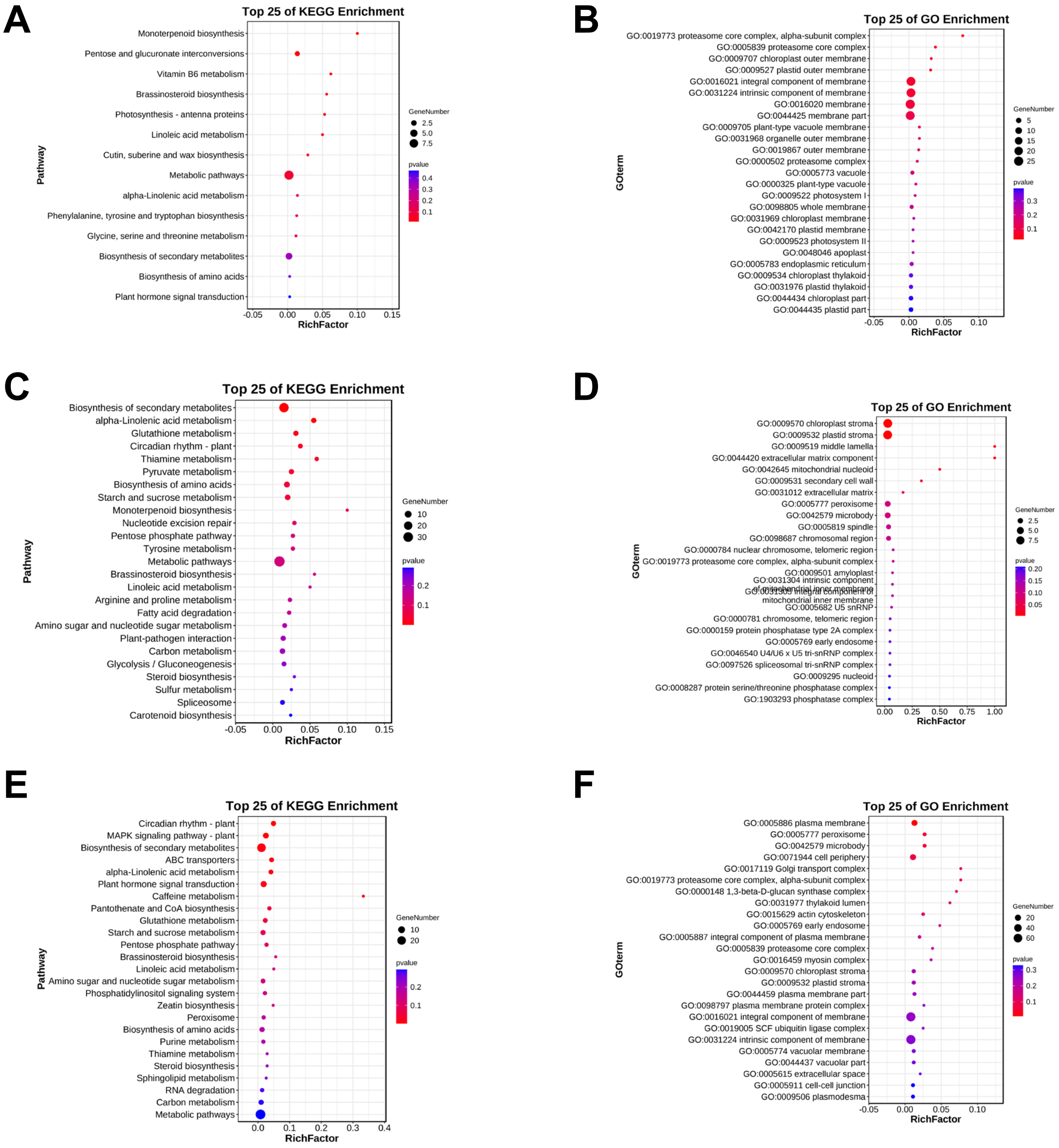

Figure S3 KEGG and GO enrichment of up-regulated genes after NaCl treatment compared to control in different times. A) KEGG enrichment analysis on the genes that were up-regulate following a 3-hour treatment with NaCl. **B)** GO enrichment analysis on the genes that were up-regulate following a 3-hour treatment with NaCl. **C)** KEGG enrichment analysis on the genes that were up-regulate following a 6-hour treatment with NaCl. **D)** GO enrichment analysis on the genes that were up-regulate following a 6-hour treatment with NaCl. **E)** KEGG enrichment analysis on the genes that were up-regulate following a 12-hour treatment with NaCl. **F)** GO enrichment analysis on the genes that were up-regulate following a 12-hour treatment with NaCl.

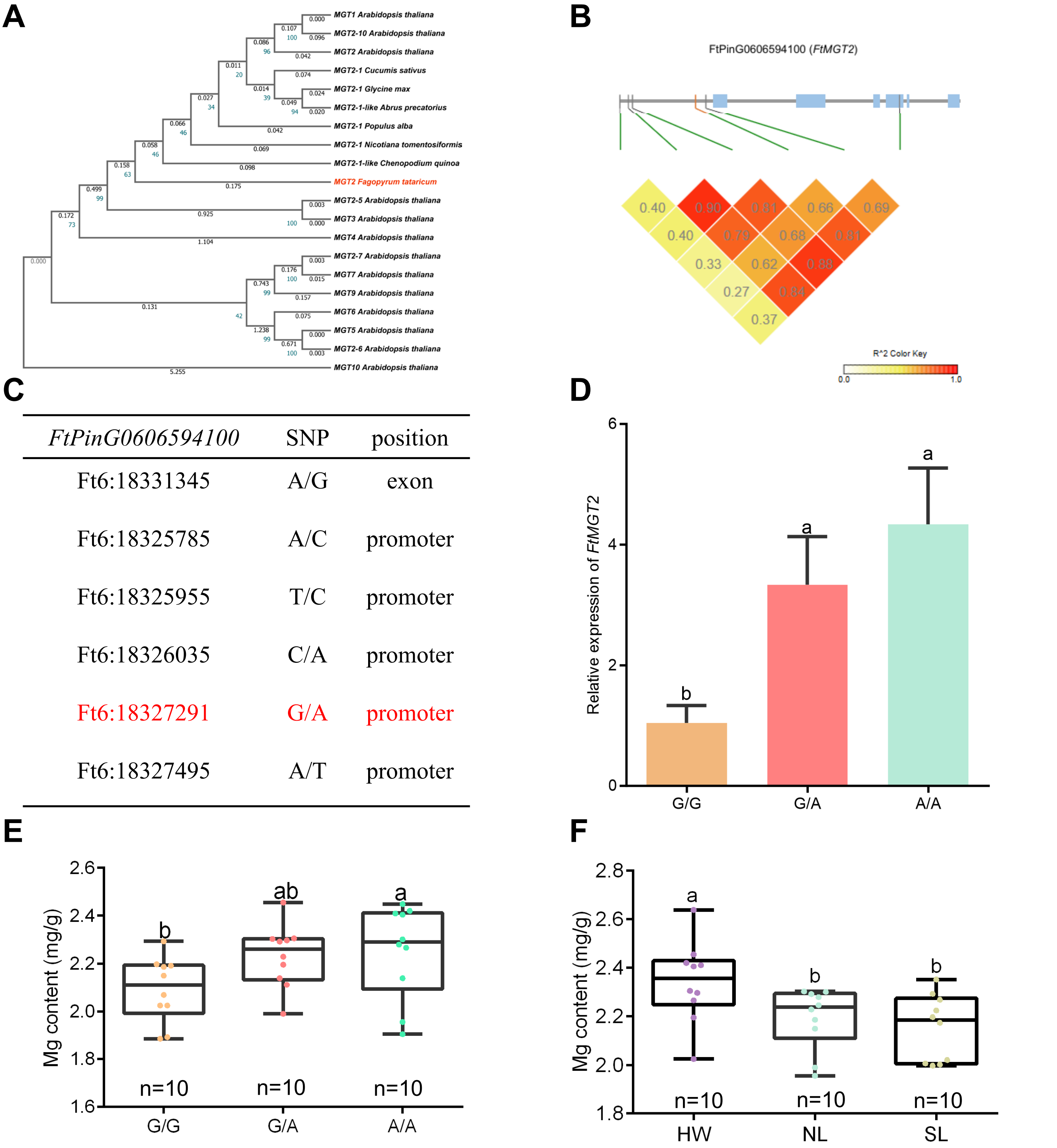

Figure S4 Phylogenetic analysis of *FtMGT2* gene with homologous genes in other species. **A)** Construct phylogenetic trees by neighbour joining (NJ), gene labeled red is *FtMGT2*. **B)** Linkage disequilibrium (LD) heatmap of *FtMGT2* with the significant loci in Chr 6. **C)** Six significant SNPs were found on the promoter of *FtMGT2*. **D)** *FtMGT2* expression in different genotypes. G/G: genotype G, G/A: genotype G/A, A/A: genotype A. **E)** Ten samples from various populations were randomly selected for the determination of magnesium content. **F)** Ten samples from various genotypes were randomly selected for the determination of magnesium content. Data in **D**, **E** and **F** are mean ± SD, n means the accessions numbers. Each data point (**E** and **F**) on the plot indicates the number of replicates. Statistical analysis was performed using one-way ANOVA analysis with Tukey's HSD test (Different letters represent significant differences at $P < 0.05$).

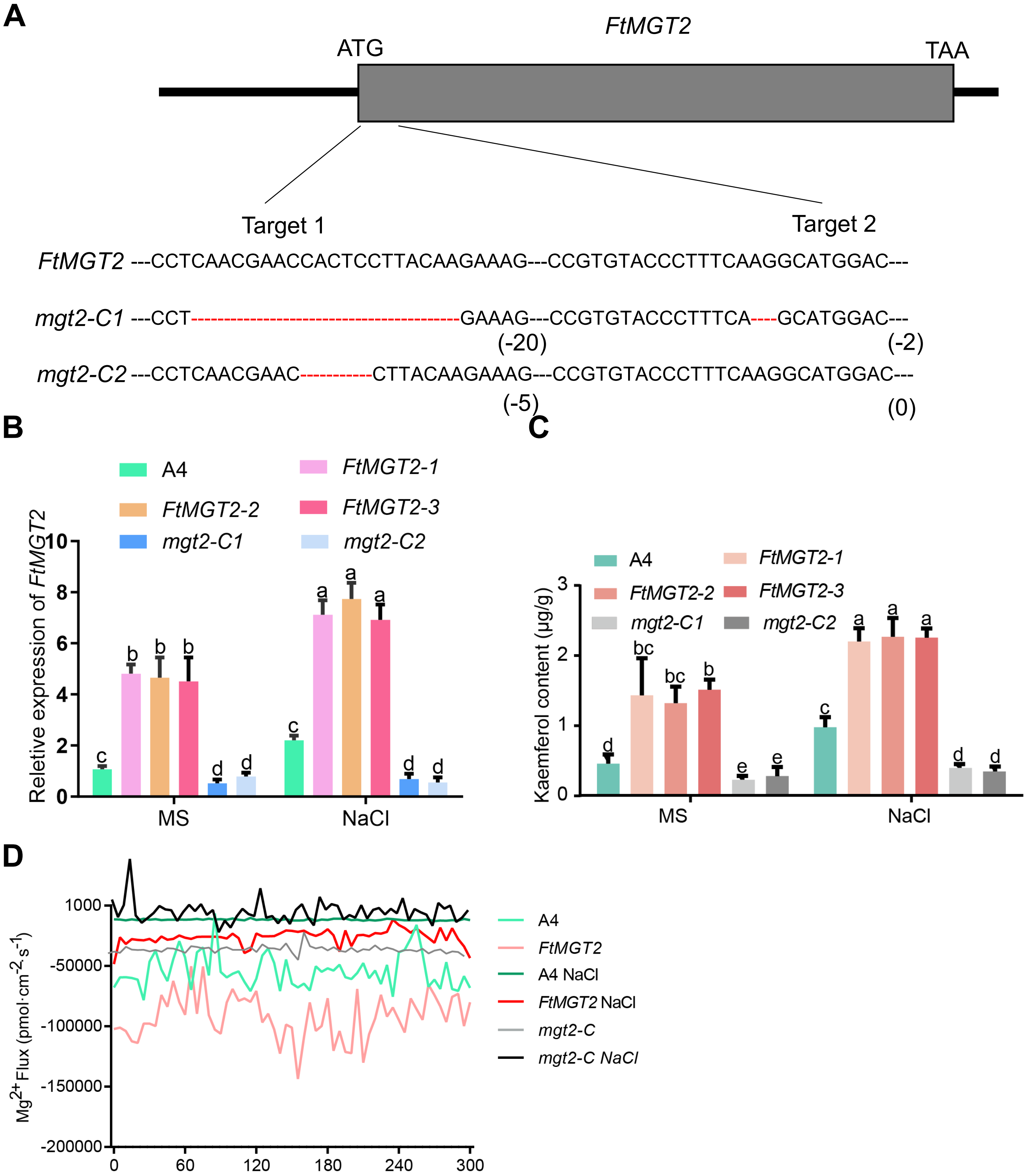

Figure S5 Sequencing results of the mutant hairy roots show that the *FtMGT2* has been successfully knocked out. A) *FtMGT2*, *FtMGT2* reference sequence for target site. *mgt2-C1* or *mgt2-C2,* sequencing sequence of *mgt2* knockout hairy roots. **B)** The *FtMGT2* expression in *FtMGT2* OE and knockout hairy roots. **C)** The kaempferol of *FtMGT2* OE and knockout hairy roots. **D)** Mg^{2+} fluxes measured from the root of different hairy roots. Data in **B and C** are presented as the mean ± SD from n = 3 independent biological replicates. Statistical analysis was performed using one-way ANOVA analysis with Tukey's HSD test (Different letters represent significant differences at $P < 0.05$).

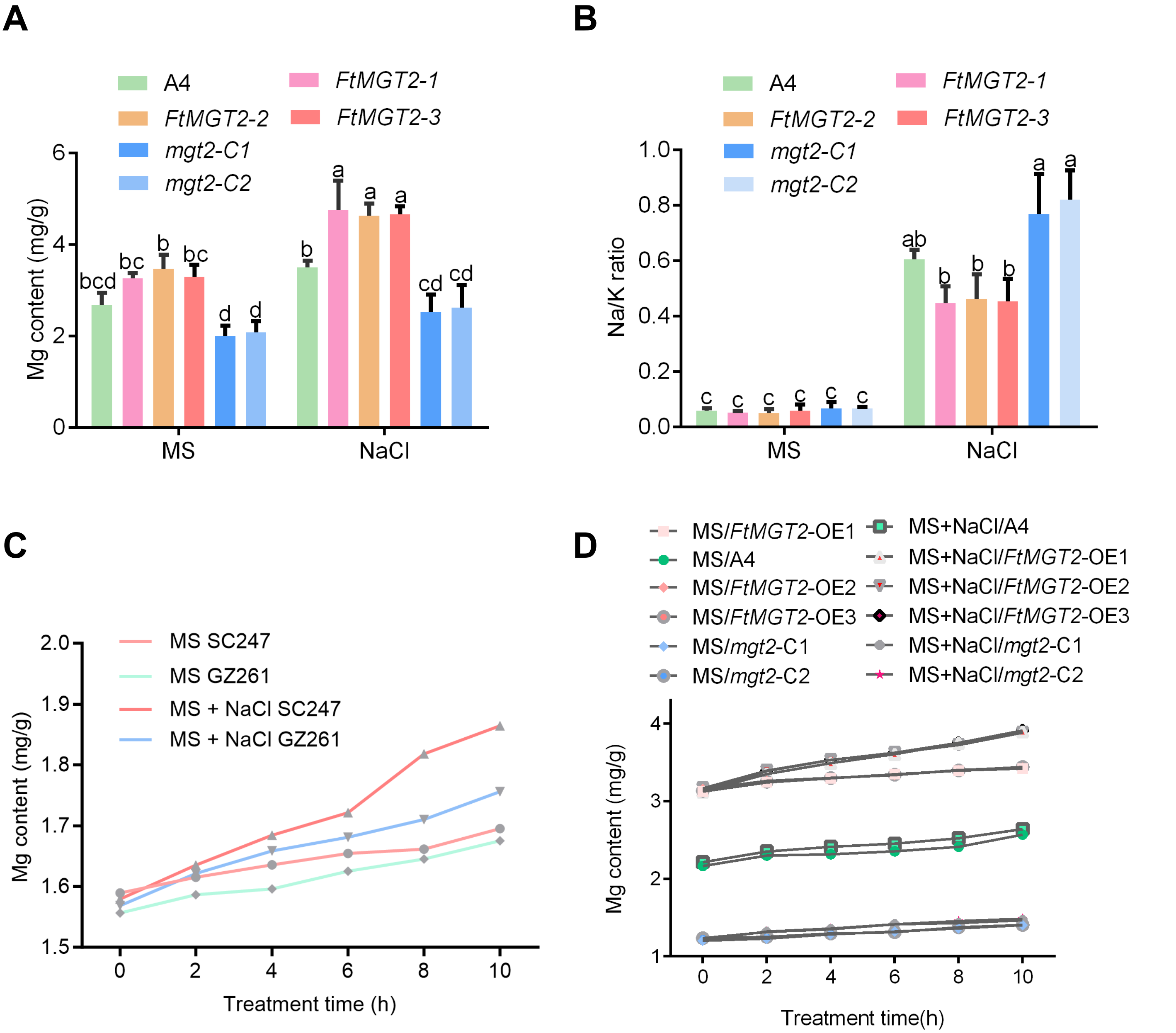

Figure S6 *FtMGT2* is capable of regulating Mg^{2+} absorption and the Na^+ / K^+ ratio. A) Mg^{2+} content in different hairy roots. **B)** Na^+ / K^+ ratio in different hairy roots. **C)** The efficiency of Mg^{2+} absorption varies across different Tartary buckwheat materials. MS, under MS medium. MS + NaCl, under MS medium with 100 mM NaCl. GZ261, a NL group material. SC247, a HW group material. **D)** The efficiency of Mg^{2+} absorption varies across different hairy roots. Data in **A** and **B** are presented as the mean ± SD from n = 3 independent biological replicates. Statistical analysis was performed using one-way ANOVA analysis with Tukey's HSD test (Different letters represent significant differences at $P < 0.05$).

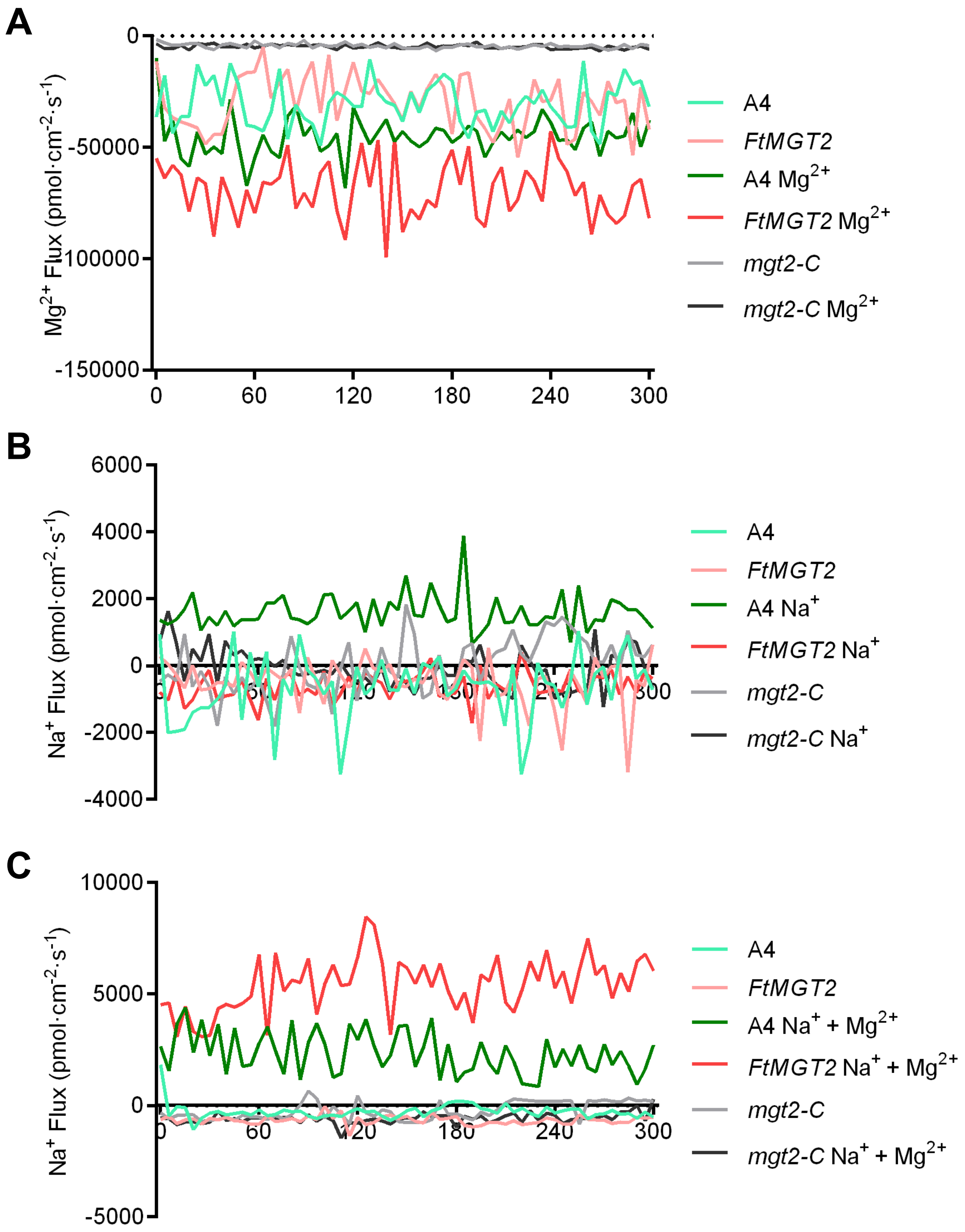

Figure S7 Dynamic changes of Mg^{2+} and Na^{+} in *FtMGT2* transgenic hairy roots under different ion treatment. A) The flow rates of Na^{+} in various *FtMGT2* transgenic hairy roots materials under Mg^{2+} treatment. **B)** The flow rates of Na^{+} in various *FtMGT2* transgenic hairy roots under Na^{+} treatment. **C)** The flow rates of Na^{+} in various *FtMGT2* transgenic hairy roots under Na^{+} + Mg^{2+} treatment. Positive values indicate efflux, whereas negative values signify absorption.

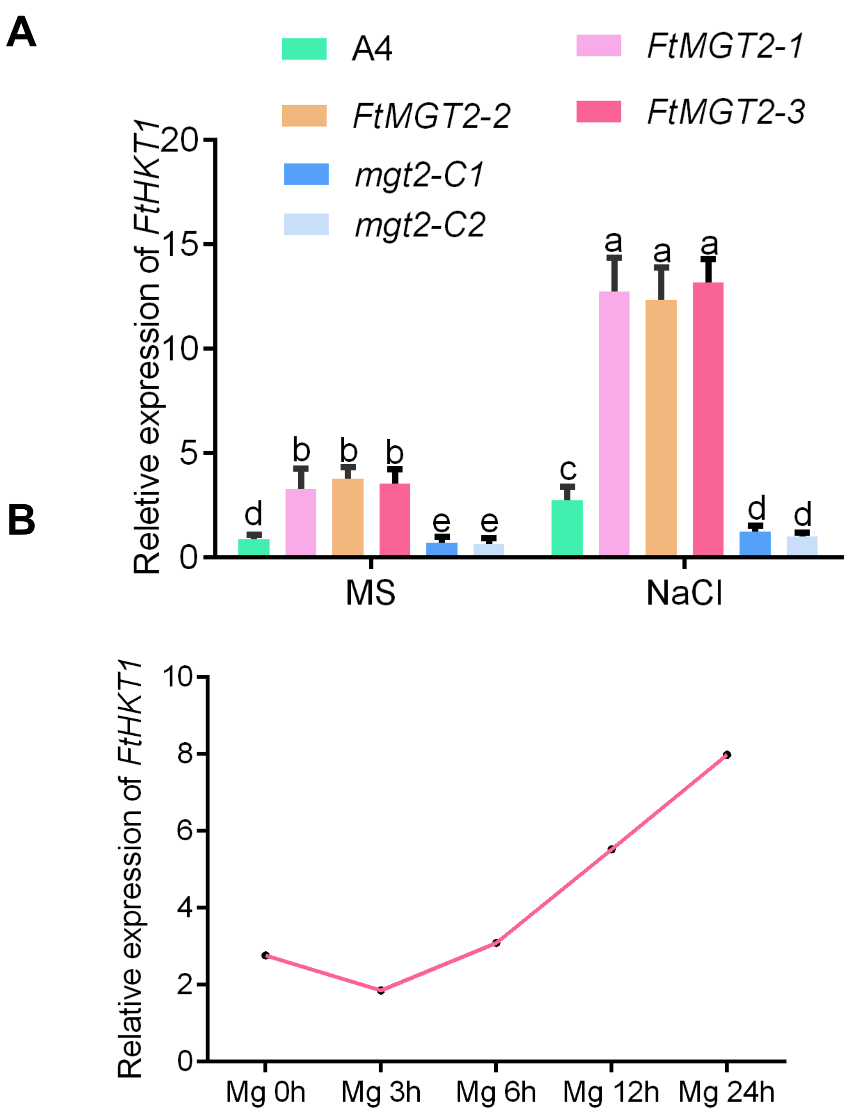

Figure S8 *FtMGT2* and Mg^{2+} can regulate the expression of *FtHKT1* in Tartary buckwheat. A) The expression levels of *FtHKT1* significantly change in *FtMGT2* transgenic hairy roots. HKT1, reported genes that positively regulate Na^{+} - K^{+} transport. Data are presented as the mean ± SD from n = 3 independent biological replicates. Statistical analysis was performed using one-way ANOVA analysis with Tukey's HSD test (Different letters represent significant differences at $P < 0.05$). **B**) The data was derived from the transcriptome of Tartary buckwheat under 50 mM $MgSO_4$ treat for different time.

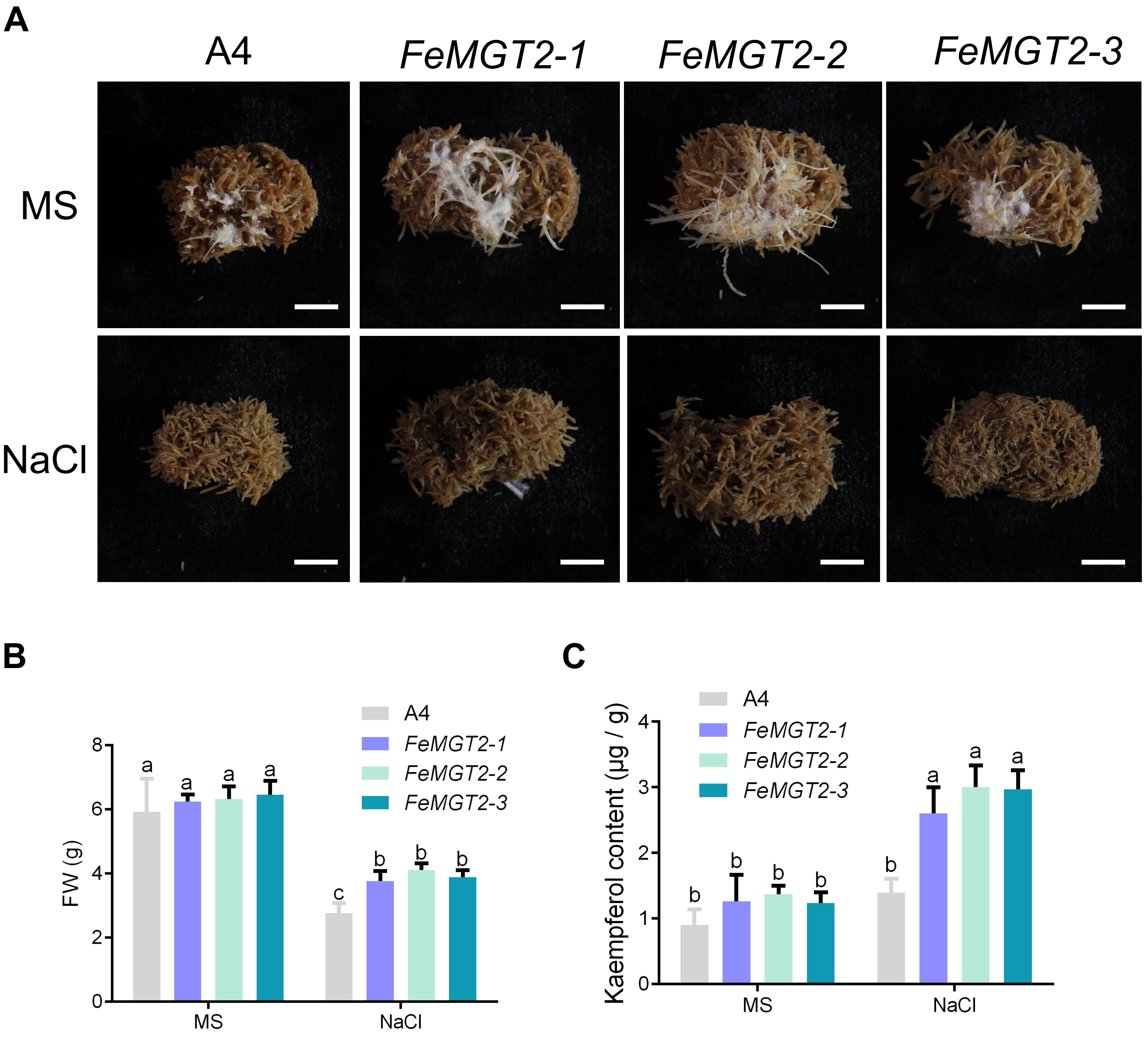

Figure S9 Functional validation of *FeMGT2* overexpressing hairy roots under salt stress. A) The phenotype of A4 and *FeMGT2* overexpressing hairy roots cultured in MS liquid medium (MS) and MS liquid medium + 100 mM NaCl (NaCl) for 20 days. A4, A4 *Agrobacterium rhizogenes* empty strain hairy roots; *FeMGT2-1*, *FeMGT2-2*, and *FeMGT2-3*, three *FeMGT2* overexpressed hairy root strains. Bar = 5 mm. **B)** The fresh weight (FW) of A4 and *FeMGT2* overexpressed hairy roots in **A**. **C)** The kaempferol content of A4 and *FeMGT2* overexpressed hairy roots in **A**. Data in **B** and **C** are presented as the mean ± SD from n = 3 independent biological replicates. Statistical analysis was performed using one-way ANOVA analysis with Tukey's HSD test (Different letters represent significant differences at $P < 0.05$).

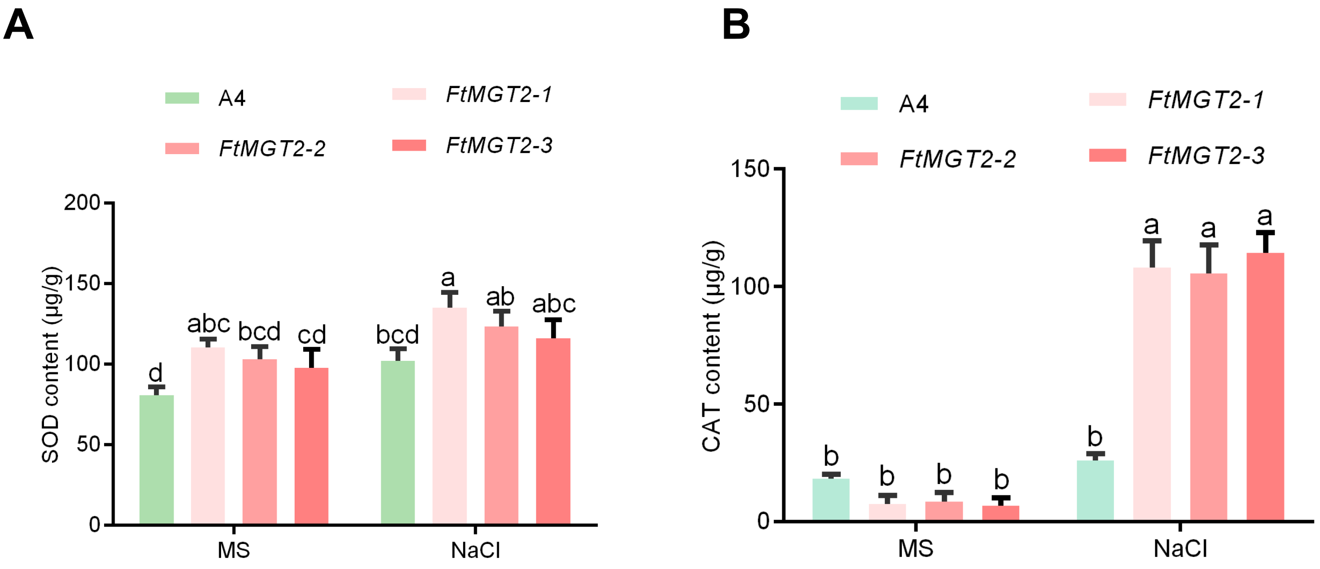

Figure S10 The enzyme activity of *FtMGT2* hairy roots under natural conditions (MS) and suffered to salt stress condition. A) The enzyme activity of superoxide dismutase (SOD) and **B)** catalase (CAT) when *FtMGT2* hairy roots under natural conditions (MS) and suffered to salt stress condition. Data in **A** and **B** are presented as the mean ± SD from n = 3 independent biological replicates. Statistical analysis was performed using one-way ANOVA analysis with Tukey's HSD test (Different letters represent significant differences at $P < 0.05$).

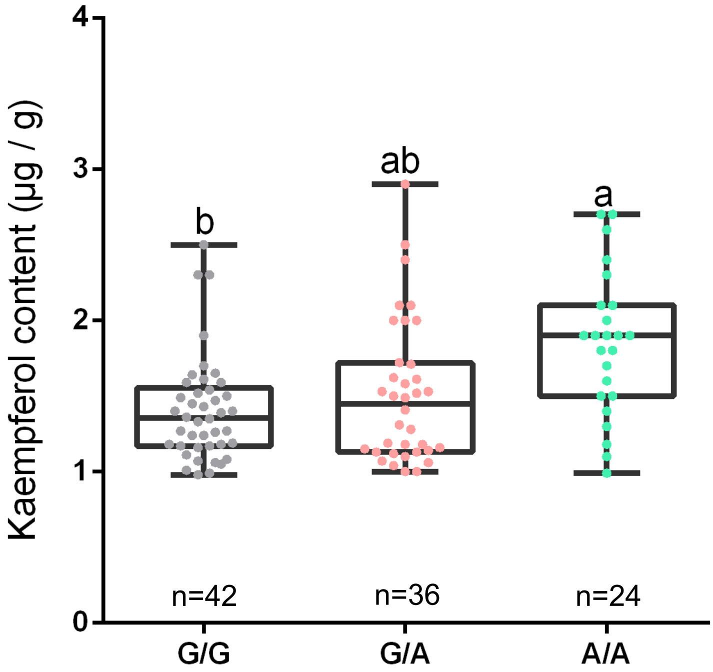

Figure S11 The kaempferol content in *FtMGT2* different genotype accessions. G/G: genotype G (G1), G/A: genotype G/A (G2), A/A: genotype A (G3). Each data point on the plot indicates the number of replicates. Data are presented as the mean ± SD. Each data point on the plot indicates the number of replicates. Statistical analysis was performed using one-way ANOVA analysis with Tukey's HSD test (Different letters represent significant differences at $P < 0.05$).

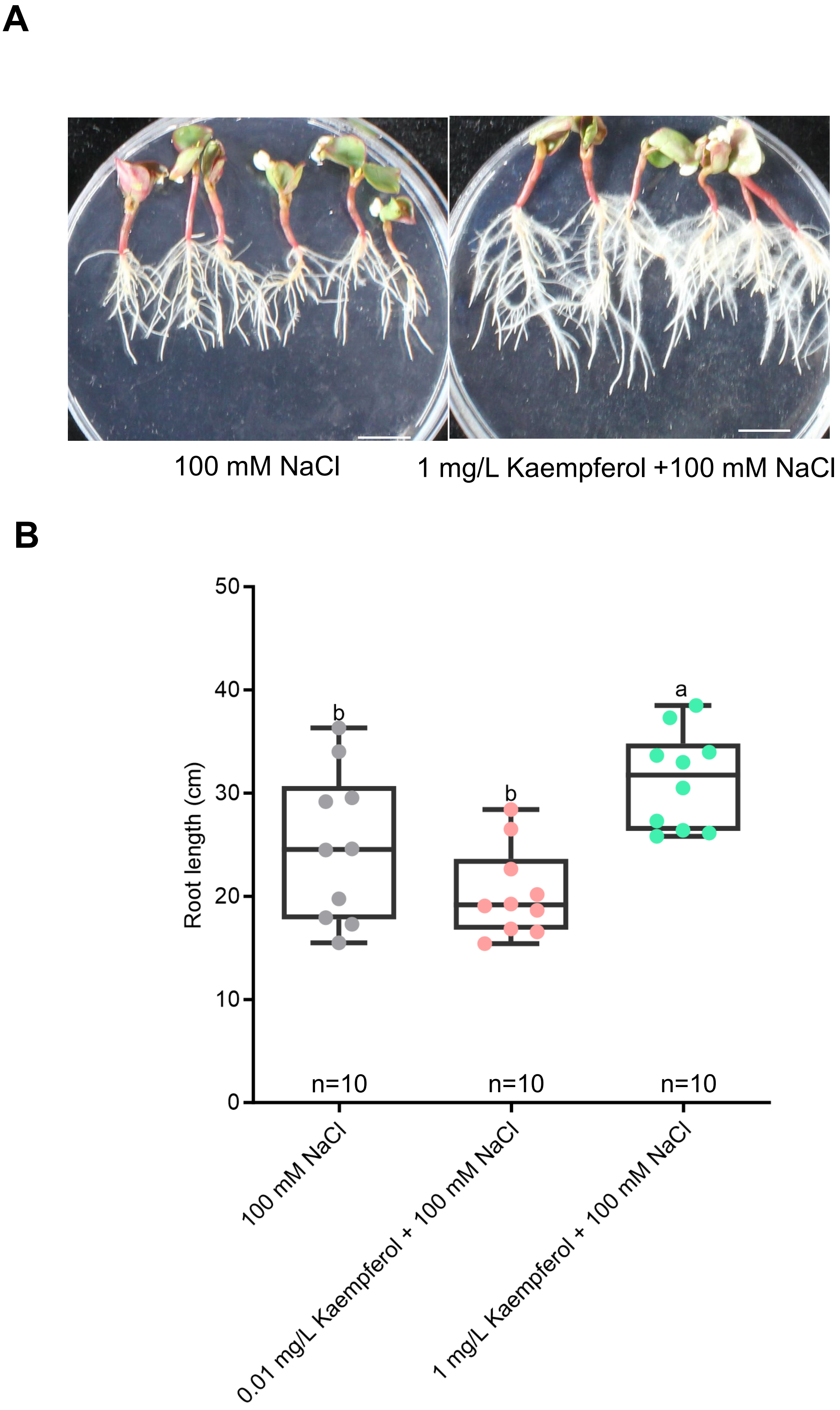

Figure S12 Phenotype (A) and root length (B) of external application of kaempferol in Tartary buckwheat subjected to salt stress. Bar = 2 cm. Data are presented as the mean ± SD. Each data point on the plot indicates the number of replicates. Statistical analysis was performed using one-way ANOVA analysis with Tukey's HSD test (Different letters represent significant differences at $P < 0.05$).

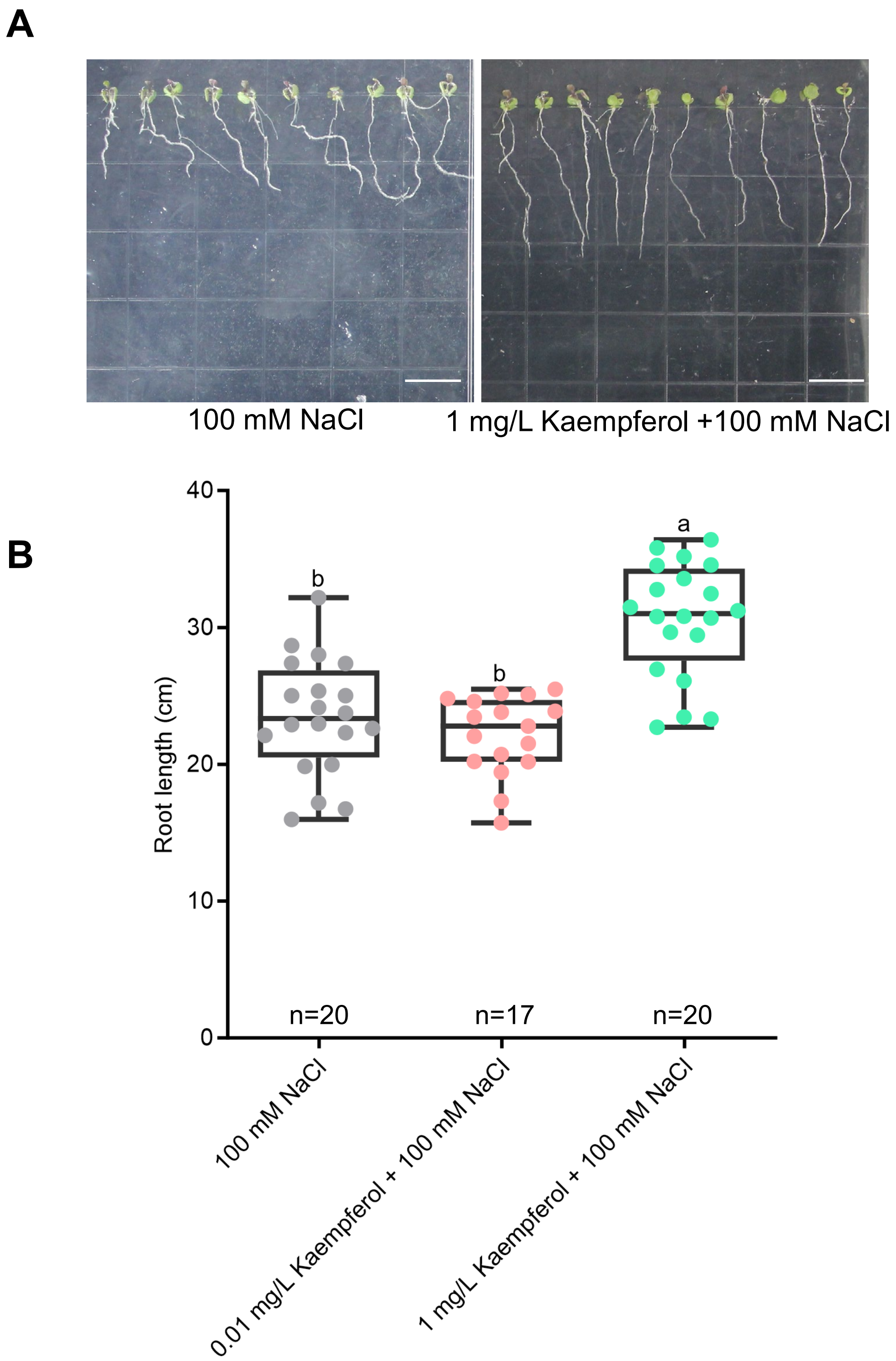

Figure S13 Phenotype (A) and root length (B) of external application of kaempferol in *Arabidopsis* seedlings subjected to salt stress. Bar = 1 cm. Data are presented as the mean ± SD. Each data point on the plot indicates the number of replicates. .Statistical analysis was performed using one-way ANOVA analysis with Tukey's HSD test (Different letters represent significant differences at $P < 0.05$).

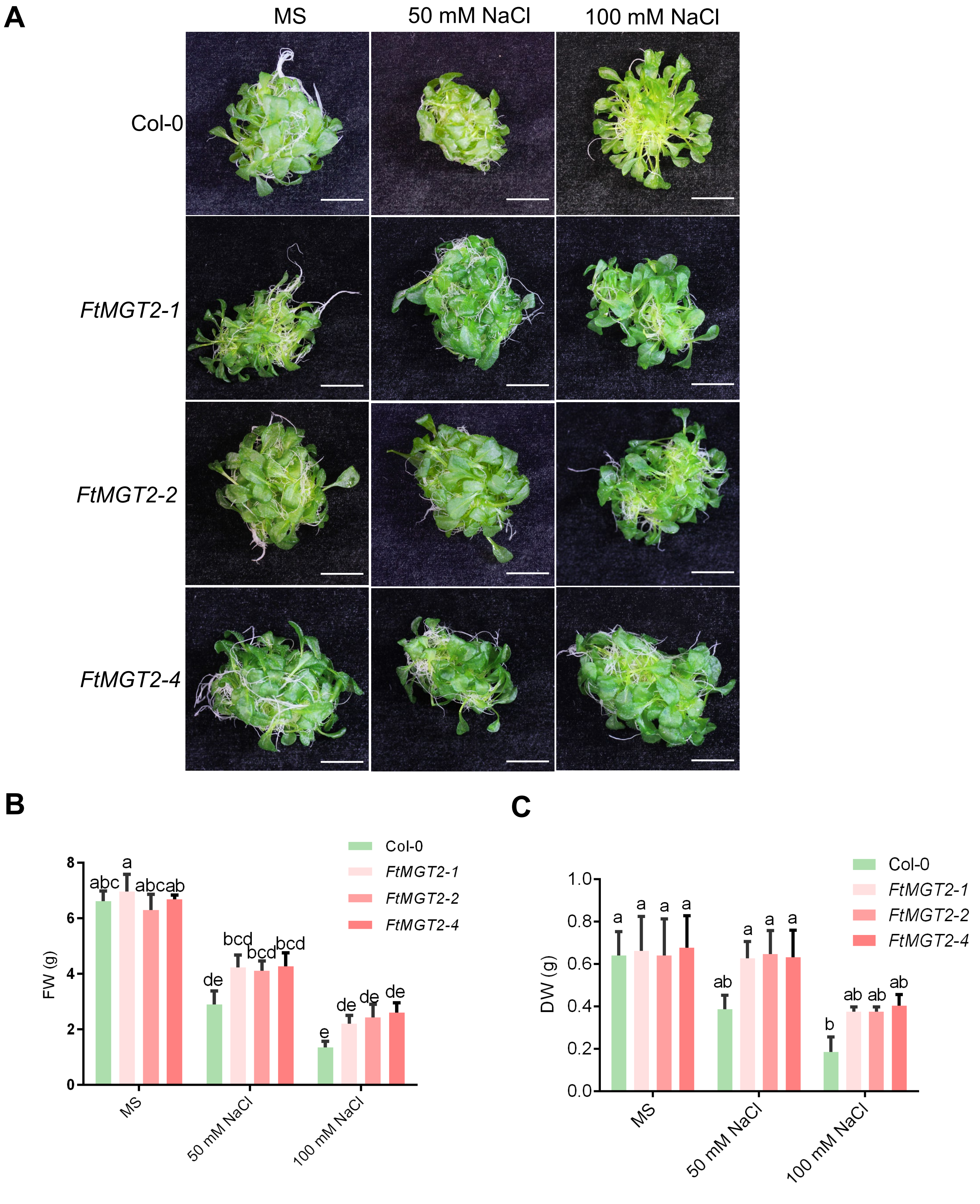

Figure S14 *FtMGT2* overexpression *Arabidopsis* can enhance plant salt tolerance. A) The phenotype of *FtMGT2* overexpression *Arabidopsis* treated with different salt concentrations. Bar = 5 mm. **B-C)** The fresh weight **(B)** and dry weight **(C)** of seedlings in **A**. Data in **B** and **C** are presented as the mean ± SD from n = 3 independent biological replicates. Statistical analysis was performed using one-way ANOVA analysis with Tukey's HSD test (Different letters represent significant differences at $P < 0.05$).

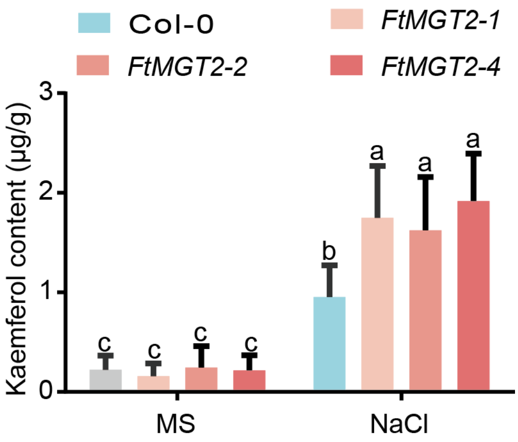

Figure S15 The salt standard treatment experiment demonstrated that the overexpression of *FtMGT2* confers increased salt tolerance. The content of kaempferol of Col-0 and *FtMGT2* heterologous expression *Arabidopsis* grown for 10 days in MS and MS solid medium containing 100 mM NaCl. Col-0, wild-type *Arabidopsis*, *FtMGT2-1*, *FtMGT2-2*, *FtMGT2-4*, three lines of *FtMGT2* heterologous expression *Arabidopsis*. Data are presented as the mean ± SD from n = 3 independent biological replicates. Statistical analysis was performed using one-way ANOVA analysis with Tukey's HSD test (Different letters represent significant differences at $P < 0.05$).

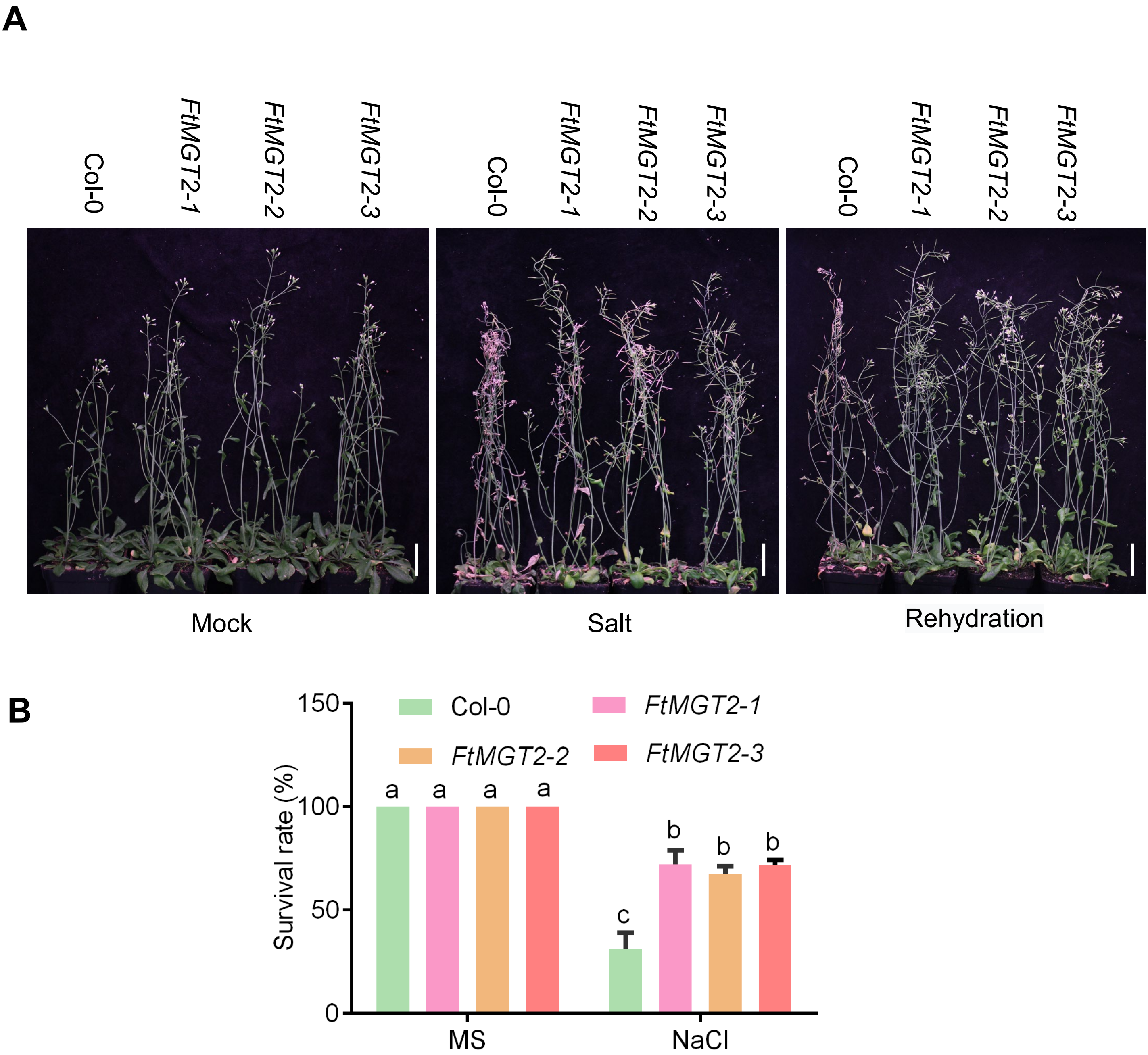

Figure S16 The salt standard treatment experiment demonstrated that the overexpression of *FtMGT2* confers increased salt tolerance. A) Mock, control treatment; Salt, treat *Arabidopsis* with a solution containing 100 mM NaCl for 2 weeks; Rehydration, rehydrate *Arabidopsis* for 2 weeks after 2 weeks of salt treatment. Bar = 5 cm. **B)** The survival rate of *Arabidopsis* in **A**. This study was conducted through three independent experiments, with six pots (four plants per pot) allocated to both the wild-type and transgenic lines in each independent experiment. Data from these parallel experiments were utilized to determine the rehydration rates. Data are presented as the mean ± SD from n = 3 independent biological replicates. Statistical analysis was performed using one-way ANOVA analysis with Tukey's HSD test (Different letters represent significant differences at $P < 0.05$).

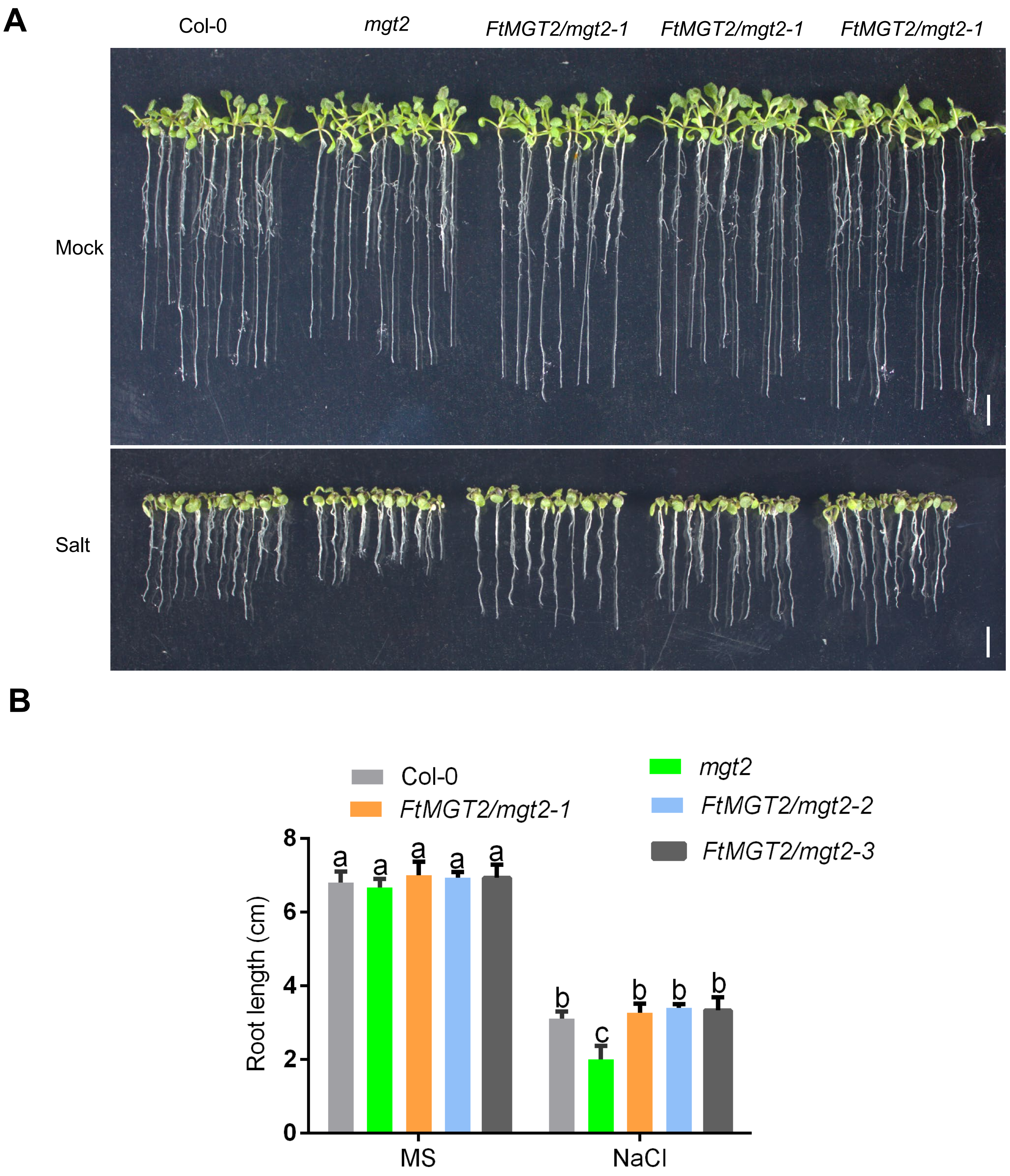

Figure S17 *FtMGT2* can mitigate the harm induced by salt stress in the *mgt2* mutant. A) Mock, control treatment; Salt, treat *Arabidopsis* with a solution containing 100 mM NaCl for 1 weeks. Bar = 5 mm. **B)** The root length of *Arabidopsis* in **A**. Data are presented as the mean ± SD from n = 3 independent biological replicates. Statistical analysis was performed using one-way ANOVA analysis with Tukey's HSD test (Different letters represent significant differences at $P < 0.05$).

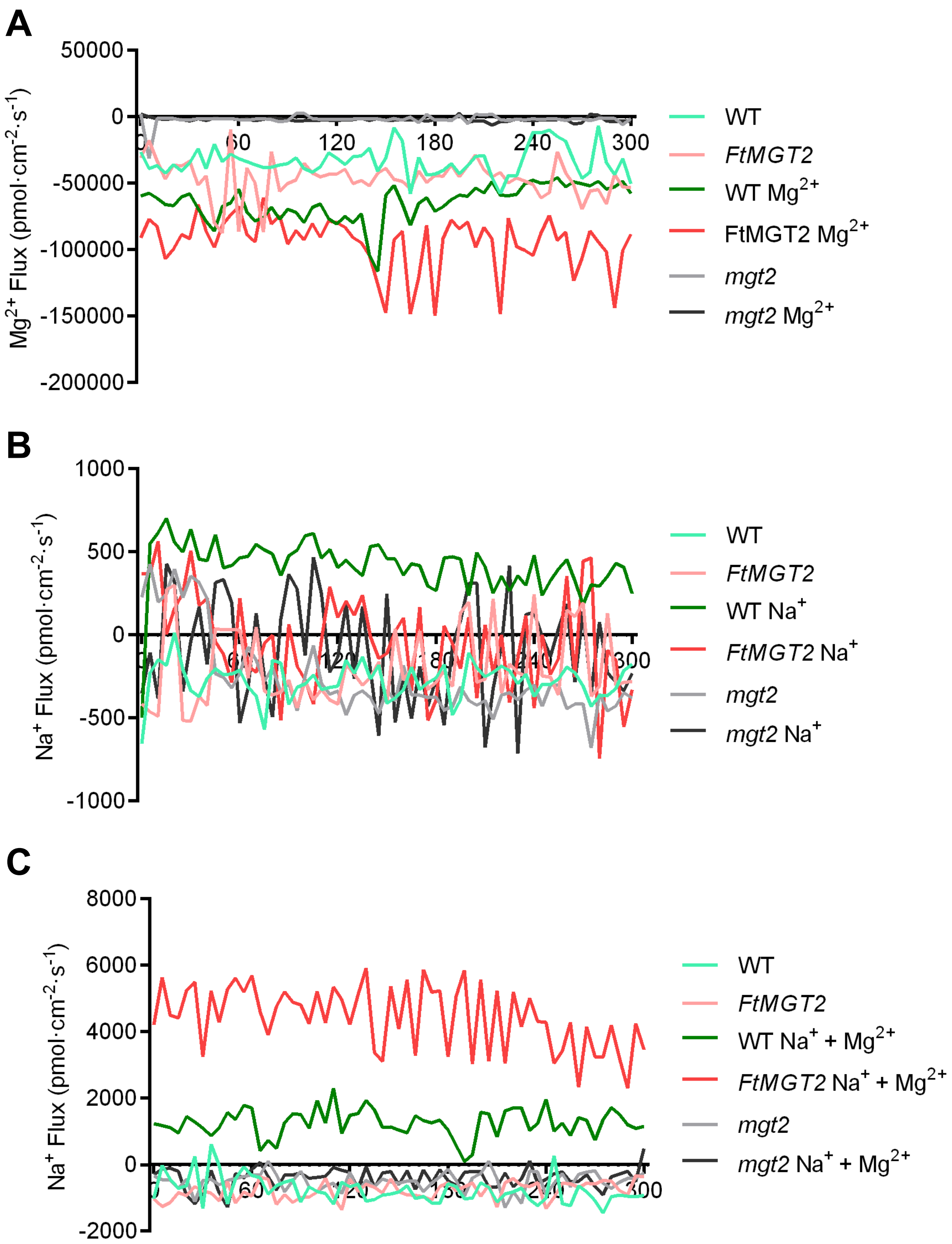

Figure S18 Dynamic changes of Mg^{2+} and Na^{+} in *FtMGT2* transgenic *Arabidopsis* under different ion treatment. A) The flow rates of Na^{+} in various *FtMGT2* transgenic *Arabidopsis* materials under Mg^{2+} treatment. **B)** The flow rates of Na^{+} in various *FtMGT2* transgenic *Arabidopsis* materials under Na^{+} treatment. **C)** The flow rates of Na^{+} in various *FtMGT2* transgenic *Arabidopsis* materials under Na^{+} + Mg^{2+} treatment. Positive values indicate efflux, whereas negative values signify absorption.

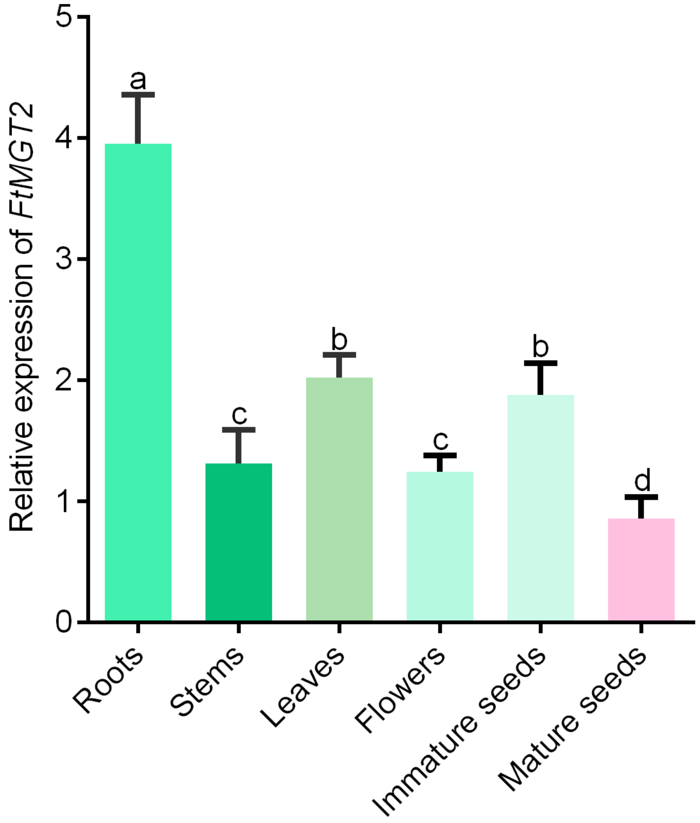

Figure S19 Expression levels of *FtMGT2* in different tissues of Tartary buckwheat seedlings. Data are presented as the mean ± SD from n = 3 independent biological replicates. Statistical analysis was performed using one-way ANOVA analysis with Tukey's HSD test (Different letters represent significant differences at $P < 0.05$).

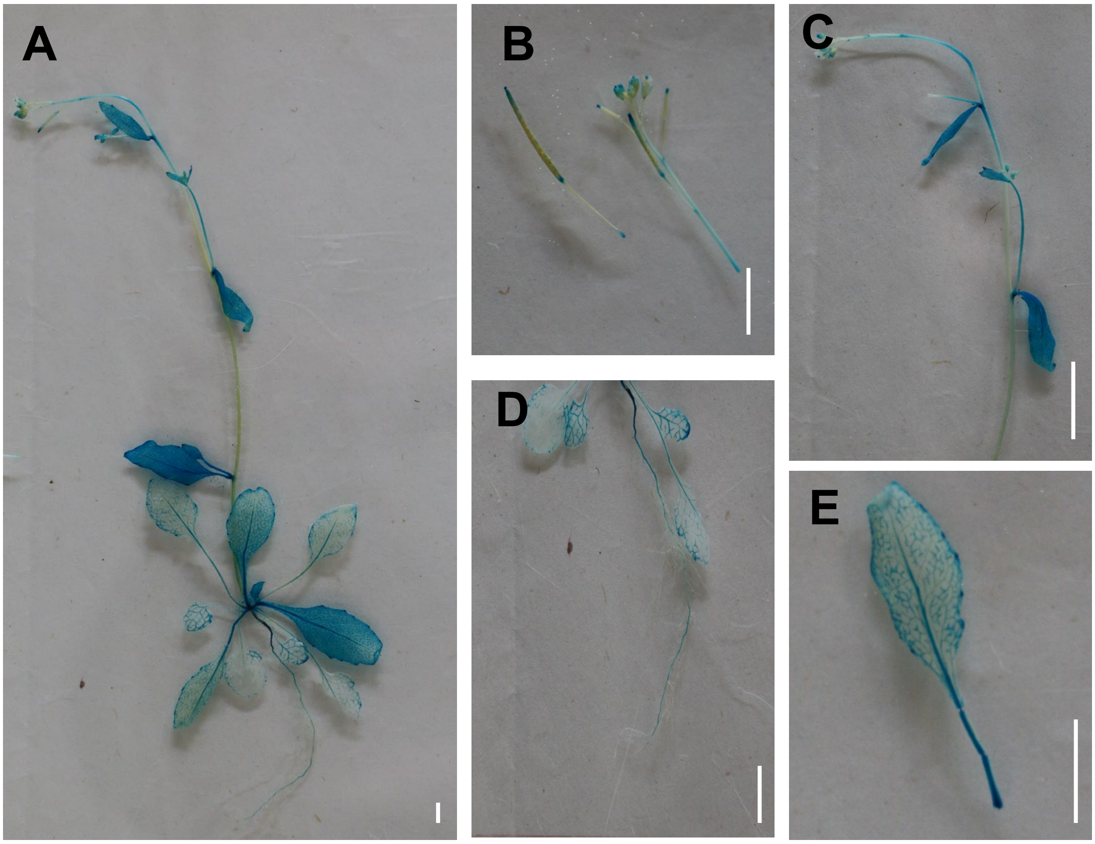

Figure S20 The *FtMGT2* promoter exhibits high expression in both roots and mature leaves. The GUS staining results of the *FtMGT2* promoter-GUS transgenic *Arabidopsis*: **A)** Whole plants, B**)** Flowers and pods, **C)** Stems, **D)** Roots, **E)** Leaves. Bar = 1 cm.

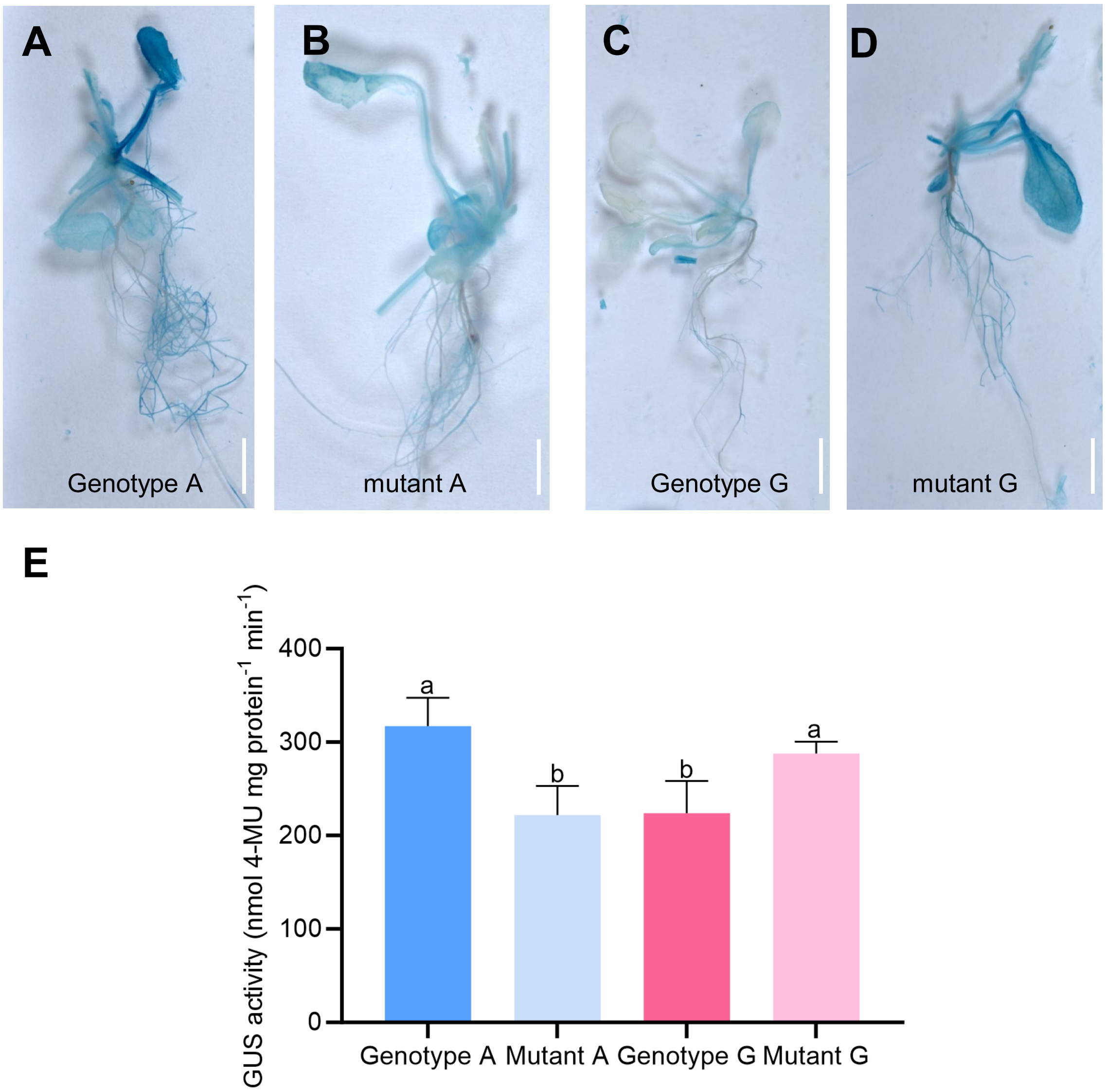

Figure S21 The *FtMGT2* promoter of different genotypes exhibits different expression in both roots and leaves. A) The GUS staining results of the *FtMGT2* promoter of genotype A GUS transgenic *Arabidopsis*. **B)** The GUS staining results of the *FtMGT2* promoter of mutant A (a point mutation in the genotype A promoter sequence transforms it into that of genotype G) GUS transgenic *Arabidopsis*. **C)** The GUS staining results of the *FtMGT2* promoter of genotype G GUS transgenic *Arabidopsis*. **D)** The GUS staining results of the *FtMGT2* promoter of mutant G (a point mutation in the genotype G promoter sequence transforms it into that of genotype A GUS transgenic *Arabidopsis*. Bar = 1 cm. **E)** The GUS activity of different GUS transgenic *Arabidopsis*. Data are presented as the mean ± SD from n = 3 independent biological replicates. Statistical analysis was performed using one-way ANOVA analysis with Tukey's HSD test (Different letters represent significant differences at $P < 0.05$).

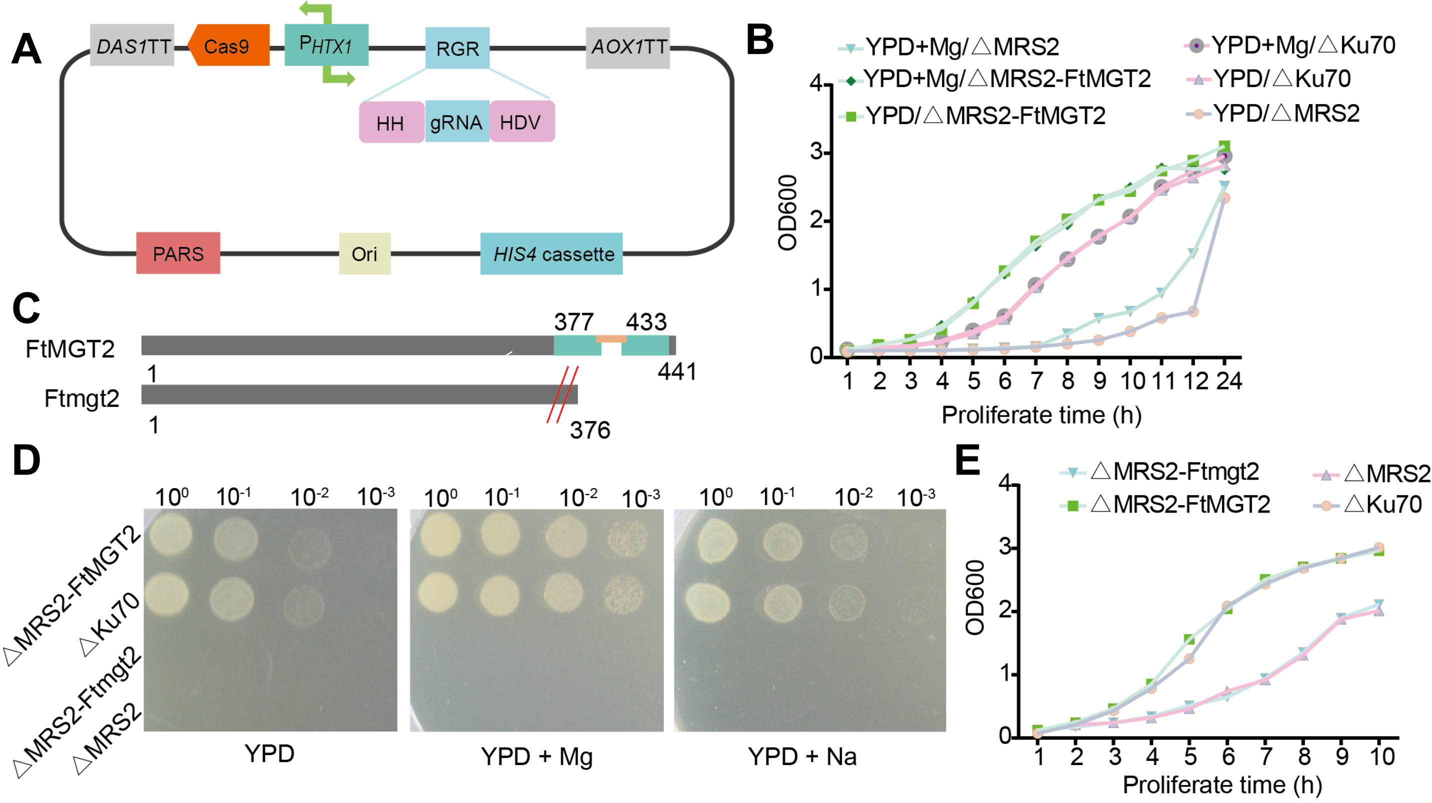

Figure S22 Phenotype of different yeast strains in different YPD solid medium. △Ku70, a wild-type yeast strain; △ MRS2, mutant yeast strain; △ MRS2-MGT2, cover yeast strain. Bar = 2 cm. **A)** The schematic diagram of yeast knockout vector construction. **B)** The growth curves of three yeast strains in YPD and YPD + Mg^{2+} solid medium, as shown in B, with OD600 values on the vertical axis and proliferation time on the horizontal axis. **C)** The schematic diagram depicts the location of the transmembrane domain in *FtMGT2* and the structural alteration of the mgt2 mutant after truncating the transmembrane structure. **D)** The growth of four yeast strains in solid medium of YPD, YPD + 100 mM Mg^{2+} and 100 mM YPD + Na^{+}. *△MRS2-Ftmgt2*, a complementary yeast strain. **E)** The growth curves of four yeast strains in YPD + Mg^{2+} liquid medium.

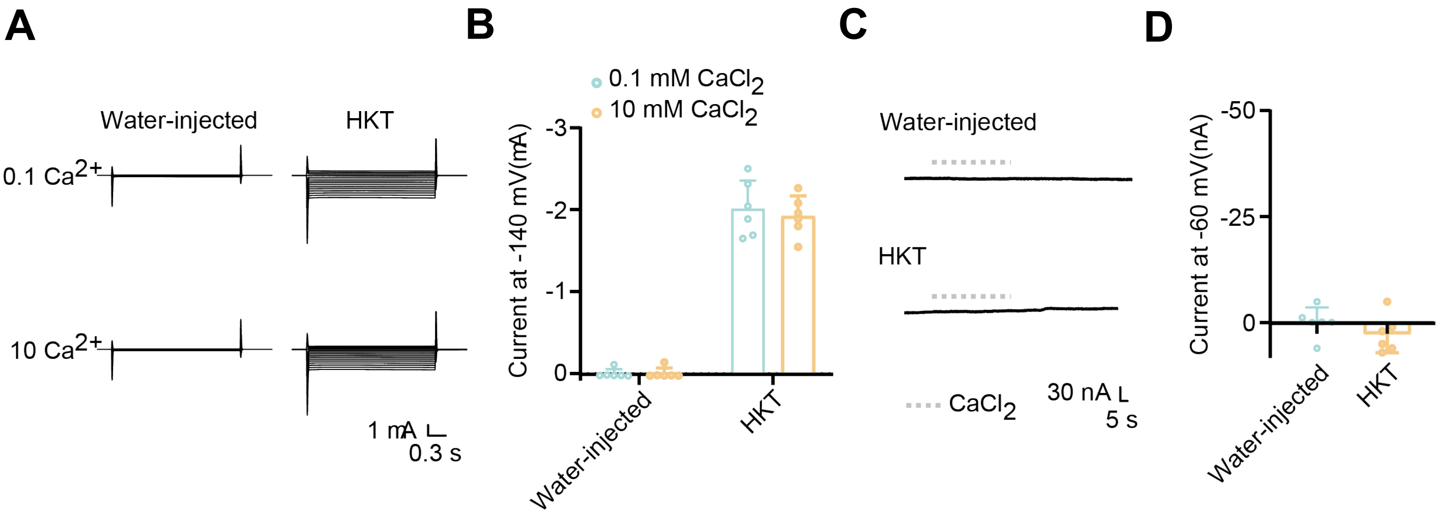

Figure S23 TEVC experiments were performed using *X. laevis oocytes* injected with water, *FtMGT2* cRNA or *FtHKT1* cRNA in the presence of different concentrations of Ca^{2+}. A) A current profile of water-injected or *FtHKT1* oocytes, bathed in different concentrations of Ca^{2+}. **B)** Current-voltage relationships of oocytes injected with water or *FtHKT1* cRNA. **C-D)** TEVC of water-injected or *FtHKT1 Xenopus laevis oocytes* showing no effect of Ca (100 µM) on endogenous currents membrane potential (EM) Data in **B** and **D** are presented as the mean ± SD from n = 3 independent biological replicates. Statistical significance was determined using a two-sided Student's *t*-test. Asterisks indicate significant differences.

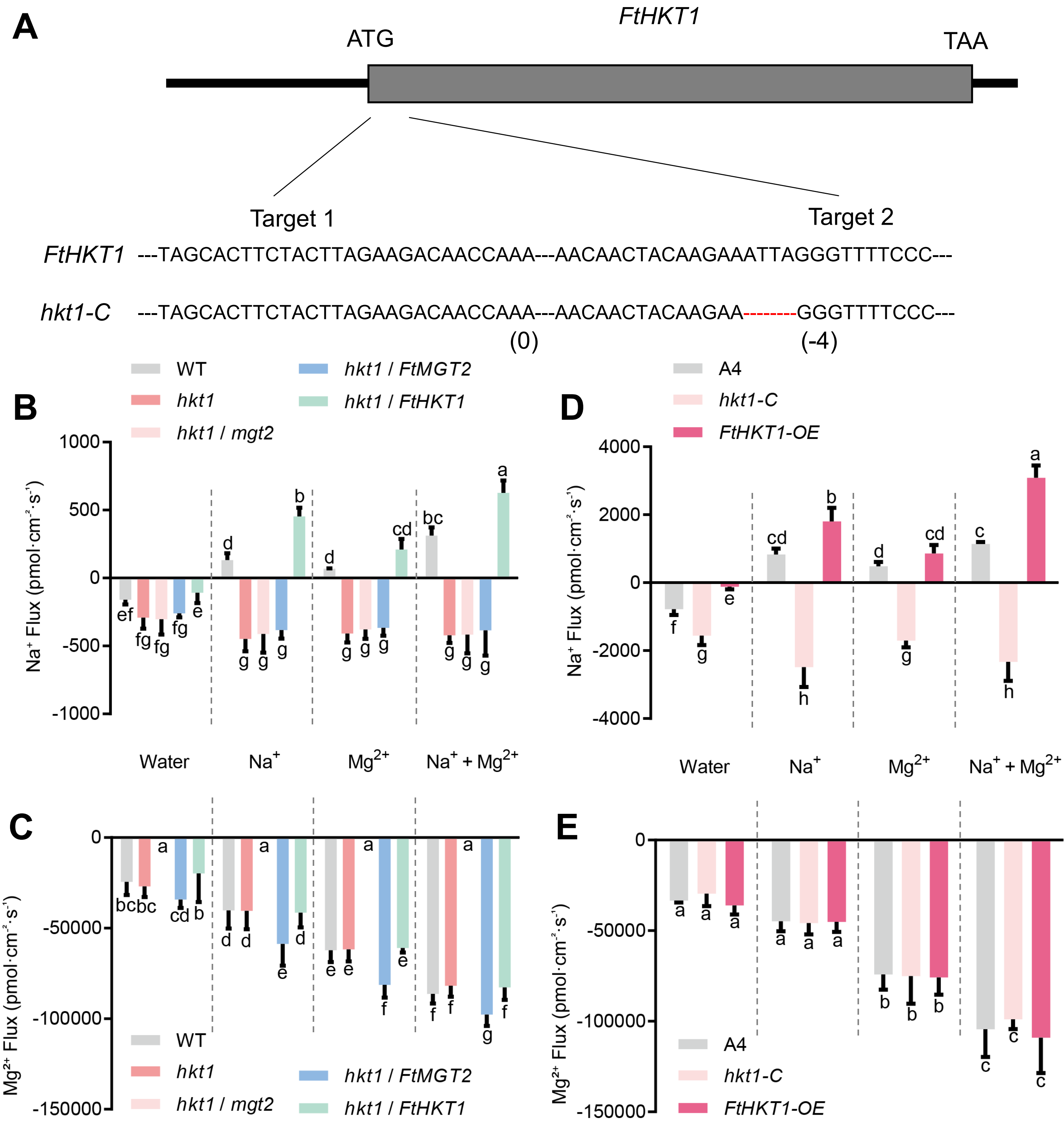

Figure S24 Steady-state Na$^+$ and Mg^{2+} fluxes of *FtHKT1* transgenic plants under different treatment at the final peaks were calculated. A) Sequencing results of the mutant hairy roots show that the *FtHKT1* has been successfully knocked out. *FtHKT1,* reference sequence for target site. *hkt1-C*, sequence of the target site in *hkt1-C* mutant hairy roots. **B)** The flow rates of Na$^+$ in various *FtHKT1* transgenic *Arabidopsis* materials under different treatment. **C)** The flow rates of Mg^{2+} in various *FtHKT1* transgenic *Arabidopsis* materials under different treatment. **D)** The flow rates of Na$^+$ in various *FtHKT1* transgenic Tartary buckwheat hairy roots materials under different treatment. **E)** The flow rates of Mg^{2+} in various *FtHKT1* transgenic Tartary buckwheat hairy roots materials under different treatment. Positive values indicate efflux, whereas negative values signify absorption. Data in **B**, **C**, **D** and **E** are presented as the mean ± SD from n = 3 independent biological replicates. Statistical analysis was performed using one-way ANOVA analysis with Tukey's HSD test (Different letters represent significant differences at $P < 0.05$).

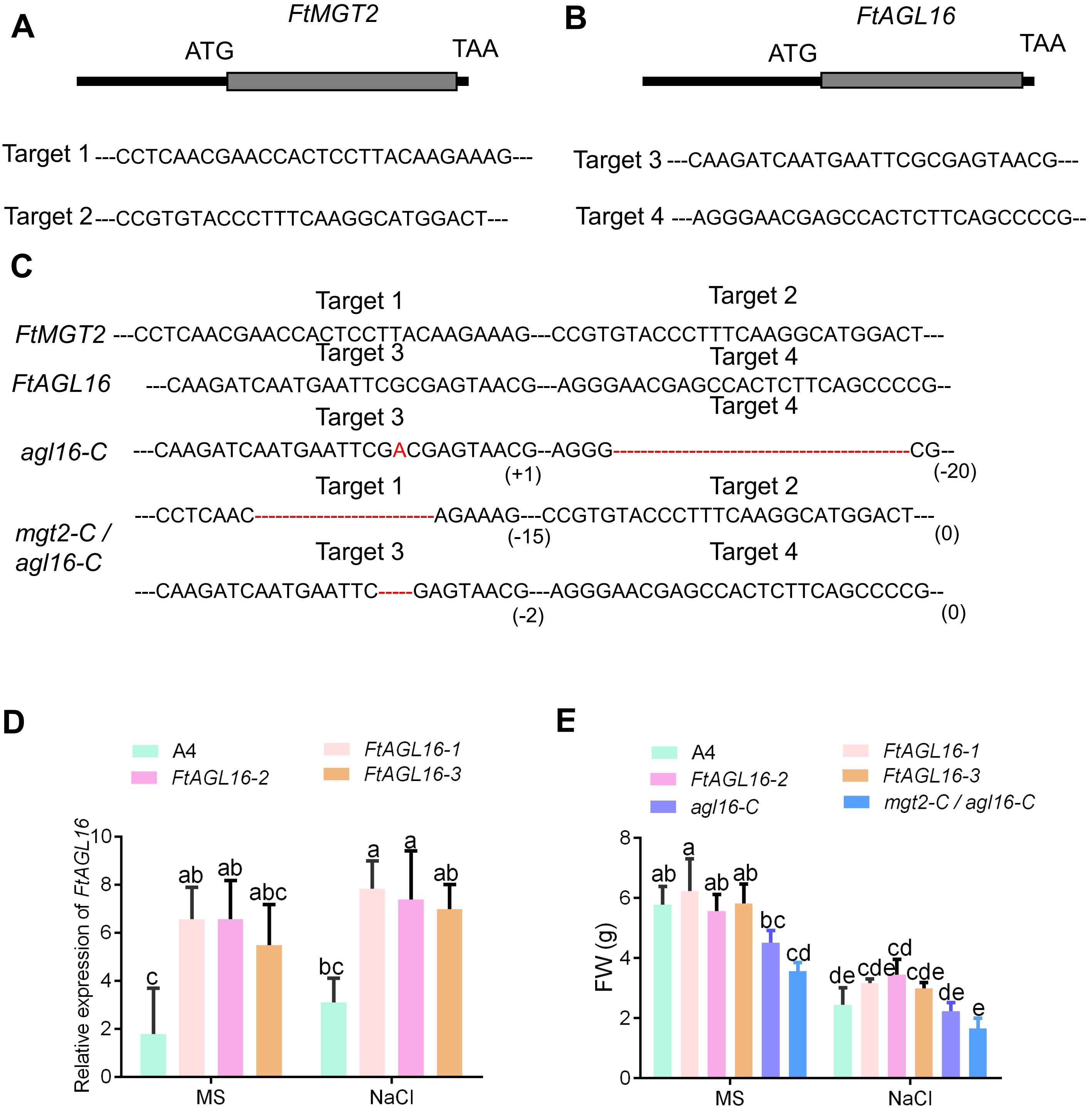

Figure S25 Sequencing results of the mutant hairy roots show that the *FtAGL16* has been successfully knocked out. A-B) *FtMGT2* and *FtAGL16* reference sequence for target site. **C)** *agl16-C* or *mgt2-C / agl16-C*, sequence of the target site in *agl16-C* and *mgt2-C / agl16-C* mutant hairy roots. **D)** Relative expression of *FtAGL16* in *FtAGL16* OE hairy roots and knockout hairy roots. **E)** Fresh weight of *FtAGL16* OE hairy roots and knockout hairy roots. Data in **D** and **E** are presented as the mean ± SD from n = 3 independent biological replicates. Statistical analysis was performed using one-way ANOVA analysis with Tukey's HSD test (Different letters represent significant differences at $P < 0.05$).

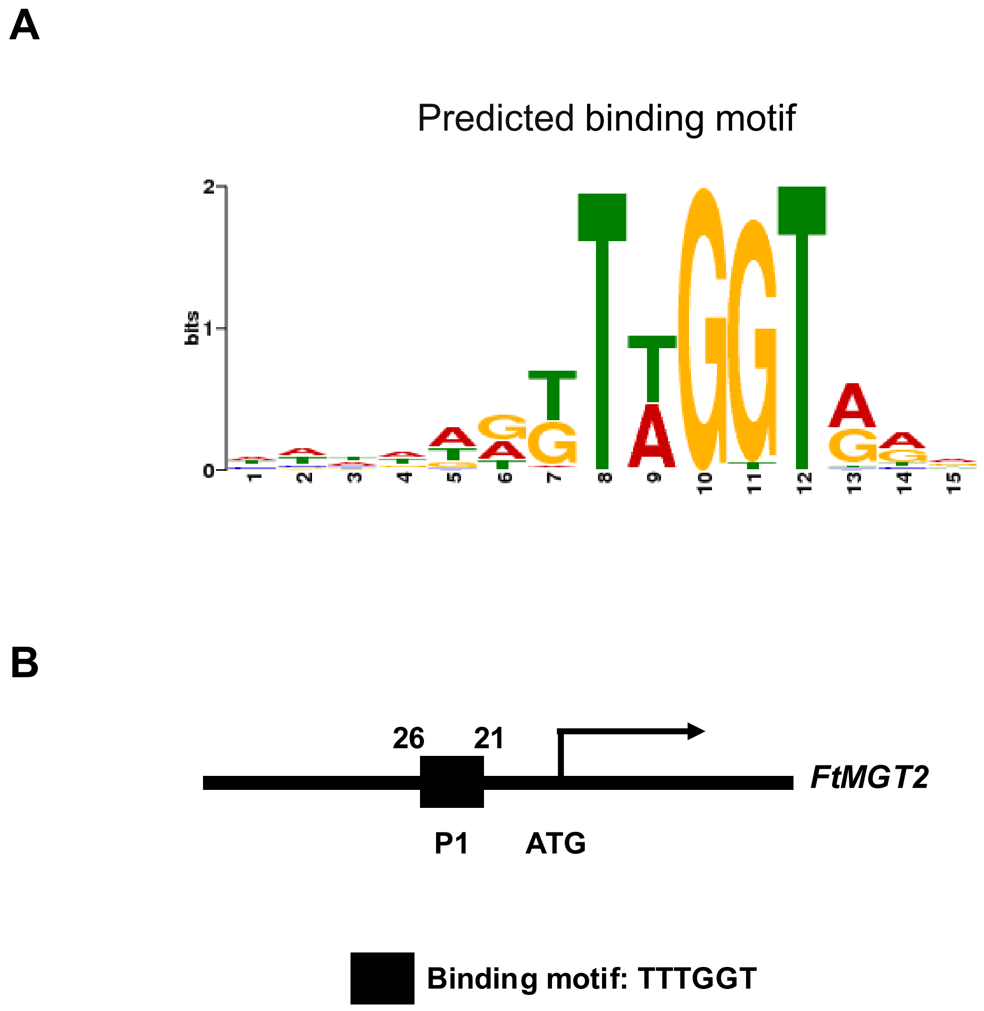

Figure S26 The sequence (A) and position (B) of predicted FtMYB15L binding motif on *FtMGT2* promoter.

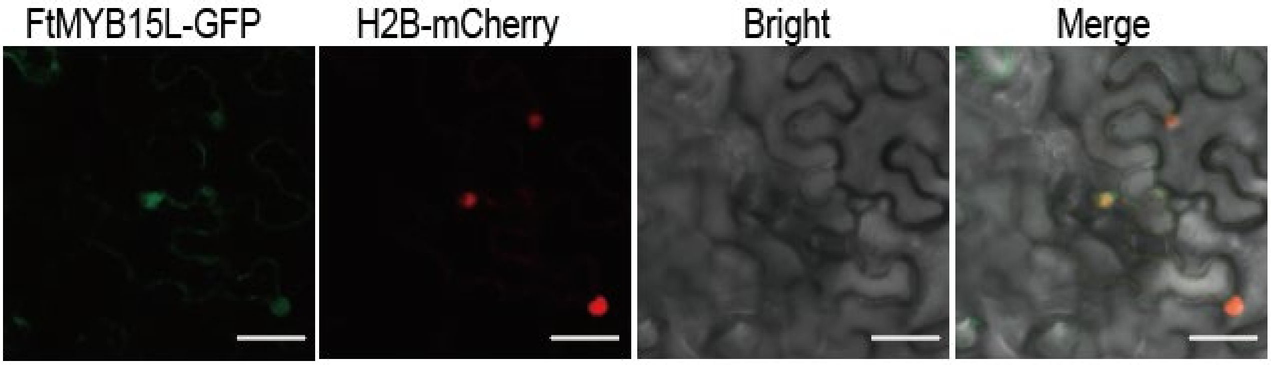

Figure S27 The subcellular localization of FtMYB15L. FtMYB15L-GFP, pCAMBIA1300-FtMYB15L-GFP materials channel; H2B-mCherry, nuclear marker; Bright, bright channel; Merge, merge channel. Bar = 10 μm.

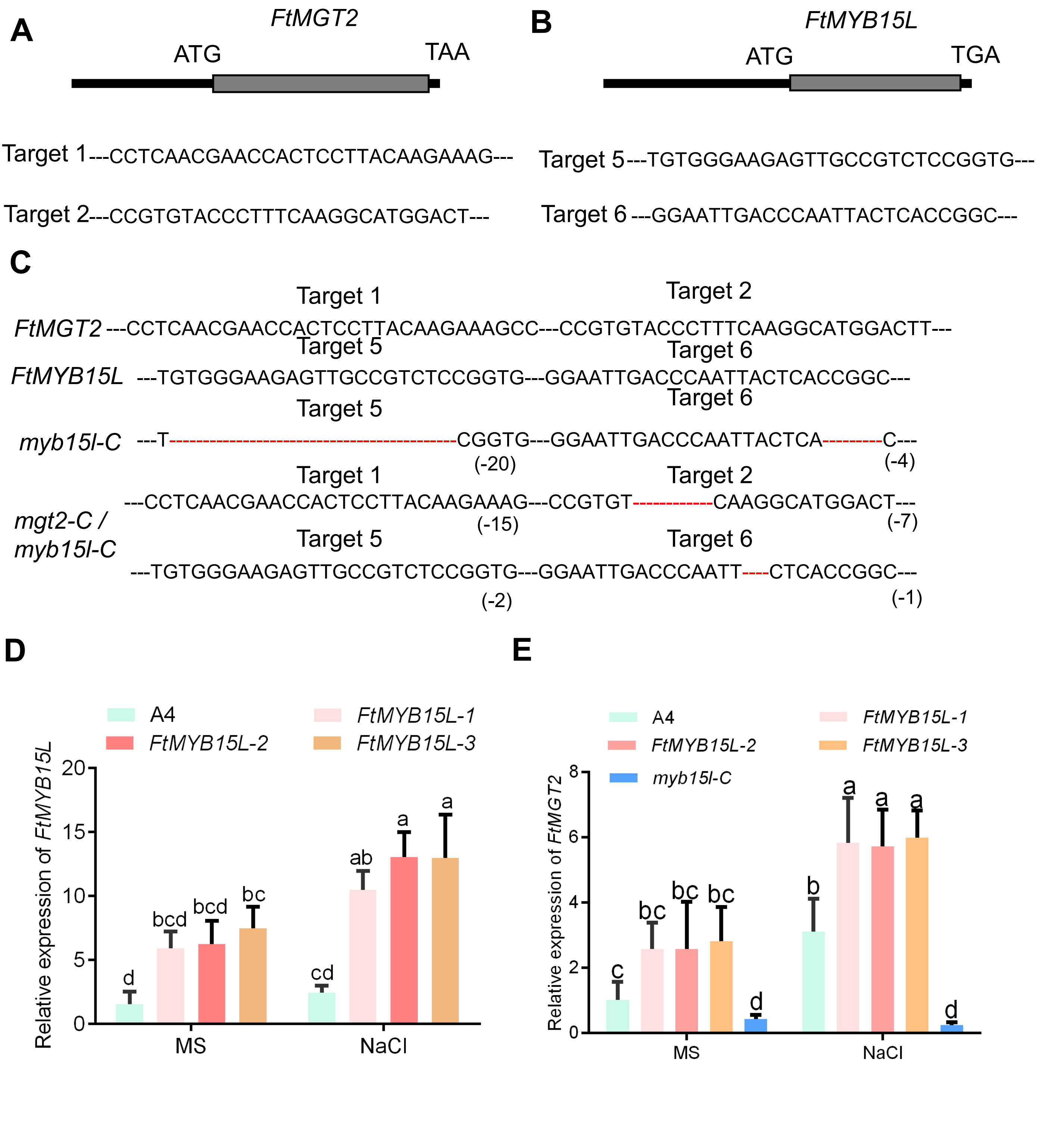

Figure S28 Sequencing results of the mutant hairy roots show that the *FtMYB15L* has been successfully knocked out. A-B) *FtMGT2* and *FtMYB15L* reference sequence for target site. **C)** *myb15l-C* or *mgt2-C / myb15l-C*, sequence of the target site in *myb15l-C* and *mgt2-C / myb15l-C* mutant hairy roots. **D)** Relative expression of *FtMYB15L* in *FtMYB15L* OE hairy roots and knockout hairy roots. **E)** Relative expression of *FtMGT2* in *FtMYB15L* OE hairy roots and knockout hairy roots. Data in **D** and **E** are presented as the mean ± SD from n = 3 independent biological replicates. Statistical analysis was performed using one-way ANOVA analysis with Tukey's HSD test (Different letters represent significant differences at $P < 0.05$).

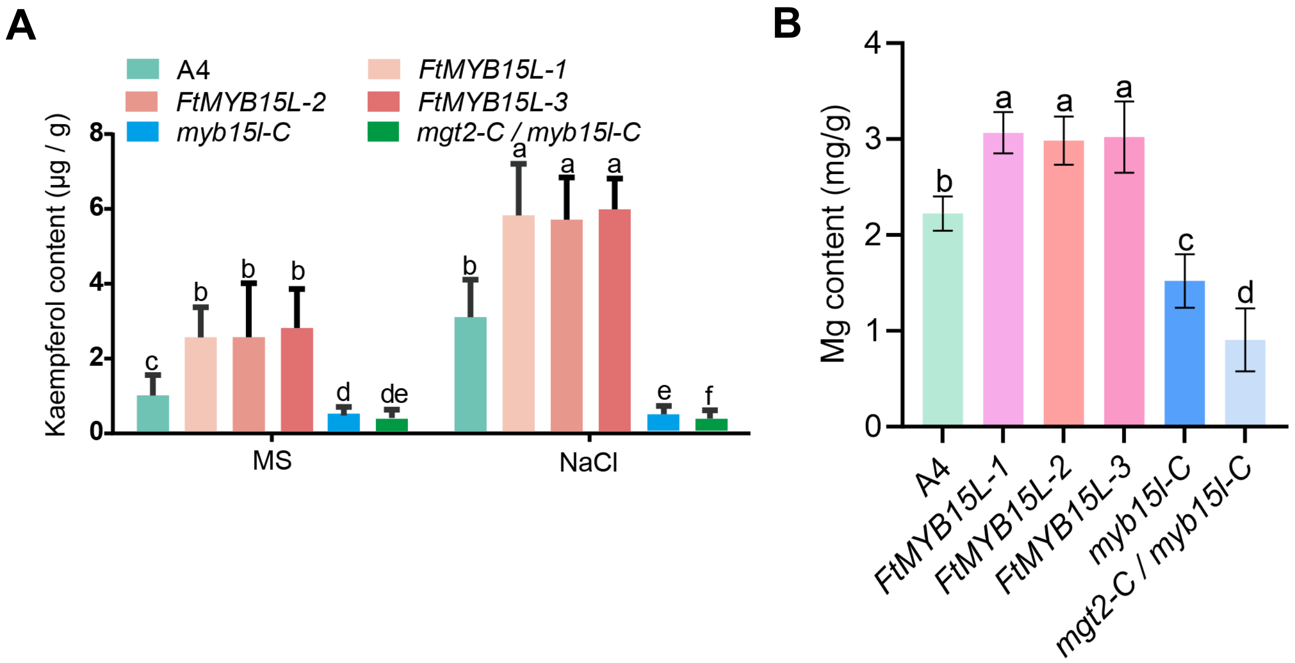

Figure S29 The kaempferol and Mg content of A4 hairy roots, *FtMYB15L* overexpressed and knockout hairy roots. **A)** The kaempferol content of different hairy roots. **B)** The Mg^{2+} content of different hairy roots. A4, A4 *Agrobacterium rhizogenes* empty strain hairy roots; *FtMYB15L-1*, *FtMYB15L-2*, and *FtMYB15L-3*, three *FtMYB15L* overexpressed hairy root strains, *myb15-C*, *FtMYB15L* knockout hairy roots; *mgt2-C / myb15l-C*, double mutant hairy roots of *FtMGT2* and *FtMYB15L*. Data in **A** and **B** are presented as the mean ± SD from n = 3 independent biological replicates. Statistical analysis was performed using one-way ANOVA analysis with Tukey’ s HSD test (Different letters represent significant differences at $P < 0.05$).

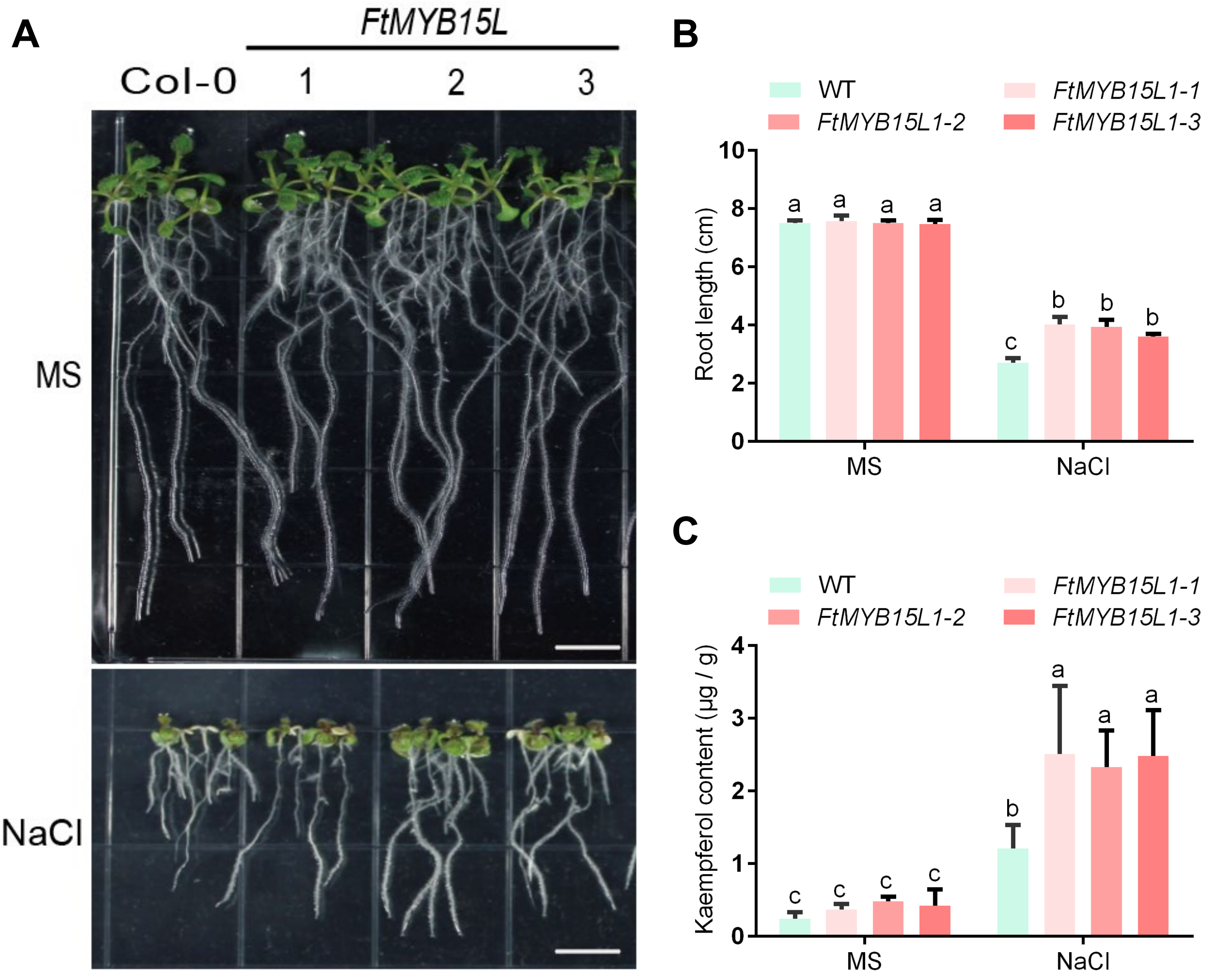

Figure S30 The phenotype of Col-0 and *FtMYB15L* overexpressed *Arabidopsis thaliana* grown for 10 days in MS solid medium and MS solid medium + 100 mM NaCl. A) Col-0, wild-type *Arabidopsis*, *FtMYB5L-1*, *FtMYB15L-2*, *FtMYB15L-3*, three lines of *FtMYB15L* heterologous expression *Arabidopsis*. Bar = 5 mm. **B)** The root length (cm) under **A** condition. **C)** The content of kaempferol under **A** condition. Data in **B** and **C** are presented as the mean ± SD from n = 3 independent biological replicates. Statistical analysis was performed using one-way ANOVA analysis with Tukey's HSD test (Different letters represent significant differences at $P < 0.05$).

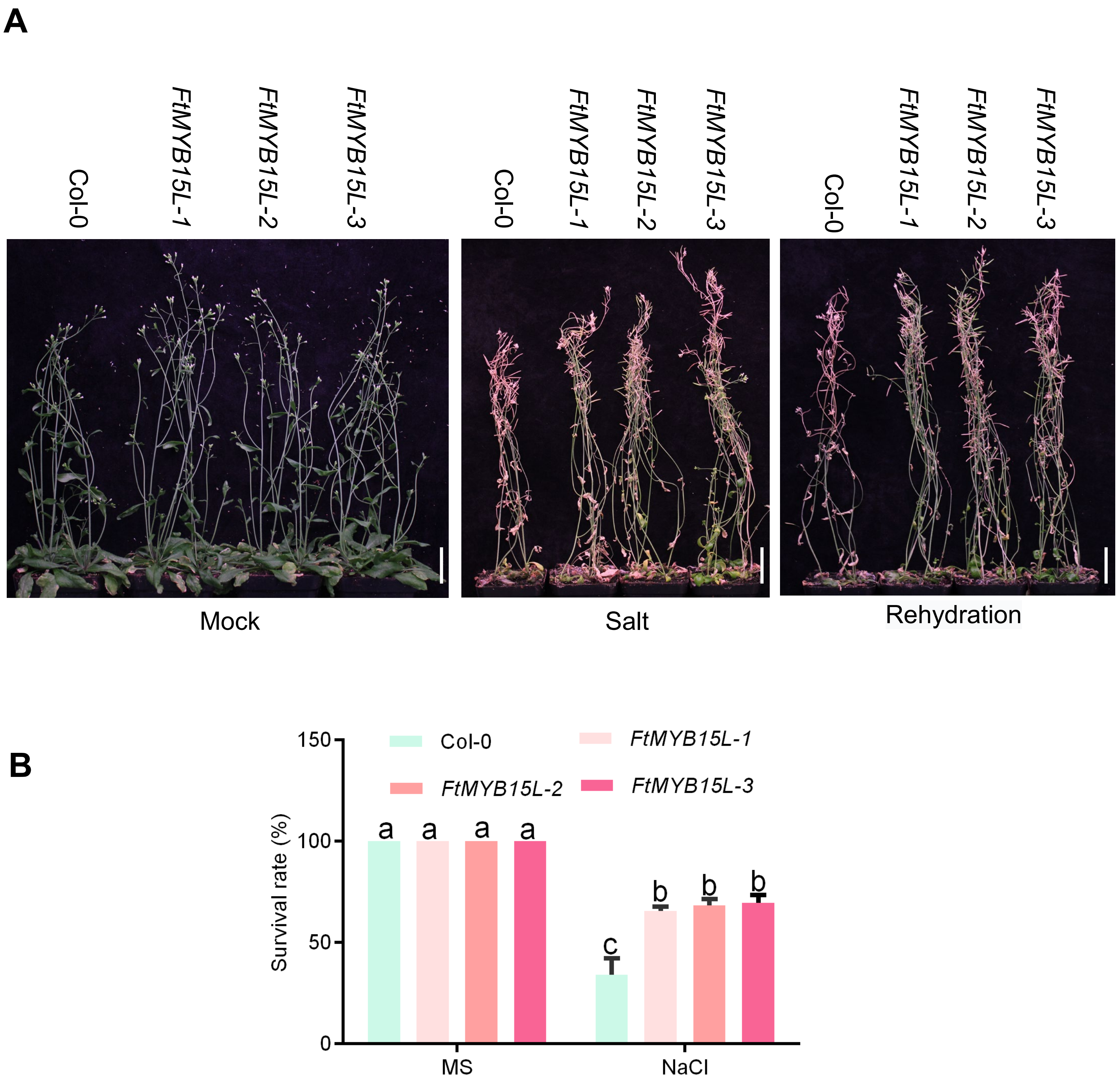

Figure S31 The salt standard treatment experiment demonstrated that the overexpression of *FtMYB15L* confers increased salt tolerance. A) Mock, control treatment; Salt, treat *Arabidopsis* with a solution containing 100 mM NaCl for 2 weeks; Rehydration, rehydrate *Arabidopsis* for 2 weeks after 2 weeks of salt treatment. Bar = 5 cm. **B)** The survival rate of *Arabidopsis* in **A**. This study was conducted through three independent experiments, with six pots (four plants per pot) allocated to both the wild-type and transgenic lines in each independent experiment. Data from these parallel experiments were utilized to determine the rehydration rates. Data are presented as the mean ± SD from n = 3 independent biological replicates. Statistical analysis was performed using one-way ANOVA analysis with Tukey's HSD test (Different letters represent significant differences at $P < 0.05$).

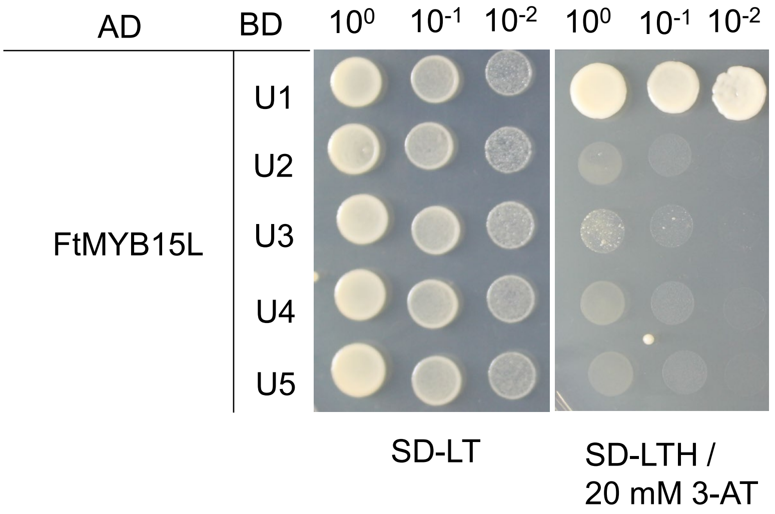

Figure S32 Yeast two hybridization identified candidate E3 ligase interacted with FtMYB15L. U1, FtPinG0606089000.01, FtBRG1; U2, FtPinG0100616700.01； U3, FtPinG0606392400.01; U4, FtPinG0404214400.01; U5, FtPinG0302633400.01.

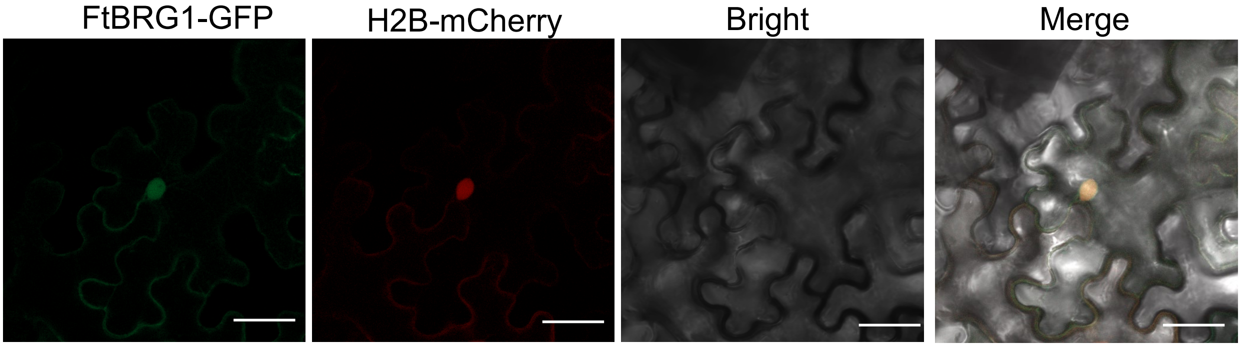

Figure S33 The subcellular localization of FtBRG1. FtBRG1-GFP, pCAMBIA1300-FtBRG1 recombination plasmid; H2B-mCherry, nuclear marker; Bright, bright channel; Merge, merge channel. Bar = 10 μm.

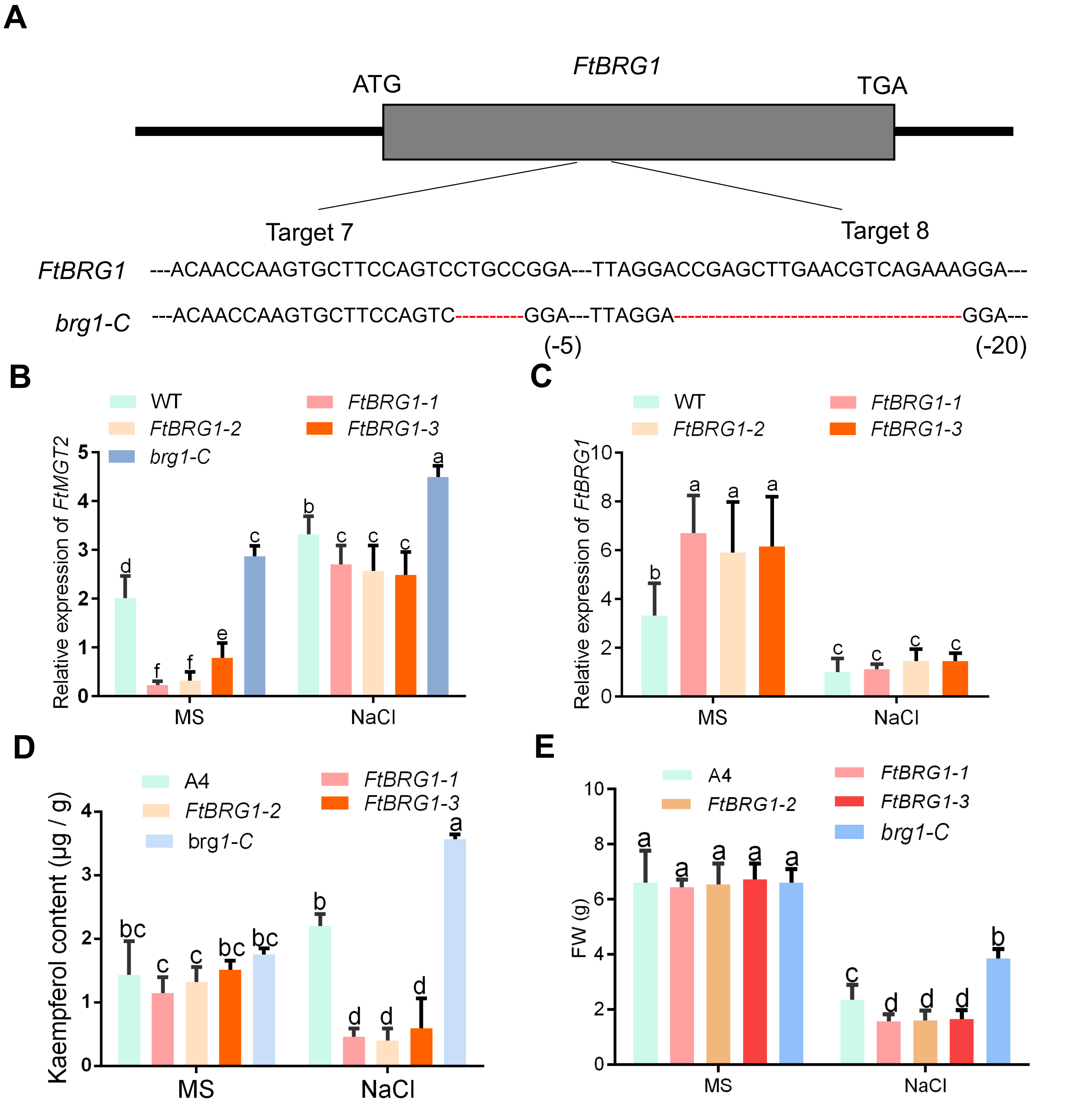

Figure S34 Sequencing results of the mutant hairy roots show that the *FtBRG1* has been successfully knocked out. A) *FtBRG1*, *FtBRG1* reference sequence for target site. *brg1-C*, sequence of the *FtBRG1* target site in *brg1-C* mutant hairy roots. **B)** Relative expression of *FtBRG1* in *FtBRG1* OE hairy roots and knockout hairy roots. **C)** Relative expression of *FtMGT2* in *FtBRG1* OE hairy roots and knockout hairy roots. **D)** The fresh weight was observed in A4 hairy roots, *FtBRG1* overexpressed and knockout hairy roots both under normal conditions and after exposure to 100 mM NaCl salt stress for 2 weeks, A4, A4 *Agrobacterium rhizogenes* empty strain hairy roots; *FtBRG1-1*, *FtBRG1-2*, and *FtBRG1-3*, three *FtBRG1* overexpressed hairy root strains. **E)** The kaempferol content of A4 hairy roots, *FtBRG1* overexpressed and knockout hairy roots. Data in **B**, **C**, **D** and **E** are presented as the mean ± SD from n = 3 independent biological replicates. Statistical analysis was performed using one-way ANOVA analysis with Tukey's HSD test (Different letters represent significant differences at $P < 0.05$).

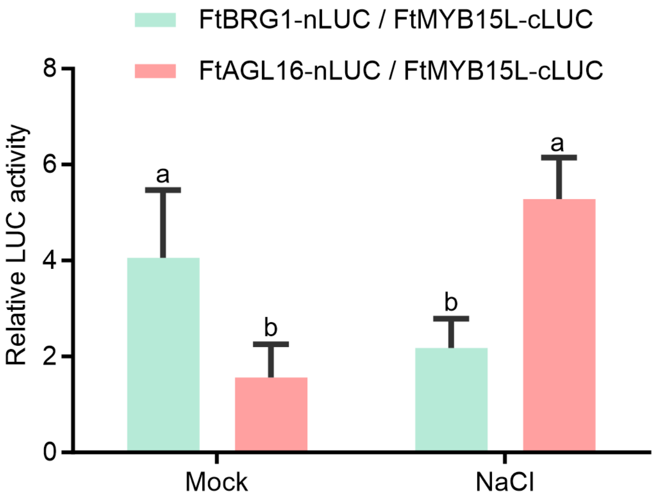

Figure S35 Relative LUC activity under Figure 7G. Mock, under normal condition. NaCl, under 100 mM NaCl condition. Data are presented as the mean ± SD from n = 3 independent biological replicates. Statistical analysis was performed using one-way ANOVA analysis with Tukey's HSD test (Different letters represent significant differences at $P < 0.05$).

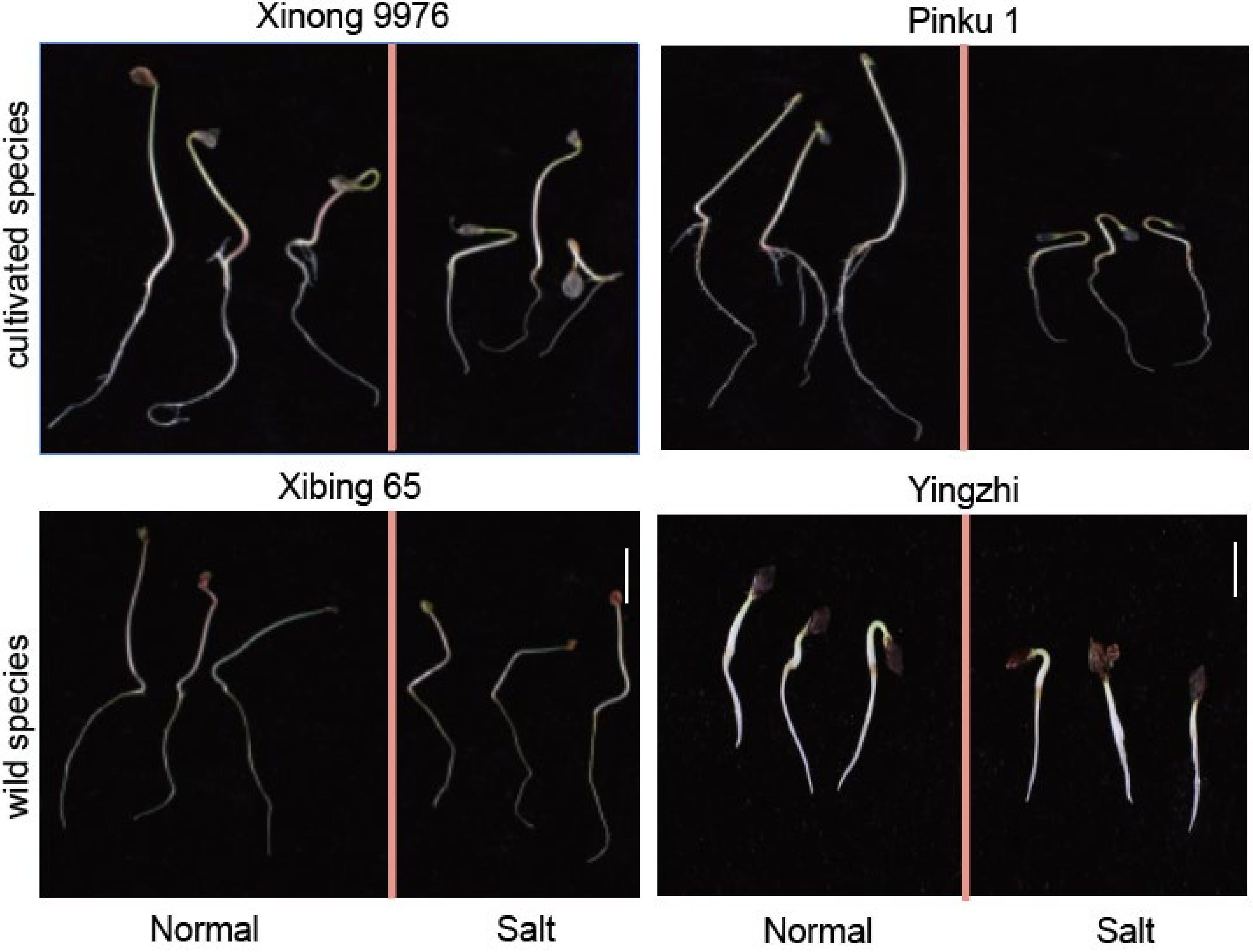

Figure S36 Phenotypes of cultivated species and wild species under salt stress. Two cultivated species including *F. tataricum* 'Pinku 1' and *F. esculentum* 'Xinong9976', two wild species including *F. gracilipes* 'Xibing 65' and *F. urophyllum* 'Yingzhi'. Normal, under water condition. Salt, under 100 mM NaCl stress. Bar = 1 cm.

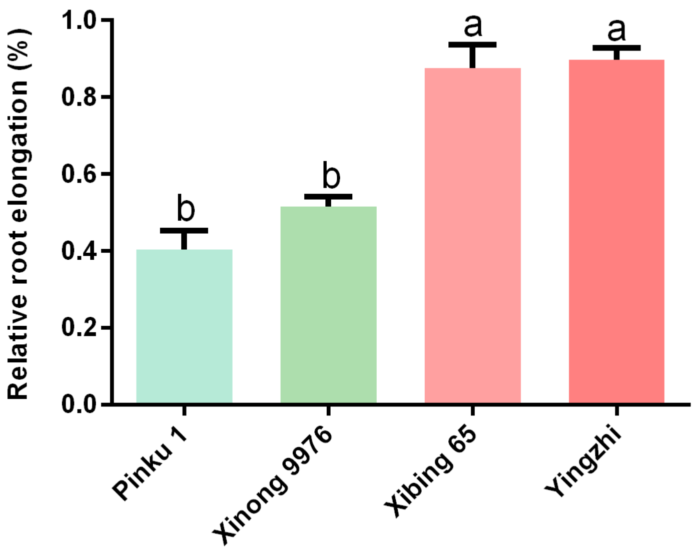

Figure S37 The relative root elongation of cultivated species and wild species under salt stress. Two cultivated species including *F. tataricum* 'Pinku 1' and *F. esculentum* 'Xinong9976', two wild species including *F. gracilipes* 'Xibing 65' and *F. urophyllum* 'Yingzhi'. The relative root elongation of four species in **Figure S36.** Data are presented as the mean ± SD from n = 3 independent biological replicates. Statistical analysis was performed using one-way ANOVA analysis with Tukey's HSD test (Different letters represent significant differences at $P < 0.05$).

```
FtMGT2-2kup       tgaaaatatccttttttttctctgt----tttggaggcttgtttcttgtttactaaaactt
FeMGT2-2kup       tgagaatatcctttttttctctctctgttttggaggattgtttcttgtttactggaacct
FgBMGT2-2kup      ---------ttttttttacattgt----tacg----------------------------
FuMGT2-2kup       tgagaacatctttttttacattgt----tacg----------------------------
                           .  ***** . .* *    * .*

FtMGT2-2kup       cattttcttagatgcttctatggttta---cttctttttatttttggttgtttt----tg
FeMGT2-2kup       ca-ttttttagatgcctctatggt------ttacttcta-tttttggtggtgtt----tg
FgBMGT2-2kup      -acttacactactgcatctttgattcaa--tctcttttttttcctgatgtttttattatg
FuMGT2-2kup       -acttatactgctgcatctttgattcaatctcttttttttttcctgatgtttttattatg
                   * ** . . . *.* *** **.*       . .**.*  **..*..*  * **    **

FtMGT2-2kup       gatttcctagta
FeMGT2-2kup       gatttcctagta
FgBMGT2-2kup      tacttcctagta
FuMGT2-2kup       tacttcctagta
                   *.*********
```

Figure S38 The difference sequence of *FtMGT2* promoter in cultivated species and wild species.

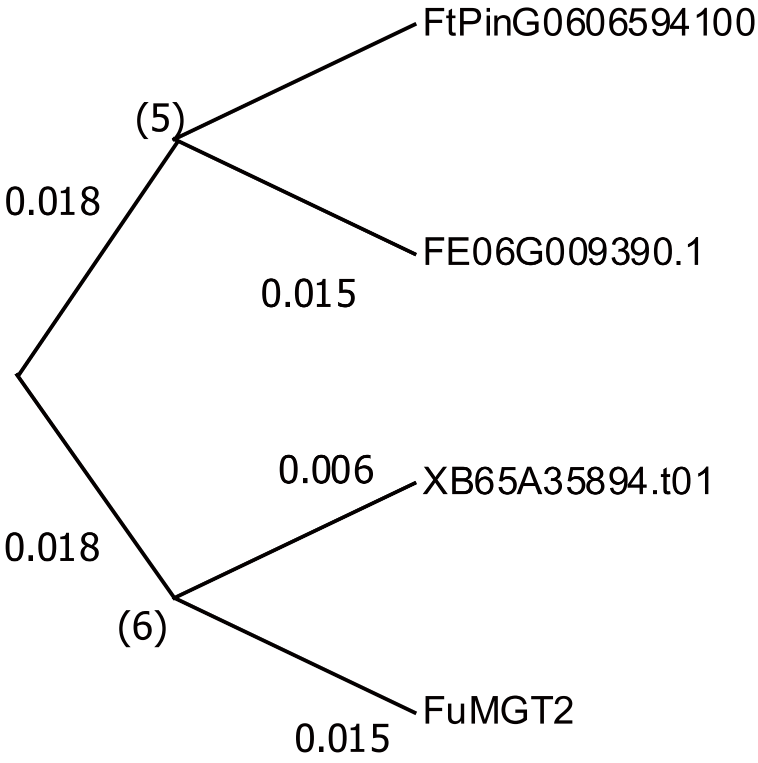

Figure S39 Phylogenetic analysis of FtMGT2 gene with four species.

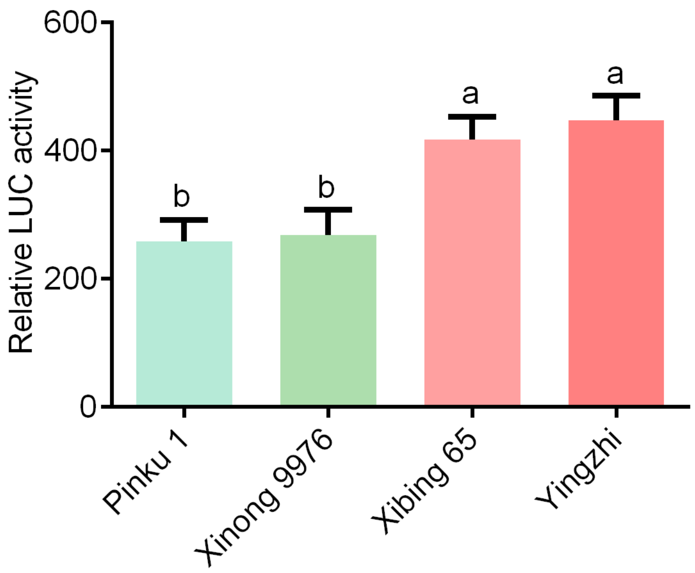

Figure S40 The relative LUC activity between four species. Two cultivated species including *F. tataricum* 'Pinku 1' and *F. esculentum* 'Xinong9976', two wild species including *F. gracilipes* 'Xibing 65' and *F. urophyllum* 'Yingzhi'. The relative LUC activity of *FtMGT2*pro-mini-LUC from four species. Data are presented as the mean ± SD from n = 3 independent biological replicates. Statistical analysis was performed using one-way ANOVA analysis with Tukey's HSD test (Different letters represent significant differences at $P < 0.05$).
