# Supplementary material for: Domestication of Tartary Buckwheat Shaped a Regulatory Module for Seedling Salt Tolerance by Targeting the Magnesium Transporter Gene FtMGT2
Source: Adv Sci (Weinh). 2025 Nov 25;13(8):e11570. doi: 10.1002/advs.202511570 (PMC12884793; doi:10.1002/advs.202511570)
Supplement: Supplementary file 4 — Supporting Information [file ADVS-13-e11570-s005.docx]

**Methods**

**Salt Tolerance Evaluation of Tartary Buckwheat Accessions**

To screen for salt tolerance, 220 Tartary buckwheat accessions were subjected to a germination assay under salt stress. For each accession, thirty seeds were placed on filter paper in Petri dishes and moistened with one of three NaCl solutions: 0% (control), 0.6% (100 mM), or 1.2% (200 mM). Ultrapure water was purchased from Beijing Jubinglong Chemical Reagent Co., Ltd. (Beijing, China). The experiment was conducted in triplicate for each treatment. After three days of incubation under controlled conditions, the epicotyl and radicle lengths were measured. To evaluate the effect of salt stress, several indices were calculated. First, the salt tolerance coefficient for the germination index (STC_GI_), radicle length (STC_RL_), and epicotyl length (STC_EL_) were calculated for each stress treatment relative to the control using the following formula:

STC(%)=(Value in control / Value under stress)×100

Subsequently, these coefficients were standardized using a membership function value, X(STC_i_), calculated for each parameter as:

X(STC_i_)= STC_max_−STC_min_ / STC_i_−STC_min_

Where STC_i_ is the measured value of a specific salt tolerance coefficient (i.e., STC_GI_, STC_RL_, or STC_EL_), and STC_min_ and STC_max_ are the minimum and maximum values for that coefficient observed across all 220 accessions, respectively. The total membership function value (∑X) for a given accession under each salt concentration was the sum of its individual membership function values:

∑X=X(STC_GI_)+X(STC_RL_)+X(STC_EL_)

Finally, an overall Salt tolerance index (STI) for each accession was computed by summing the total membership function values from both stress levels:

STI=(∑X)_0.6% NaCl_ + (∑X)_1.2% NaCl_

​**Genome-wide Association Study**

Based on the datasets in previous study^5^, a total of 1,094,530 SNPs were used for the association analysis on the salt-tolerance index. GWAS was performed using the factored spectrally transformed linear mixed models (FaST-LMM)^68^, and the significance threshold was set at 1×10^-5^ according to the effective SNPs number that was estimated using the GEC v1.0 program (http://pmglab.top/gec/). After evaluating the LD decay distance, the loci associated with peak SNP, as well as the 100 kb flanking region and genes, were considered as candidates for further study. The location and type of SNP in the reference genome were identified using ANNOVAR software^69^. After merging SNPs genotype and annotation to HapMap formats, SNP haplotypes were identified in candidate genes using Candihap v1.3.0^70^ using the parameter “-m -u 2000 -d 500”.

**RNA-Seq and Gene Expression Analysis**

For the differential expression analysis of Tartary buckwheat response to NaCl treatment, germinated Pinku 1 seeds (a buckwheat variety whose genome was sequenced for genome assembly^71^) on soaked filter paper in a biochemical incubator for 3 days at 23℃ / 22℃, 16 hours light / 8 hours darkness. Seedlings with consistent growth (2 cm root length) were then selected and transferred to MS liquid medium for further cultivation until the leaf fully unfolded. Salt stress treatment was carried out on MS liquid medium containing 0.6% NaCl for 0 h, 3 h, 6 h and 12 h. The samples were then sent to Annoroad Gene Technology (Beijing, China) for transcriptome sequencing.

For the differential expression analysis of overexpression lines, *Arabidopsis* seeds of *FtMGT2* OE lines and 1302-GFP OE lines were sown on MS solid medium. The seeds were then vertically cultured in a biochemical incubator for 20 days at 23℃ / 22℃, 16 hours light / 8 hours darkness. Then, select *Arabidopsis* plants with consistent growth (just bolting) from *FtMGT2* OE lines and 1302-GFP OE lines and sent to Annoroad Gene Technology (Beijing, China) for transcriptome sequencing.

Total plant RNA of each sample was extracted using an RNA Extraction Kit (Aidlab, Beijing, China), and its quality was tested using the 2100 RNA Nano 6000 Assay Kit (Agilent Technologies, CA, USA). RNA was purified using Oligo (dT) and then digested with Fragmentation Buffer. To construct the RNA-Seq libraries, the first step involved amplifying complementary DNA (cDNA) using random primers. Then, the second-strand cDNA was synthesized using 2nd Strand Marking Buffer and 2nd Strand/End Repair Enzyme Mix. The constructed library was quantified using qPCR (Bio-RAD CFX 96, Bio-RAD KIT iQ SYBR GRN), and sequenced in Illumina platform with paired-end 150-bp mode. Adapter sequences and low-quality reads were eliminated from the initial reads. For the analysis of RNA-Seq, raw reads were initially filtered using the fastp v. 0.20 to remove adaptor and low-quality base. Subsequently, clean reads were aligned to the reference genome *F. tataricum*. Pinku (HERA) using HISAT2 (version 2.1.0) with default parameter settings and sorted by SAMtools v1.10^72^. The featureCounts program of Subread v.2.0.3^73^ obtained the genes count matrix and normalized gene FPKM values of each sample.

Differential expression analysis was performed using the R packages DESeq2 v1.38.3^74^. DEGs with the |log_2_ Fold Change|>=1 and adjusted Pvalue <=0.05 were identified in the control and treatment group. Additionally, gene expression level was performed time-course sequencing data analysis using R packages TCseq v1.22.6^75^ (<https://bioconductor.org/packages/release/bioc/vignettes/TCseq>) with the ‘fuzzy cmeans' mode and clusters was set at 15. Upregulated- and downregulated- DEGs were enriched in Cellular component (CC), Biological Process (BP) and Molecular Function (MF) of Gene Ontology and pathway of Kyoto Encyclopedia of Genes and Genomes (KEGG) using the Omicshare online platform (<https://www.omicshare.com/>). Co-expression analysis was referred to previous described^76^.

**Domestication Analysis**

The genetic differentiation index *F_ST_* between wild population and landraces were identified using the genomic_general (https://github.com/simonhmartin/genomics_general). To detect the candidate region possibly underwent selection in the process of domestication, wild population from Himalayan group (HW) was compared with the groups from Northern China (NL) and Southern China (SL), respectively. Selective sweeps among different groups were examined using the XP-CLR test with XP-CLR v1.1. Regions of the genome with the highest 5% XP-CLR values were identified as putative selected regions^5^.

**RNA-Fluorescence *in Situ* Hybridization (FISH)**

FISH experiments using 1-week-old untreated Tartary buckwheat seedlings and seedlings of the same age with 2-3 cm root tips that had been exposed to 100 mM NaCl stress for two days. Hybridization utilized the SweAMI ISH probe which synthesized and purified at Servicebio (Wuhan, China), with the probe sequence detailed in Supplementary Table 9. FISH assays were performed as previously described^77^.

**GUS Straining Assay**

The promoter sequences *FtMGT2* was amplified by PCR and then inserted into the pCAMBIA 1391 vector. The recombinant plasmids were introduced into *Agrobacterium* strain GV3101 and these were subsequently used to produce transgenic *Arabidopsis.* Then transgenic *Arabidopsis* seedlings were immersed in GUS staining solution and incubated at 25-37°C for 3 hours. Subsequently, the samples were de-stained in 70% ethanol 4-5 times until the negative control materials appeared white. Observation under the microscope revealed blue spots on a white background, indicating the sites of GUS expression. This assay was conducted using the GUS stain Kit (SL7160, Coolaber Tech Co., Ltd., China) in accordance with the instructions provided by the manufacturer. All primers utilized in the assay are documented in Supplementary Table 9.

**Knockout Vector Construction**

CRISPR-Cas9 vector construction were performed as previously described^78^. The potential off-target sites for our gRNAs were performed using the CRISPR Primer Designer tool against the Tartary buckwheat “Pinku1” genome, allowing for up to four mismatches. The analysis did not identify any potential off-target sites within the genome under these stringent parameters. Target sites were tailored according to the gene CDS region, with the finalized sgRNA expression cassette integrated into the CRISPR-Cas9 vector via the Golden Gate cloning technique. Then, we conducted the knockout vectors such as: CRISPR-Cas9-mgt2, CRISPR-Cas9-hkt1, CRISPR-Cas9-agl16, CRISPR-Cas9-myb15l, CRISPR-Cas9-brg1, CRISPR-Cas9-mgt2 / agl16 and CRISPR-Cas9-mgt2 /myb15l.

**Gene Cloning and Transgenic Hairy Roots and *Arabidopsis* Generation**

The CDS sequences of *FtMGT2* and *FtBRG1* were amplified by PCR and then inserted into the pCAMBIA 1302 vector. The *FtMYB15L,* *FtAGL16* and *FtHKT1* CDS sequence was amplified by PCR and subsequently inserted into the pCAMBIA 1307 vector. The primers and specific restriction sites are shown in Supplementary Table 9. The recombinant plasmid was transformed into *Agrobacterium* (*Agrobacterium rhizogenes*) A4 to generate transgenic hairy roots, following the established protocol described^5^. The recombinant plasmids were also introduced into *Agrobacterium* strain GV3101 to produce transgenic *Arabidopsis* using the floral dip method, as described by Clough^79^. The *mgt2* mutant (*SALK_006797C*) was obtained from Arashare, China; the *hkt1* mutant (*CS6531*) was obtained from TAIR; The mutants *mgt2* and *hkt1* were utilized as parents to obtain a homozygous double mutant, *hkt1*/ *mgt2*, by combining traditional hybridization techniques with PCR identification; the *hkt1*/ *FtMGT2* complementation line and the *hkt1*/ *FtHKT1* complementation line were produced using the floral dip method.

**Measurement of Flavonoid Contents**

The samples were dried at 105°C for 60 minutes and then placed at a constant temperature of 65°C until they reached a constant weight. 0.1 g of tissue was collected for each sample, ground into powder and sieved using a 60-mesh sieve. Then, 5 mL of 80% methanol was added to samples followed by sonication for 25 minutes at 50°C and 40 kHz. Extracts were filtered using a 0.22 μm nylon membrane filter and analyzed by HPLC-qToF-MS as previously described^5^. The flavonoid content was calculated by comparing it with the peak area of authentic standards using HPLC.

**Subcellular Localization**

Full-length CDS of *FtMGT2*, *FtMYB15L* and *FtBRG1* were cloned and inserted into the pCAMBIA1300-GFP vector. p2300-H2B-mCherry and pJIT-mCherry were used as the nuclear and membrane markers, respectively. The recombinant plasmid was transformed into *Agrobacterium* strain GV3101 and introduced into *nicotiana benthamiana* (*N. benthamiana*) leaves. Subcellular localization was examined utilizing a Zeiss LSM900 laser scanning confocal microscope. Primer sequences can be found in Supplementary Table 9.

**qRT-PCR Analysis**

To determine the expression of different haplotypes sample of *FtMGT2*, germinated different haplotype materials with approximately 10 seeds per material. 5 days after germination, the entire plant was frozen in liquid nitrogen and the total RNA was extracted.

To assess the expression of overexpressed materials in other phenotypic experiments, we collected materials from the phenotypic period and rapidly froze them in liquid nitrogen for subsequent total RNA extraction.

Total RNA extraction was performed using the RNA Easy Plant Tissue Kit (DP452, Tiangen, Beijing, China). Reverse transcription was conducted using the HiScript III RT SuperMix (+gDNA wiper) (R323, v21.1, Vazyme, Nanjing, China) according to the manufacturer's instructions. The quantitative reverse transcription polymerase chain reaction (qRT-PCR) was performed using the Taq Pro Universal SYBR qPCR Master Mix (Q712, v20.1, Vazyme, Nanjing, China) in accordance with the manufacturer's instructions. Primers are depicted in Supplementary Table 9.

**Construction and Phenotypic Experiment of Yeast Mutants**

*ΔKu70* is a non-homologous terminal junction-deficient strain of *Pichia pastoris*, which can enhance gene knockout and integration efficiency mediated by the CRISPR system^42^. Firstly, the corresponding gene PAS (Chr3_0706, named *MRS2*) of Pichia pastoris was obtained through Blast alignment based on the *MRS2* sequence derived from buckwheat. Its protein sequence is annotated as ‘Mitochondrial inner membrane Mg (^2+^) channel’. The endogenous *MRS2* gene of Pichia pastoris was knocked out using CRISPR gene editing technology. This was primarily achieved through the Cas9/gRNA system, which cleaved at the specific site of the MRS2 gene. Then, the MRS2 gene was repaired through homologous recombination using exogenous donor homologous fragments. After the repair, the MRS2 gene was completely deleted, and the mutant strain was named ΔMRS2. To prepare the ΔMRS2 strain into a receptive state, and then construct an MGT2 complement plasmid to transfer the *FtMGT2* gene from buckwheat into the ΔMRS2 defective strain, and obtained the strain ΔMRS2-MGT2. Using the same method, the sequence of the *FtMGT2* gene with a missing transmembrane domain was transformed into ΔMRS2, and ΔMRS2-mgt2 complementary yeast strain was obtained. All primers used are listed in Supplementary Table 9.

For the phenotypic experiment, first observe the growth of different yeast strains in YPD and YPD + Mg (14.9 g/L MgSO4, based on Invitrogen's Pichia Fermentation Process Guidelines) or YPD + 100 mM NaCl solid culture medium, YPD + 10 mM KCl or 50 mM KCl solid culture medium. On the other hand, various yeast strains were initially cultured in YPD + Mg liquid culture medium to a specific concentration. They were then diluted with fresh YPD liquid culture medium to ensure they all had the same concentration (OD600 = 0.1). The fresh inoculum was subsequently cultured in various liquid culture media at 30℃, 220 rpm, and the concentration was measured every hour.

**The Phenotype of Overexpression and Knockout Materials**

To phenotype the hairy roots, select fresh hairy roots with consistent growth of approximately 2-3cm. Culture them in 50 mL of MS liquid medium for two weeks at 20℃, 120 rpm^80^. Afterward, transfer them to MS liquid medium and MS medium containing 100 mM NaCl for another two weeks. Then, take photos and measure the fresh weight and dry weight. Use the fresh hairy roots to measure the enzyme activity of superoxide dismutase (SOD) and catalase (CAT) according to the manufacturer's instructions (Superoxide Dismutase (SOD) Activity Assay Kit, BC0175, Solarbio, China; Catalase (CAT) Activity Assay Kit, BC0205, Solarbio, China), and use the drought hairy roots to measure the flavonoid content.

To phenotype the *Arabidopsis*, seeds of *FtMGT2* OE lines, *FtMYB15L* OE lines and Col-0 were sown in MS solid medium, then vertically cultured in the biochemical incubator for 3 days at 23℃ / 22℃, 16 hours light /8 hours darkness, and then selected neat and consistent seedlings and transferred them to MS solid medium with and without 100 mM NaCl. When the root length in MS medium without NaCl reaches the bottom, the root length of *Arabidopsis* was measured at various concentrations. Additionally, transplanted *FtMGT2* OE lines and Col-0 *Arabidopsis* each 50 seedlings in MS liquid medium with different concentrations of NaCl (50 mM and 100 mM), respectively, incubate in the dark for 3 weeks (18℃, 120 rpm), then take photos and measure the fresh weight and dry weight.

To observe the growth and development of *FtMGT2* overexpression *Arabidopsis*, we planted both the *FtMGT2* overexpression *Arabidopsis* and Col-0 in nutrient soil under suitable cultivation conditions (23℃ / 22℃, 16 hours light / 8 hours darkness), then observed the bolting time, flowering time, and the number of rosettes leaves at flowering. Using newly bolted *Arabidopsis* leaves for disease tolerance identification, the methods referred to the previous article^81^.

To investigate the expression of *FtMYB15L* overexpression *Arabidopsis* under salt tolerance, seeds of *FtMYB15L* OE lines and Col-0 were sown in MS solid medium, and then vertically cultured in a biochemical incubator for 10 days at 23℃/ 22℃, 16 hours light/ 8 hours darkness. Then, place the *FtMYB15L* OE lines and Col-0 *Arabidopsis* in a new MS liquid medium and MS with 100 mM NaCl. Gently shake the samples for 2, 3 and 4 hours. Afterward, use the samples of 3 hours to measure the expression of *FtMYB15L*, and use the samples from the 2 and 4 hours to measure the accumulation of FtMYB15L protein, then extract the protein by the Plant Protein Extraction Kit (BC3720, Solarbio, China), and immunoblotted with anti-MYC antibodies (Anti c-Myc Mouse Monoclonal Antibody, CW0299, Cwbio, China).

**Treatment of Plants under Salt Stress with Kaempferol**

After disinfecting the Pinku 1 seeds (1% NaClO for 8 minutes and 75% ethanol for 8 minutes), seeding and cultured them in MS solid medium with 100 mM NaCl, 0.01 mg/mL kaempferol + 100 mM NaCl and 1 mg/ mL kaempferol + 100 mM NaCl. After 7 days, observe the phenotype of Pinku 1 in various culture media and measure the length of the roots.

After disinfecting the Col-0 *Arabidopsis* seeds (using 75% ethanol for 8 minutes, followed by ethanol for another 8 minutes), seeding and cultured them in MS solid medium with 100 mM NaCl, 0.01 mg/mL kaempferol + 100 mM NaCl and 1 mg/mL kaempferol + 100 mM NaCl. After 7 days, the phenotype of Pinku 1 in different culture media was observed and the root length measured.

**Yeast One-Hybrid Assay**

The promoter of the *FtMGT2* gene was integrated into the pHis vector for use as a reporter. The *FtMYB15L* CDS was introduced into the pGADT7 vector, which harbors a GAL4 transcriptional activation domain as an effector. The effectors were introduced into the Y1H gold strain, with each strain harboring the reporter gene. Transformants were cultured on a minimal synthetic defined (SD)-glucose medium that did not contain Leu (-L) and Trp (-T). The Y1H assay was conducted in accordance with the manufacturer's instructions (Matchmaker Y1H System; Clontech, USA). All primers utilized in the assay are documented in Supplementary Table 9.

**ChIP-qPCR**

Perform ChIP-qPCR experiments using *FtMYB15L* or *FtAGL16* overexpressing hairy roots as previously described^82^, with sample lysis and immunoprecipitation was performed using the Chromatin Immunoprecipitation (ChIP) Kit For Plant (JKR23002P, GENECREATE BIOLOGICALENGINEERING CO., LTD. Wuhan, China) in accordance with the manufacturer's instructions.

**Yeast Two-Hybrid Assay**

The coding sequence regions of *FtMYB15L*, *FtMYB15Lc* and *FtMYB15Ln* were individually integrated into the pGADT7 vector using homologous recombination. The full-length sequences of *FtAGL16*, *FtBRG1*, *FtBRG1c* and *FtBRG1n* were inserted into the pGBKT7 vector using homologous recombination. Yeast transformation was conducted in accordance with the guidelines provided by the manufacturer (Clontech, USA). The bait and prey vectors were simultaneously introduced into the yeast strain Y2H and cultivated together on a selective SD medium that did not contain Leu and Trp. After an incubation period of 3 to 4 days at a temperature of 30 °C, the yeast cells were applied in dilutions of 10- and 100-fold onto selection plates that were supplemented with an SD medium lacking Leu, Trp, and His. The plates were incubated at a temperature of 30 °C until colonies of yeast cells were observed. All primers utilized in the assay are documented in Supplementary Table 9.

**EMSA Assay**

The EMSA experiments were conducted following the methodology described^47^. The complete coding sequence of *FtMYB15L* was inserted into the pET28A vector and the complete coding sequence of *FtAGL16* was inserted into the pGEX-4T-2 vector through homologous recombination and subsequently introduced into *Escherichia coli* strain Arctic-Express (DE3) for the purpose of expressing the His-FtMYB15L protein and GST-FtAGL16 protein. The recombinant proteins were subjected to affinity purification using Ni-beads or the GST beads fusion protein purification^46^(Sangon Biotech, China). Oligonucleotide probes (Supplementary Table 9) were synthesized and biotinylated at the 5' end by Sangon Biotech. EMSA was conducted using the LightShift® Chemiluminescent EMSA Kit (Thermo scientific, USA) in accordance with the instructions provided by the manufacturer. All primers utilized in the assay are documented in Supplementary Table 9.

**Firefly Luciferase Fragment Complementary Imaging Technology (LCI) Assay**

For LCI assays, the full length of *FtMYB15L, FtBRG1* and *FtAGL16* was amplified using specific primers and introduced into pCAMBIA1300-cLUC and pCAMBIA1300-nLUC. The recombinant vectors were transformed into GV3101 and then co-transformed into the 4-week-old *N. benthamiana* leaves. Using the living fluorescence imager to observe fluorescence and measure LUC activity. All primers

utilized in the assay are documented in Supplementary Table 9.

**Pull-Down Assay**

The complete coding sequence of *FtAGL16* and *FtBRG1* was successfully inserted into the pGEX-4T-2 by homologous recombination. The resulting construct was then transformed into Escherichia coli strain (DE3) for the purpose of expressing the GST-FtAGL16 protein and GST-FtBRG1 protein. In vitro-expressed and purified GST fusion proteins, specifically GST-FtBRG1 or GST-FtAGL16 and the negative control GST. Subsequently, His-FtMYB15L fusion proteins were introduced and incubated for an additional 3 hours at a temperature of 4°C. After undergoing five washes with the pull-down buffer, the GST beads that had precipitated were gathered through a short centrifugation process (2,000 *g*, 2 min) and subsequently reconstituted in the protein extraction buffer. Proteins were separated using sodium dodecyl sulfate-polyacrylamide gel electrophoresis (SDS-PAGE) and subsequently detected using specific antibodies. The Anti His-Tag Mouse Monoclonal Antibody (CW0286, Cwbio, China) and Anti GST-Tag Mouse Monoclonal Antibody (CW0884, Cwbio, China) were employed for this purpose. All primers utilized in the assay are documented in Supplementary Table 9.

**BiFC Assay**

The pEARLY-FtMYB15L-YFPc, pEARLY-FtAGL16-YFPn, and pEARLY-FtBRG1-YFPn vectors are assembled through the Golden Gate cloning technique, subsequently introduced into *Agrobacterium* GV3101, then transferred into tobacco leaves. Observed the subcellular localization using laser scanning confocal microscopy^81^.

**Co-IP Assay**

Co-IP were performed using *FtMYB15L* overexpressing hairy roots as previously described^82^, with sample lysis and immunoprecipitation was performed using the Co-Immunoprecipitation (Co-IP) Kit for Plant (JKR23001P, GENECREATE BIOLOGICALENGINEERING CO., LTD. Wuhan, China) in accordance with the manufacturer's instructions.

**Element Analysis with ICP-MS**

The samples are dried in an oven to achieve a constant weight, then ground and precisely weighed to 0.2 g for ionic analysis conducted, following high-temperature digestion of the sample, it is introduced in liquid aerosol form, ionized in the plasma, separated by the mass spectrometer, and ultimately detected by the ion detection system^83^.

**Dual-Luciferase Assay**

The 62SK-FtMYB15L-His and 62SK-FtBRG1 effectors (FtMYB15L-His and FtBRG1 inserted into the pGREEN62SK vector) and the *FtMGT2*pro-LUC reporter constructs (the *FtMGT2* promoter inserted into the pGREEN0800-miniLUC vectors) were introduced into GV3101 and co-injected into *N. benthamiana* leaves were then cultured for a period of 2 days. Using the living fluorescence imager to observe fluorescence and quantify LUC/REN activity. Additionally, after observing the fluorescence, extract the protein of 62SK-FtMYB15L-His / *FtMGT2*pro-LUC and the protein of 62SK-FtMYB15L-His / *FtMGT2*pro-LUC by the Plant Protein Extraction Kit (BC3720, Solarbio, China), and use the anti-His to measure the accumulation of FtMYB15L protein. ACTIN was used as the internal control and immunoblotted with anti-Actin antibodies (Anti Actin Mouse Monoclonal Antibody, CW0264, Cwbio, China). The Western blot methods can be referred to in the previous article^81^. All primers utilized in the assay are documented in Supplementary Table 9.

***In Vivo* Ubiquitination Assay**

To verify the ubiquitination interaction between FtBRG1 and FtMYB15L1, we transient transformed 4-week-old *N. benthamiana* leaves using the GV3101 strains of FtMYB15L-Myc and FtBRG1-GFP (the first group is FtMYB15L-Myc, the second group is FtMYB15L-Myc + FtBRG1-GFP). Then extract the protein by the Plant Protein Extraction Kit (BC3720, Solarbio, China), and immunoblotted with anti-MYC antibodies (Anti c-Myc Mouse Monoclonal Antibody, CW0299, Cwbio, China) and anti-Ub antibodies (Ubiquitin (NT) Mouse Monoclonal Antibody, AG3157, Beyotime, China).

**Protein Degradation Assay**

Protein degradation assay was performed as previously described^84^, the constructs *FtMYB15L*-Myc and *FtBRG1*-GFP were individually transfected into GV3101 strains, and co-infiltrated into tobacco leaves as indicated. Total FtMYB15L-Myc proteins were extracted then analyzed by SDS-PAGE and detected by immunoblotting with anti-Myc. The relative protein level of immunoblot assay was quantified by ImageJ.

**Non-Invasive Ion Flux Measurements (NMT)**

Fluxes of Na^+^ and Mg^2+^ were measured from root tissues using non-invasive microelectrode ion fluxes measuring system. Full details of microelectrodes fabrication and calibration and principles of the MIFE ion flux measurements are according to the instructions provided by Xuyue Technology Co., Ltd. Beijing, China: Mg^2+^ fluxes were measured by the NMT (NMT100S-SIM-XY, Xuyue, Beijing, China). Plants were put into Mg test solution (0.1 mM MgCl_2_, pH = 6), pre-pulled and salinized microelectrodes (NMT-HC-03 Xuyue, China) were filled with approximately 1.0 cm of backfilling solution (500 mM MgCl_2_) and then attached with a selective liquid Mg^2+^ exchange cocktail (LIXs, Mg^2+^, XY-STZ-Mg-T, Xuyue, China) for Mg^2+^ detection. Na^+^ fluxes were measured by the NMT (NMT100S-SIM-XY, Xuyue, Beijing, China). Plants were put into Na test solution (1 mM NaCl, pH = 6), pre-pulled and salinized microelectrodes (NMT-HC-03 Xuyue, China) were filled with approximately 1.0 cm of backfilling solution (500 mM NaCl) and then attached with a selective liquid Na ion-exchange cocktail (LIXs, Na^+^, XY-STZ-Na-T, Xuyue, China) for Na^+^ detection.

***Salmonella* Mutant MM281 Uptake Assay**

The *Salmonella* mutant MM281 uptake assays performed as previously described^16^, use the MM1927 as the positive control, and MM281 and MM281 transformed with the empty pTrc99A vector as two negative controls. Growth in different concentrations of MgSO_4_.

**Two-Electrode Voltage Clamping (TEVC)**

The TEVC assays performed as previously described^22^. All *Xenopus laevis* oocytes (Stage IV, female) used in this research were purchased from the commercial supplier Washtenaw Frog and Reptile Farm (Brownstown, MI, USA). The cDNAs of *FtMGT2* and *FtHKT1* were cloned into pGEMHE vectors. Transcription and capped RNAs were prepared from the linearized pGEMHE vector DNA templates using the mMACHINE high-yield capped RNA T7 kit following the manufacturer’s instructions (Themo, Ambio). The cRNAs at concentrations of 500 ng/μl were divided into 0.2-ml PCR tubes and stored at -80℃ until injection. Xenopus oocytes were harvested at stages V to VI and incubated in ND96 (without Ca^2+^) solution with 1.5 mg/ml clostridiopeptidase A at 26℃ and 40 rpm for 3 h of enzymolysis, then washed seven times and maintained in ND96 solution overnight prior to injection at 18℃. Oocytes were injected with different cRNAs. For recording of *FtMGT2* and *FtHKT1*, 23 nl of 250 ng/μl cRNA solution, respectively, was injected into one oocyte. For recording of *FtMGT2* + *FtHKT1*, a 23 nl mixture of two cRNAs (500 ng/μl for *FtMGT2* and *FtHKT1*) was injected into one oocyte. The whole oocyte current was recorded using the TEVC 1 days after cRNA injection with a GeneClamp 200B amplifier (Axon Instruments). Voltage-pulse protocols, data acquisition, and analysis were performed using pClamp9 software (Axon instruments). Electrodes were filled with 3 M KCl. The oocytes were continuously perfused during the voltage-clamp experiment. Channel recordings were initiated in bath solutions (10 mM HEPES–NaOH [pH 7.4]), which were supplemented with 20 mM MgCl_2_, 20 mM NaCl, 20 mM KCl, 2 mM NaCl, 2 mM NaCl + 0.1 mM MgCl_2_, 2 mM NaCl + 10 mM MgCl_2_, 2 mM NaCl + 0.1 mM CaCl_2_ or 2 mM NaCl + 10 mM CaCl_2_ and adjusted osmotically to 220 mmol / kg with mannitol. Voltage steps were applied from 0 to -140 mV in 10 mV increments during 2.2 s, each beginning with 0.2 s and ending with 0.6 s at the resting potential of the oocyte membrane in the tested bath solution. Gap-free voltage was applied to the oocyte membrane in the tested bath solution containing 220 mM mannitol and 2 mM NaCl at a holding potential of -60 mV. Solutions of MgCl_2_ or CaCl_2_ substrates in the same solution were prepared and the pH adjusted to 7.4.
